# A review of gorgonian coral species (Cnidaria, Octocorallia, Alcyonacea) held in the Santa Barbara Museum of Natural History research collection: focus on species from Scleraxonia, Holaxonia, Calcaxonia – Part II: Species of Holaxonia, families Gorgoniidae and Plexauridae

**DOI:** 10.3897/zookeys.860.33597

**Published:** 2019-07-04

**Authors:** Elizabeth Anne Horvath

**Affiliations:** 1 Westmont College, 955 La Paz Road, Santa Barbara, California 93108, USA Westmont College Santa Barbara United States of America; 2 Invertebrate Laboratory, Santa Barbara Museum of Natural History, 2559 Puesta del Sol Road, Santa Barbara, California 93105, USA Santa Barbara Museum of Natural History Santa Barbara United States of America

**Keywords:** Allan Hancock Foundation (AHF) – ‘Velero’ Expeditions, cryptic species, local endemics, museum collection, new *Eugorgia* species, *
Placogorgia
*, “red whips”, soft corals, “thread-like” forms

## Abstract

Gorgonian coral specimens from the Holaxonia, families Gorgoniidae and Plexauridae held in the collection of the Santa Barbara Museum of Natural History (SBMNH) were reviewed and evaluated for species identification. The specimens were collected from within, and adjacent areas of, the California Bight. The SBMNH collection has encompassed within it a large percentage of specimens collected by the Allan Hancock Foundation (AHF) ‘Velero’ Expeditions of 1931–1941 and 1948–1985. This historic collection displays an emphasis on species belonging to the Holaxonia, particularly the gorgoniids and plexaurids; thus, this second part presents a thorough discussion of well-known genera from within the California Bight, with more extensive discussions of several genera that have historically, and currently, led to confusion (and thus, misidentification). A brief discussion of a California Bight grouping, referred to within as the “red whips,” is presented; this grouping encompasses several species with very similar colony appearance across a number of genera. Two species, the gorgoniid *Leptogorgiachilensis* (Verrill, 1868) and the plexaurid *Chromoplexauramarki* (Kükenthal, 1913) each required the designation of a neotype from within the collection. A new species in the genus *Eugorgia* Verrill, 1868, a whip or thread-like form belonging to the family Gorgoniidae, is described. One additional plexaurid genus (*Placogorgia*) is discussed, a genus not commonly reported for the California Bight region. This is the first comprehensive work, in three parts, focusing on all species of gorgonian coral known to inhabit the California Bight. This paper, Part II of the full work, continues the systematic review of all species represented in the Santa Barbara Museum of Natural History research collection begun in Part I.

## Introduction

As defined in Part I of this work, the term gorgonian used in this paper refers to those alcyonacean octocorals belonging to the groups Scleraxonia, Holaxonia and Calcaxonia. These organisms are modular colonies, usually extensively branched, displaying a stiff central, internal axis (composed of calcite and gorgonin), in both main stem and all branches, composed of either fused sclerites, or sclerites composed of scleroproteinous gorgonin. The entire axial skeleton is covered with soft tissue coenenchyme filled with numerous calcareous sclerites, either embedded in it or lying on its surface. The supporting axial skeleton allows for colonies to achieve large size (some species) and allows for the display of both highly branched colonies, known as sea fans, as well as long, slender forms known as sea whips.

The gorgonian Holaxonia are the most numerous of the gorgonian corals found in the Santa Barbara Museum of Natural History’s (SBMNH) research collection. While a fair number of specimens, representing the families Gorgoniidae and Plexauridae were already present, with the acquisition of gorgonian materials from the Allan Hancock Foundation ‘Velero’ Expeditions, the holdings within the collection were greatly enhanced. Many of the specimens not only needed rebottling, but extensive work had to be done to identify (or to correct identification of), not only the specimens that were already in the collection, but the many specimens collected during the ‘Velero’ years of operation. As many of the genera in these two families have been thoroughly reviewed elsewhere (Breedy and Guzmán 2007, 2016, 2018, Breedy et al. 2009), I am providing only brief descriptions for most. More problematic genera, or genera and species that have not been well studied, are given more extensive coverage and discussion. Not all of the Holaxonia holdings in the SBMNH collection are covered here. Part III of this review will cover two of the genera that were most in need of work and required more thorough discussion.

## Materials and methods

Nearly all of the specimens examined in this work (housed currently as part of the Santa Barbara Museum of Natural History’s permanent research collection, Invertebrate Laboratory), were collected over a period of years dating from the 1930s to the present, in either dry or wet condition. A large percentage of these specimens came to the SBMNH through a diverse 10,000-lot cnidarian collection, a portion of the Allan Hancock Foundation (AHF) collection built upon the historic ‘Velero’ expeditions of 1931–1941 and 1948–1985. Not only are gorgonian specimens housed in the cnidarian section of the entire invertebrate collection, but there are gorgonians housed elsewhere within the collection; for instance, gorgonian coral fragments are housed in the museum’s mollusk collection (the mollusks in question were found on, and collected with, a species of gorgonian), or in other sections of the museum’s cnidarian collection (such as zoanthid anemones collected on gorgonian corals). Scattered throughout other portions of the museum’s invertebrate collection are bryozoans, barnacles, or brittle stars that were collected from within or on gorgonian coral colonies and were preserved with the gorgonian they were living with. To assist with the identification of the SBMNH specimens, examinations of specimens of known species from or collected in the Bight were performed on material found in the collections of the National Museum of Natural History, Smithsonian (**USNM** = NMNH), the California Academy of Sciences, San Francisco (**CAS**), the Los Angeles County Museum of Natural History (**LACoMNH**), Scripps Institute of Oceanography (**SIO**), the Monterey Bay Aquarium Research Institute (**MBARI**), Moss Landing Marine Laboratories (**MLML**) and the small museum which is a part of the Cabrillo Marine Aquarium in San Pedro, California (**CMA**) (see Appendix 1: List of material examined). These were compared to SBMNH specimens, informing the identification of species represented in the SBMNH collection. Additionally, several National Oceanographic and Atmospheric Administration (NOAA) offices throughout the country provided further material for study.

All specimens were examined for gross colony morphology; more importantly, examination of the calcareous sclerites, present in different parts of the colony, was conducted for nearly all specimens. The standard method for sclerite extraction (tissue sample in common household bleach) was performed, and light microscopy via a compound Olympus (CH) microscope, was used initially to determine the genus to which a specimen belonged. Scanning Electron Microscopy (SEM) of the sclerites was then undertaken. All samples were coated with gold, using a Cressington Sputter Coater Unit, 108auto. Samples were examined and digital images taken, using a Zeiss Scanning Electron Microscope EVO 40, at 10 kV. This second part covers some fourteen species, classified as holaxonians belonging to the families Gorgoniidae and Plexauridae. A summative overview of species housed in the SBMNH research collection, from these specific groups, is included below.

This information regarding species and lots of specimens examined for Part II for colony morphology and sclerites (either through light microscopy or SEM) is a summation of the more detailed information to be found in the Appendix 1: List of material examined – Part II. It is evident from this summative overview that the SBMNH research collection illustrates diversity and abundance of species from the holoaxonian group found within or near the California Bight.

**Table:** Part II: Collective specimen and species data.

# of specimens analyzed with sclerite preparations	~260
# of specimens examined without sclerite preparation	0
Breakdown of specimens examined:	
# of specimens analyzed from SBMNH collection	~184
# of specimens analyzed from USNM-Smithsonian	19
# of specimens analyzed from CAS	13
# of specimens analyzed from other institutions	54
Total # of species that received sclerite observations	14
# of new species described	1
Breakdown of species examined:	
# of species from the SBMNH collection	14
# of species from USNM-Smithsonian	7
# of species from CAS	3
# of species from other sources	10
# of species shown in Figures (colony)	13
# of species shown in Figures (either light microscopy and/or SEM of sclerites)	14

**Table:** Species covered in this part.

	**SBMNH**	**Other institutions**	**Colony figure**	**Sclerite figure**
* Adelogorgia phyllosclera *	Yes	Yes	Yes	Yes
* Eugorgia daniana *	Yes	Yes	Yes	Yes
* Eugorgia rubens *	Yes	Yes	Yes	Yes
*Eugorgialjubenkovia* sp. nov.	Yes	Yes	Yes	Yes
* Leptogorgia chilensis *	Yes	Yes	Yes	Yes
* Leptogorgia diffusa *	Yes	Yes	Yes	Yes
* Leptogorgia filicrispa *	Yes	Yes	Yes	Yes
* Leptogorgia flexilis *	Yes	Yes	Yes	Yes
*Leptogorgia* sp. A	Yes	Yes	Yes	Yes
* Chromoplexaura marki *	Yes	Yes	Yes	Yes
* Muricea californica *	Yes	Yes	Yes	Yes
* Muricea plantaginea *	No	Yes	No	Yes
* Muricea fruticosa *	Yes	Yes	Yes	Yes
*Placogorgia* sp. A	No	?	Yes	Yes

## Systematic accounts

(Classification used throughout this paper conforms to that of Bayer 1981c)

### Diagnosis of the Order Alcyonacea Lamouroux, 1816

(Gorgonian corals, as defined previously)

Octocorals with uniformly short gastrovascular cavities; colonies typically arborescent, rarely lobate or incrusting, producing more or less specialized three-dimensional axial skeletal structures: either a distinct central axis of horny (gorgonin) or calcareous material (or both), or a central medullar zone of calcareous sclerites which are loosely or inseparably bound together by horny or calcareous matter.

### Holaxonia Studer, 1887

With distinct central axis composed of horny material alone or of horny material more or less heavily permeated with calcareous substance, continuous or with alternating horny and calcareous joints. In center of axis is a relatively narrow, largely hollow, tubular space partitioned into series of small chambers, referred to as the cross-chambered central chord. Calcareous material of the peripheral zone of axis is in nonscleritic form (single exception in Keroeididae).

### Key to Families represented in SBMNH collection (Holaxonia)

**Table:** 

1	Axis horny, with a chambered, hollow, soft central chord	**2**
–	Axis not horny, but is a solid axis, with no soft, central, hollow core	**See Calcaxonia, Part III**
2	Axis purely horny, composed of scleroprotein, without any calcareous deposits	**3**
–	Axis horny, but some calcareous material may be present in some forms; hollow, horny, soft-chambered central chord is wide; there is a peripheral zone of hollow, horny spaces containing calcareous material; cortex is thick, with an inner and outer layer, formed by systematic longitudinal canals; polyps retractile into prominent calyces	**Family Plexauridae**
3	Axis perforated by a wide, cross-chambered central chord; cortex thin; polyps not retractile; sclerites spikey and conspicuous	**Family Acanthogorgiidae** (covered in Part I)
–	Distinct hollow, horny, soft-chambered central chord that perforates axis is narrow; axial cortex surrounding the core is very dense; polyps fully retractile, into low calyces	**Family Gorgoniidae**

### List of species

Class Anthozoa

Subclass Octocorallia Haeckel, 1866

Order Alcyonacea Lamouroux, 1816

Holaxonia Studer, 1887

Family Gorgoniidae Lamouroux, 1812

*Adelogorgiaphyllosclera* Bayer, 1958

*Eugorgiadaniana* Verrill, 1868

*Eugorgiarubens* Verrill, 1868

*Eugorgialjubenkovia* sp. nov.

*Leptogorgiachilensis* (Verrill, 1868)

*Leptogorgiadiffusa* (Verrill, 1868)

*Leptogorgiafilicrispa* Horvath, 2011

*Leptogorgiaflexilis* (Verrill, 1868)

*Leptogorgia* species A [? = *Leptogorgiatricorata* Breedy & Cortés, 2011]

Family Plexauridae Gray, 1859 [= Muricidae]

*Chromoplexauramarki* (Kükenthal, 1913)

Discussion concerning diversity of “red whip” forms

*Muriceacalifornica* Aurivillius, 1931

*Muriceaplantaginea* (Valenciennes, 1846) = *M.appressa* Verrill, 1864

*Muriceafruticosa* Verrill, 1868

(following genus formerly part of: [Stenogorgiinae = old Paramuriceidae]

*Placogorgia* species A

### Descriptions of species

#### Family Gorgoniidae

Taxon classificationAnimaliaAlcyonaceaGorgoniidae

Lamouroux, 1812

##### Diagnosis.

Axis purely horny, composed of carbonate hydroxylapatite with narrow but distinct chambered central chord; cortex little loculated, if at all. Polyps fully retractile, some forming low calyces (polyp-mounds), scattered or biserially disposed. Axis/polyp coenenchyme moderately thick, packed with spindles and capstans with regular belts of tubercles; in certain genera modified into disc spindles, scaphoids, or unilaterally spinous forms. Anthocodial armature weak, in form of crown composed of flat rodlets with scalloped edges, or lacking entirely. Colonies of diverse form, from unbranched to pinnate, closely reticulate or foliate.

#### Genus Adelogorgia

Taxon classificationAnimaliaAlcyonaceaGorgoniidae

Bayer, 1958

Adelogorgia Bayer, 1958: 46; 1979: 1026–1027. Breedy and Guzmán 2018: 329.

##### Type species.

*Adelogorgiaphyllosclera* Bayer, 1958.

##### Diagnosis.

Genus originally included in family Plexauridae (Bayer 1958). Presence of moderately thick coenenchyme; polyps communicate directly with system of longitudinal canals. Exterior coenenchyme contains derivatives of short, stout capstans called double wheels/discs; large, leaf-like expansions (on one side) up to 0.15 mm long; spindles with tubercles in transverse rows, to 0.2 mm, some developed as leaf clubs. Interior layers of coenenchyme contain only spindles. Anthocodiae weakly to moderately armed with flat rods, 0.15–0.3 mm long.

##### Etymology.

*Adelo-* is Greek for unknown. When Bayer described this genus in 1958 it was a new, unknown gorgonian genus; however, Bayer did not discuss the derivation.

#### Adelogorgia phyllosclera

Taxon classificationAnimaliaAlcyonaceaGorgoniidae

Bayer, 1958

**Figure 1.**
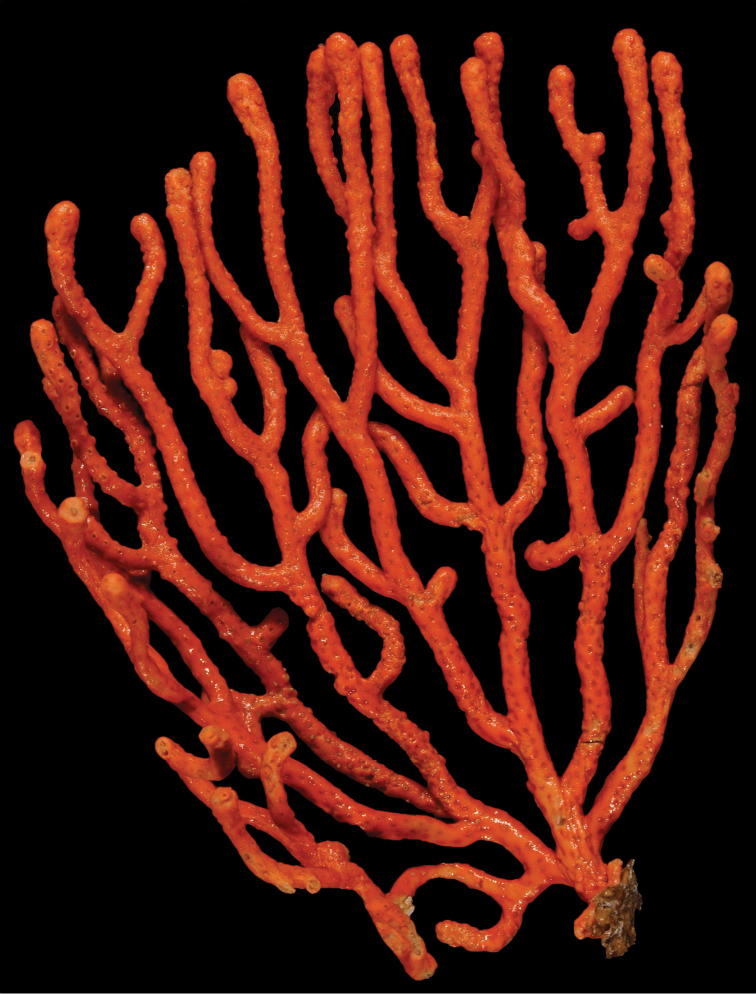
*Adelogorgiaphyllosclera*, SBMNH 51252. Colony measures 16.5 cm × 15.0 cm.

**Figure 2.**
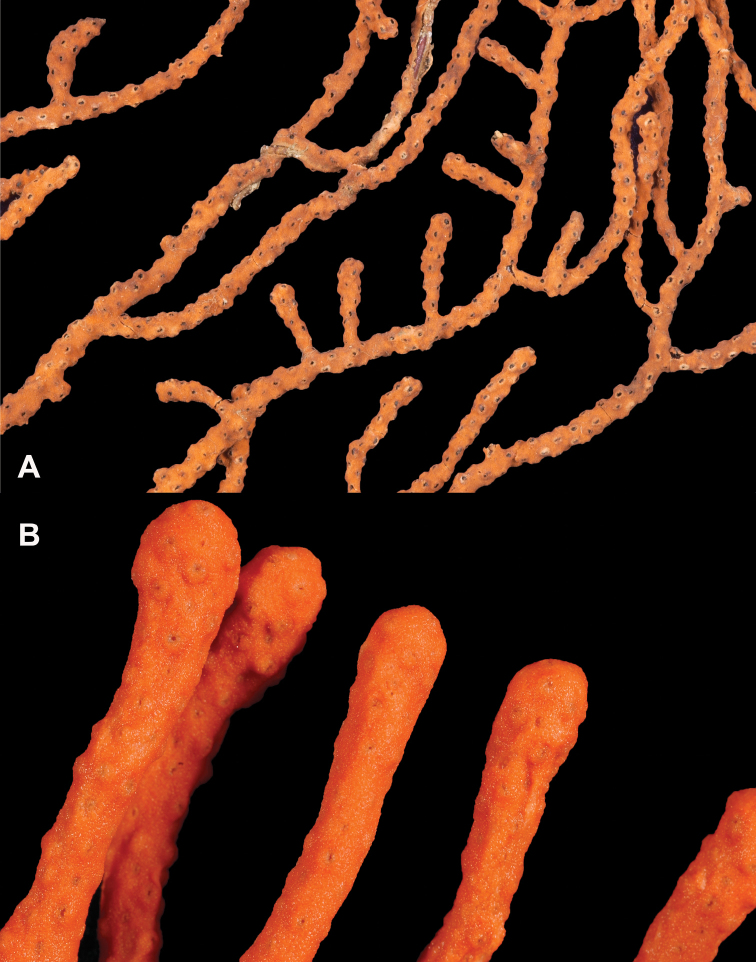
*Adelogorgiaphyllosclera*. **A**SBMNH 422403 [dry], slightly magnified image of branches **B**SBMNH 51252 [wet], rounded, slightly club-shaped branch tips.

**Figure 3.**
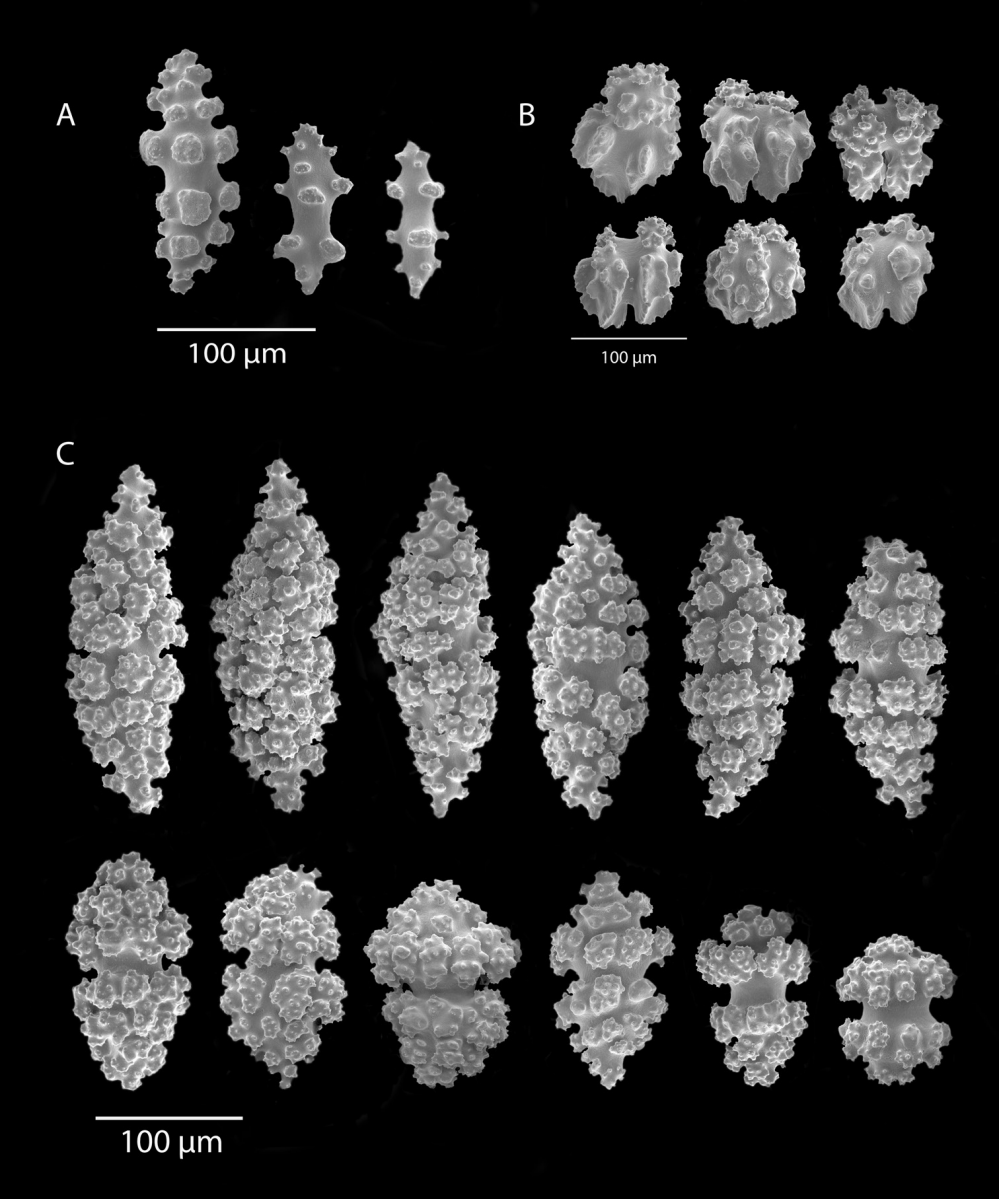
*Adelogorgiaphyllosclera*, SBMNH 51252, SEM image. Sclerites red-orange in color. **A** Short warted spindles (middle and right, potentially anthocodial sclerites) **B** Characteristic “leaf scale” sclerites **C** Warted coenenchymal spindles. Compare leaf scales shown here to those seen in Bayer 1979 (figs 5c, 6c).

Figures 1 , 2A, B , 3A–C

Adelogorgia phyllosclera Bayer, 1958: 46–47; figs 3a–f, 4a, b, 9b, c. Breedy and Guzmán 2018: 330–333.

##### Type locality.

USA, California, La Jolla, South of Scripps Institution, La Jolla Canyon, 30–33 m.

##### Type specimens.

**Holotype**USNM 50186; [dry]. **Paratypes** listed under “other material” in “Appendix 1: List of material examined” for this species.

##### Material examined.

~26 lots (see Appendix 1: List of material examined). All specimens at USNM were examined for comparison purposes.

##### Description.

*Colony* (Figure 1) heavy; bushy to fan-shaped, branching strictly in single plane, particularly in young colonies (≤ 20 cm height), but with branches occasionally growing irregularly as colony gets older (up to 0.6 m in height, usually less than 0.3 m); branching dichotomous, irregular and lateral, not pinnate (Figure 2A), with knobby to smooth branches. Color of live colony red or orange-red, polyps yellow to yellow-orange; axis slender, orange. Dry specimens brilliant red, rusty red, maroon-rust to black. Branches 2.0–4.5 mm in diameter, ascending into a meandering, sinuous form; terminal branches short (3.0–4.0 cm), slightly swollen at distal ends (Figure 2B). Trunk diameter measures up to ~6.0 mm. Axis in dead/dry specimens, within all branches of older part of colony, black, smooth, without conspicuous striations; in branches of younger portions of colony, maroon. Outer layer of axis abundantly loculate, texture of axis weak, flexible in terminal portion of branches, rather brittle in base area. Polyps with weak operculum, composed of two to four curved spindles in every segment, arranged en chevron; sclerites in polyps not arranged transversely, not forming collaret. Polyps able to retract down to surface or upper marginal edge of low to moderate calyces, these moderately elevated as low bumps off branch surface (0.5–0.8 mm tall, 1.2 mm across), situated some 2.0–2.5 mm distance apart, distributed over entire surface of all branches; margin of calyces not dentate. Sclerites (Figure 3) of polyps straight or curved rods, sculptured with simple conical warts, arranged en chevron, two to four sclerites at base of each tentacle. Coenenchyme spiculation in two layers; layers determined by shape of sclerites seen in each. Exterior layers of coenenchyme with stout capstans (0.1 mm), and spindles (0.2 mm); (latter more common), some less commonly present as having leaves or scales over one surface; few appear as leaf clubs. Numerous sclerites with sculpturing on one side modified into leafy projections (double-discs) as seen in Figure 3B (appear occasionally as sclerites analogous to disc-spindles of *Eugorgia*); in many cases, sclerites (0.1–0.15 mm) strongly characteristic of this form; proportion of leafy sclerites to ordinary ones in outer layer of coenenchyme varies only slightly. Ordinary ones are most abundant, while leafy ones, though sometimes rare, are always present. Axial sheath (interior layer of coenenchyme) contains symmetrical spindles (0.16 mm) only; no capstans, clubs or leafy scales. Sclerites of outer layer red; of inner layer nearly colorless.

##### Etymology.

The root *phyllo-* (Greek) = leaf; *sclero-* (Greek) = hard scale. Species is unusual in the leafy appearance of one sclerite type, a key characteristic in identifying the species. However, Bayer gave no explanation for either genus or species names.

##### Common name.

Chuck’s gorgonian; Orange gorgonian; Shady-leafed gorgonian; Hard-leaved gorgonian; Hidden gorgon (these names appear in a variety of field/diving guides for the area such as Gotshall 2005, Kerstitch and Bertsch 2007).

##### Distribution.

Based on collection location data, from Upper Baja, California to southern California. Generally known from La Jolla area of southern California. One specimen from Catalina Island, Bird Rock, SBMNH 51252 (one of several from Santa Catalina Island), indicates this species does range a bit north of La Jolla, California.

##### Biology.

Commonly encountered in southern California in kelp beds; depth range of 20–300 m (Gotshall 2005). An anecdotal note (J Ljubenkov, penciled notation on a species list) stated: “*Adelogorgiaphyllosclera* is a deep water form; it is a major deep water gorgonian and replaces *Muricea* on sewage pipes” (verified by staff of LACSD and OCSD). Two specimens, USNM 50186 and SBMNH 422894 (Point Loma), support numerous epizootic anemones (perhaps *Epizoanthus* Gray, 1867). On others, a flat, grayish incrustation (perhaps bryozoan) can be seen. Some balanoid barnacles are present over the surface of some specimens examined, in the form of prominent cysts (“galls”) on the branches, which protrude out from the axis through a coenenchymal covering. One specimen (SBMNH 422893) harbors a small brittle star, wrapped around a portion of the main trunk. No zooxanthellae present in the tissues, particularly true of USNM 50186; specimen examined for their presence by Bayer.

##### Remarks.

Among the eight to ten specimens that Bayer examined in 1958, there appeared to be three main areas that showed variation: thickness of branches, development (size) of the calyces (if present), and proportions of different sclerite forms (those with leafy sculpturing as compared to common spindles/capstans) (Bayer unpublished ms 7, Cairns 2009). Specimens at SBMNH do illustrate variation in branch diameter. Terminal branches range in diameter from 2.0–4.5 mm. Branches with smallest diameter have very distinct, prominent calyces, arising conically from their base, but thicker branches have less conspicuously prominent calyces, actually coenenchymal mounds appearing as low bumps; polyps fold into simple openings that appear as pores. One specimen at NMNH, USNM 50187, is of an extreme form; some of the branches are quite slim, bearing very pronounced calyces. It became clear that similar variations were not of taxonomic significance. Other more slender specimens exhibit a wide range of variation in branch diameter.

Most field/diving guides imply that this species is fairly contained within, and to, the region of La Jolla, California. Several of the SBMNH specimens argue against this; it appears that this particular species ranges a bit further south (upper/lower Baja) than had been previously reported. Three lots examined and confirmed correct as to their genus identification (at least) implied either: 1) a range that extends further south and/or 2) the presence of several other species, including *Adelogorgiatelones* Bayer, 1979, and one or more of the species recently described by Breedy and Guzmán (2018), in the collection. As to potential range of distribution, Bayer (1979) made the following comment in his description of *A.telones*: “Although it (*A.phyllosclera*) seem(ed) to be rather common in the vicinity of La Jolla, collections made farther to the south, in Baja California, by the same team of divers, (did) not include it (*A.phyllosclera*). Neither does it occur in other collections from Baja California and the Gulf of California taken by diving or dredging, nor in collections obtained by the US Fish Commission steamer ‘Albatross’ by dredging and trawling at many localities along the coast of Central America and South America”. There are, however, several specimens in the SBMNH collection taken from northern Baja California that clearly appeared to be this species (see Appendix 1: List of material examined). Further sightings/collections would help to confirm this species’ total range (where it may either transition to *A.telones*, or other recently discovered species, or has a definite southern limit, with *A.telones* and other species then appearing some distance further south). In the examination of specimens from California locations, and those from the Galápagos Islands, in the SBMNH collection, the distinctive differences that would separate species were not clear; all specimens, with one exception (completely bleached, SBMNH 422891 from Santa Cruz Island, Galápagos Islands), are the distinctive red color of this species. *A.telones*, by contrast, is typically either yellow or white (Bayer 1979, Breedy and Guzmán 2018). Bayer (1979) stated that, in a comparison of the two species, *A.phyllosclera* has: 1) branching that is more crooked and open, 2) polyps that form distinctly hemispherical or blunt-conical calyces (as opposed to calyces being inconspicuous or not really present at all), and 3) sclerites somewhat larger, with double wheels/discs somewhat different in shape along with being more elaborately sculptured. Based on the coloring of specimens (with one exception) in the SBMNH collection, along with Bayer’s (1958) discussion of variation in characters within this species, there are unanswered questions regarding distribution of this species and the potential for several other species, as represented in the SBMNH collection. Both *A.phyllosclera* and *A.telones* are accepted species in the WoRMS Data Base (Cordeiro et al. 2018a), along with three others.

#### Genus Eugorgia

Taxon classificationAnimaliaAlcyonaceaGorgoniidae

Verrill, 1868

Lophogorgia (pars) Horn, 1860: 233.
Gorgonia (pars) Verrill, 1864: 33; 1866: 327.
Leptogorgia Verrill, 1864: 32.
Eugorgia (pars) Verrill, 1868c: 414.
Eugorgia Verrill, 1868b: 406–407. Studer 1887: 64–65. Bielschowsky 1918: 39. Kükenthal 1924: 343. Bielschowsky 1929: 170. Stiasny 1951: 63. Bayer 1951: 99; 1981: 921. Breedy et al. 2009: 8.

##### Type species.

*Leptogorgiaampla* Verrill, 1864; by subsequent designation Verrill 1868b: 386.

##### Diagnosis.

Breedy et al. (2009) did a thorough review of this genus (well represented in the SBMNH collection). Sclerites chiefly disc spindles, capstans or double discs (wheels); double discs up to 0.05 mm long, spindles 0.12–0.18 mm, not developed as clubs; ordinary spindles present in small numbers in some species. Anthocodiae unarmed; sclerites, if present, flat rods and platelets with lobed margins. Polyps fully retractile into coenenchyme, slightly raised to prominent mounds, forming polyp-mounds, in longitudinal rows; often evenly distributed on all sides of branches. Branching is lateral, dichotomous (partial) or pinnate-like, in one plane; if bushy, branches in multiple planes; no anastomoses. Axis contains network of frequently mineralized organic filaments. Colony colors quite variable, depending on species.

#### Eugorgia daniana

Taxon classificationAnimaliaAlcyonaceaGorgoniidae

Verrill, 1868

**Figure 4.**
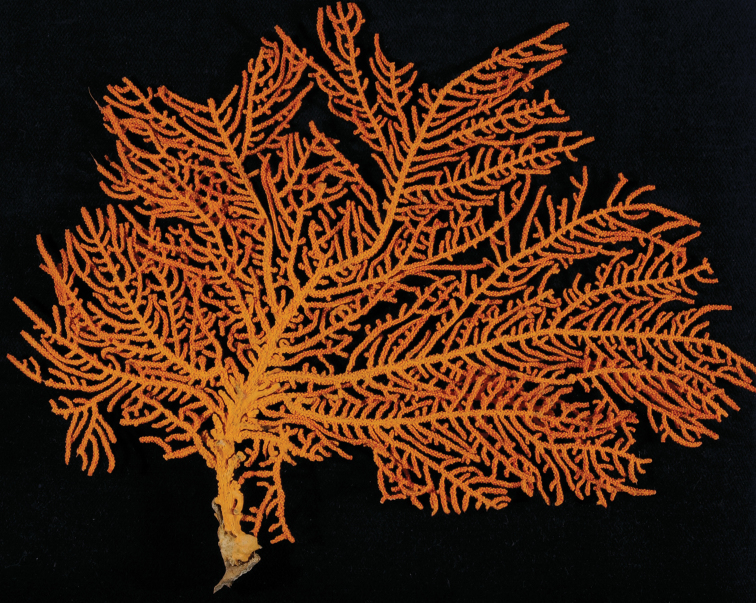
*Eugorgiadanianae*, SBMNH 422897. Shows complex branching that creates wide, flat fan. Colony is 24 cm high × 25.5 cm broad at widest point.

**Figure 5.**
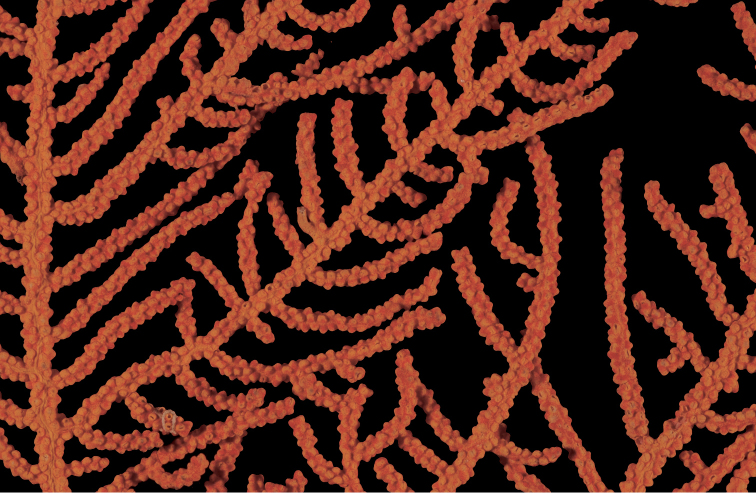
*Eugorgiadanianae*, SBMNH 422897. Close-up of pinnate branching pattern.

**Figure 6.**
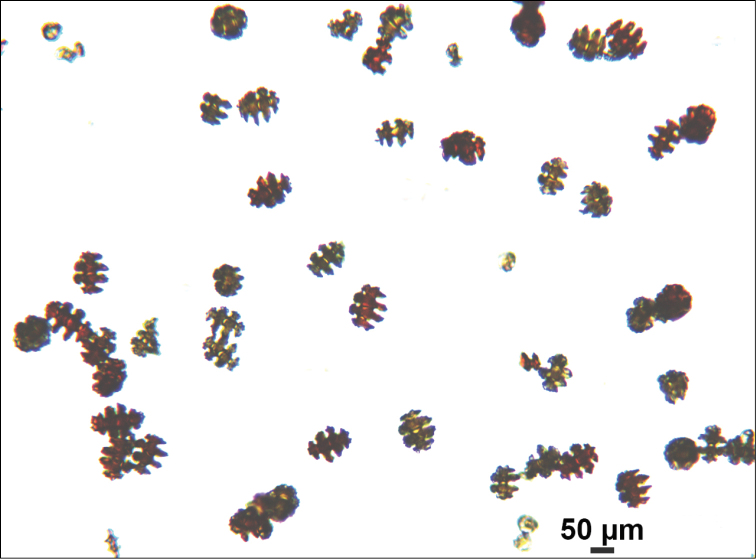
*Eugorgiadanianae*, SBMNH 422897, light microscopy array, 10× magnification. Note jagged, sharp-edged, double discs and color variety. Sclerites will appear orangey-yellow collectively but individuals can be deep, bright red, yellow-green, or bicolored, with a maximum length up to ~100 µm.

**Figure 7.**
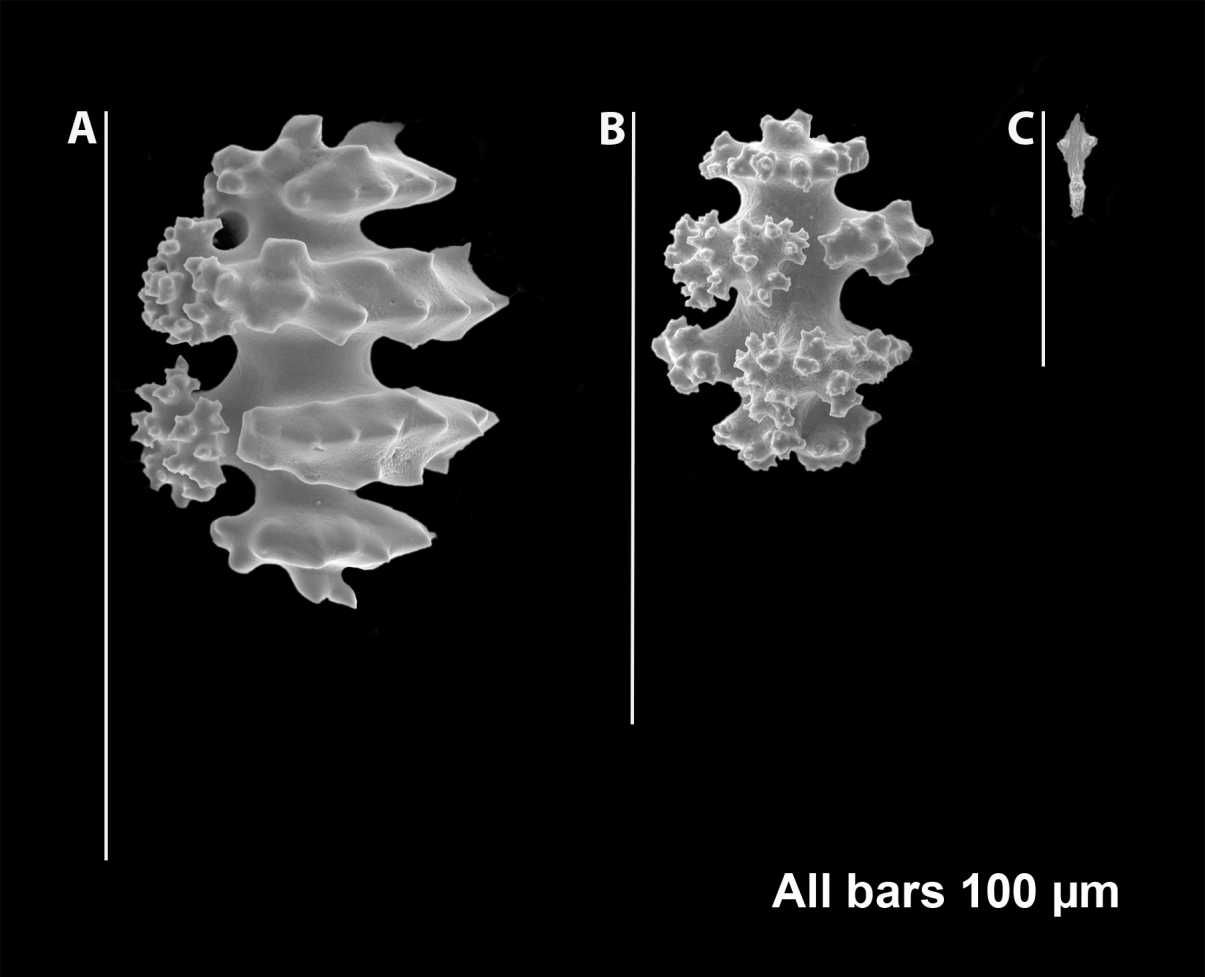
*Eugorgiadanianae*, SBMNH 422897, SEM image. Representative sclerite of each form. **A** Double-disc **B** Coenenchymal sclerite **C** Anthocodial sclerite. Images here compare well with those shown in Breedy et al. 2009 (fig. 8).

Figures 4 , 5 , 6 , 7

Eugorgia daniana Verrill, 1868a; 1868b [1869]: 409–410; pl V, fig. 14; pl VI, fig. 7. Bielschowsky 1918: 45. Kükenthal 1924: 346. Bielschowsky 1929: 181. Stiasny 1951: 65. Prahl et al. 1986: 17. Breedy et al. 2009: 17–20.

##### Type locality.

Central America: Pearl Islands; Costa Rica, Gulf of Nicoya.

##### Type specimens.

**Syntype series** (Breedy et al. 2009): MCZ 723 [dry]; MCZ 7080 [dry]; YPM 1551a-d and 1629a, b [dry]; YPM 5146 [dry].

##### Material examined.

~10 lots (see Appendix 1: List of material examined). Was unable to examine the type specimens, but utilized descriptions and images noted in Breedy et al. (2009).

##### Description.

Collection lot examined, shown in Figures 4 (whole colony), 5 (branch magnified to show prominent polyps), 6 and 7 (sclerites, light microscopy and SEM, respectively), generally matches description given in Breedy et al. (2009: 17–20, 35). Color of branches tended generally to dark orangey-red, with coenenchyme base of polyp-mounds red, upper portion of polyp-mounds gold-orange; overall impression is that colony is basically red. Sclerites (Figure 6) either bright to deep red or yellow-green in color, mixed together. In some instances an individual sclerite can be bicolored (red at one end, other end yellow-green); sclerites as double discs, relatively large; inner wheels thin, with sharp edges, outer ones terminal, not half as large, also sharp-edged (Figure 7A) (see remarks below).

##### Common name.

Red gorgonian (Gotshall 1998).

##### Distribution.

As recorded by Verrill (1868a and 1868b [1869]) found in Panama and the Pearl Islands, 11–15 m (this according to FH Bradley); also seen in the Gulf of Nicoya, Costa Rica (JA McNiel). Other notations indicated that it could extend down to Peru (see Breedy et al. 2009: table 4 for full, known distributional range). Based on one specimen, USNM, 57302, taken in Escondido Bay, near San Diego County, San Diego, California, the overall range would extend from San Diego, California (at least), through Central America, possibly down to South America. Assuming *E.daniana* is a distinct species, it is then present in Central and Lower Gulf of California, living in the same area with *Eugorgiaaurantiaca* Horn, 1860. Thus, southern-most end of the California Bight may be the northern-most limit (and as a separate species, *E.daniana* extends just a bit further north and further south than *E.aurantiaca*).

##### Biology.

Generally found on offshore reefs and islands; prefers clean, plankton-rich waters and generally found at depths to ~30 m (Gotshall 1998).

##### Remarks.

While description generally matches that given in Breedy et al. (2009), I would make the following caveats: first, the colony color as described by Verrill was as a bright yellow, streaked and blotched with dark red on both branches and polyps. The color seen in specimens here lies somewhat intermediate to that described in Olvera et al. (2018) for *E.aurantiaca* and *E.daniana*; overall, dark orange-red. Divers have anecdotally described the living colony as having slender red branches, with white (colorless) polyps.

For Verrill (1868b [1869]: 411), “In the mode of branching, the size and structure of the branchlets, and color (*E.aurantiaca*) closely resembles (*E.daniana*),” which he separated primarily on the basis of the very different size and form of the sclerites, especially of the double discs; for Verrill, these were the defining feature in designating this species as distinct from *E.aurantiaca*. In my examinations, the larger, “sharp” double discs were always present (distinctive of *E.daniana*). However, in a few specimens used for comparison, labeled as *E.aurantiaca*, the presence of sharper double discs was noted (misidentified specimens?); this condition was contrary to the generally slightly smaller size of the double discs that are commonly seen in *E.aurantiaca*, where discs are described as being generally rounded, inner and outer discs very close together. In subsequent comparisons, the polyps were not always so densely packed in *E.daniana* as was seen in *E.aurantiaca* (no overlapping of polyps). With regards to branching pattern, specimens of *E.aurantiaca* often had a far more distinctive (and decidedly symmetrical) pattern of pinnate branching, with majority of secondary branches (branchlets) of similar length, all generally lying in one plane as compared to that seen in *E.daniana*. SBMNH specimens of *E.daniana* nearly always had their terminal-most, thin, slender branchlets curving out of one plane. Without molecular investigation, there is no clear, definitive confirmation that *Eugorgiaaurantiaca* and *Eugorgiadaniana* are indeed separate species. There are many overlapping features, in terms of potential branch pattern, form, and size of sclerites, and general color. The differences could be accounted for as variation within one species. However, examination of numerous examples of both those identified as *E.aurantiaca* and *E.daniana* allowed for observation of the differences in the double discs that Verrill used to distinguish these two species (differences can be obvious). For now, it seems appropriate to recognize two separate species until further studies prove otherwise.

Of note is that WoRMS Data Base (Cordeiro et al. 2018b) does list *E.aurantiaca* and *E.daniana* as separate species, but that *E.daniana* has been accepted as *Leptogorgiadaniana*. Very few species of *Leptogorgia* (*L.ramulus* Milne Edwards & Haime, 1857, is one of few) display the shorter branch lengths seen consistently in species of *Eugorgia* nor the irregular and pinnate branching of the *Eugorgia*, which are morphological characters; it is understood that this acceptance is based on the molecular work that was done by Soler-Hurtado et al. (2017). Of note is the rationale for this genus change, with Soler-Hurtado et al. (2017) noting the 1999 ICZN Principle of Priority (Article 23). In the work of Olvera et al. (2018), this species is listed as a species of *Eugorgia* and is not included in the list of *Leptogorgia* species that are discussed.

#### Eugorgia rubens

Taxon classificationAnimaliaAlcyonaceaGorgoniidae

Verrill, 1868

**Figure 8.**
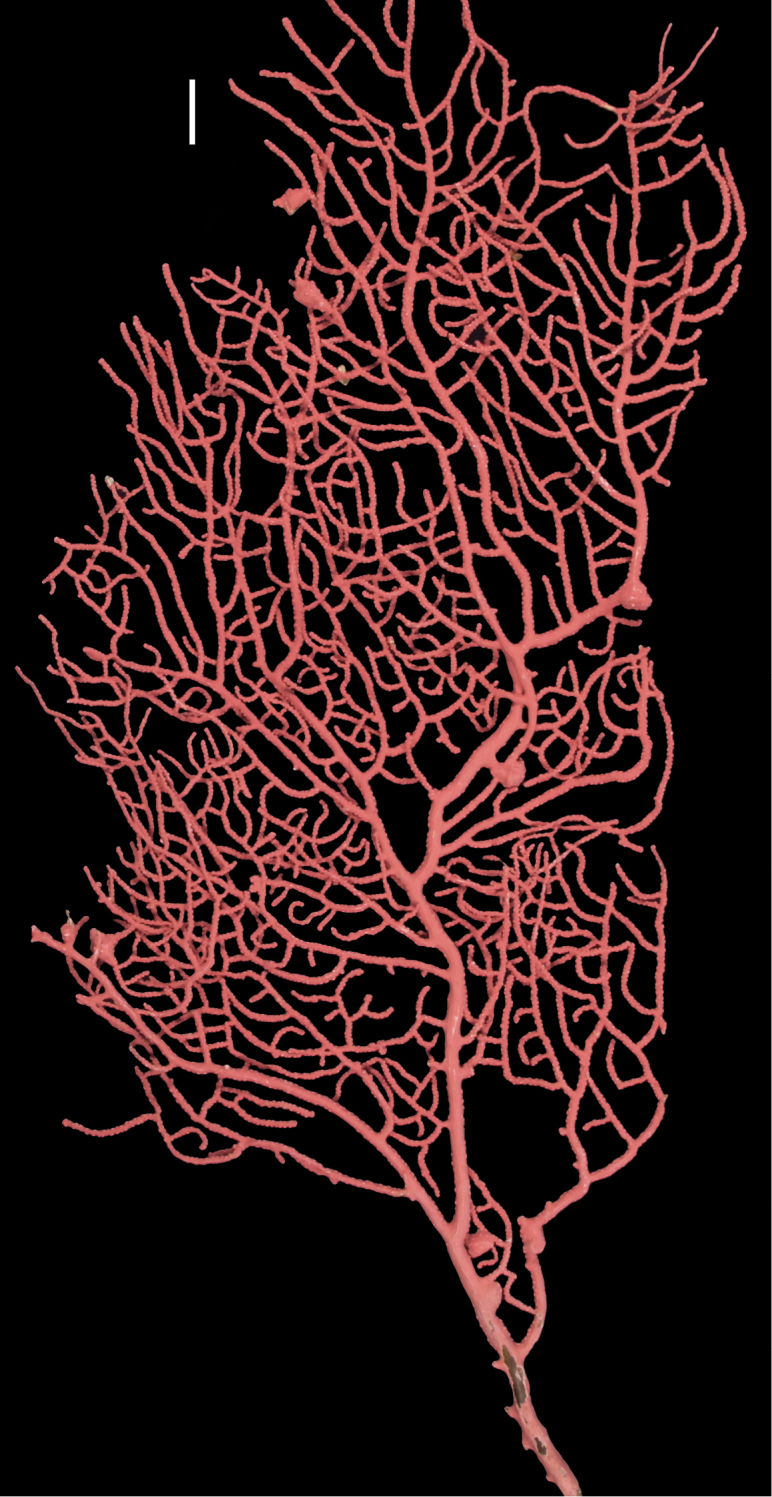
*Eugorgiarubens*, SBMNH 422900. Colony 45 cm tall, 18 cm across (through broadest, middle section). Scale bar: 2 cm.

**Figure 9.**
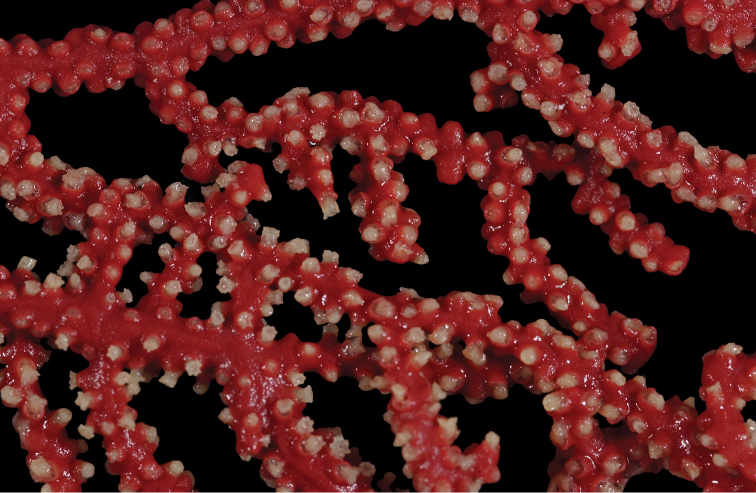
*Eugorgiarubens*, SBMNH 45562. Magnified, showing branching and extended polyps.

**Figure 10.**
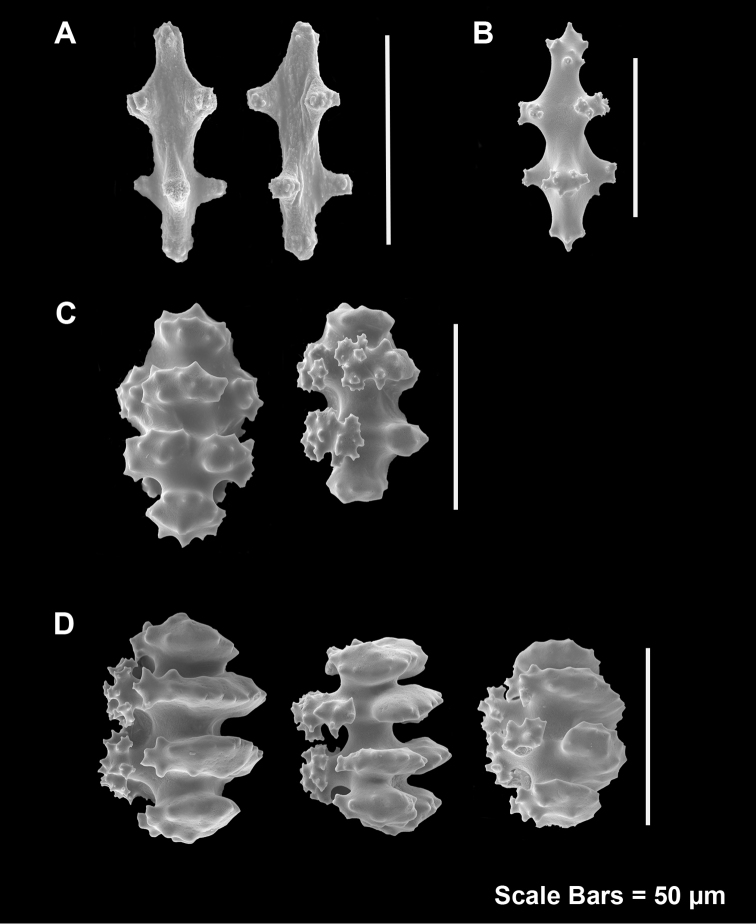
*Eugorgiarubens*, SBMNH 45562, SEM image. Color of sclerites purple. Representative sclerite of each form. **A** Anthocodial sclerites **B** Anthocodial sclerite or small sclerite of coenenchyme **C** Coenenchymal spindles **D** Double disc spindles. Images here compare well with those shown in Breedy, et al. 2009 (fig. 15).

Figures 8 , 9 , 10A–D

Eugorgia rubens Verrill, 1868b [1869]: 411 [not figured]. Studer 1894: 69. Bielschowsky 1918: 45. Kükenthal 1924: 346. Bielschowsky 1929: 183. Breedy et al. 2009: 29–31.

##### Type locality.

SE Pacific Ocean, South America, Peru, Piura Dept., Paita. The type locality is often incorrectly spelled as Payta.

##### Type specimens.

**Type**–YPM 1779 [dry]; MCZ 36047 (slide of **holotype**).

##### Material examined.

~ 50 lots (see Appendix 1: List of material examined). Was unable to examine the type specimen, but examined many others at NMNH, CAS, etc.; this is easily recognizable and comparison to type was not necessary.

##### Description.

In general, all SBMNH material (along with supplemental lots) examined falls in line with the description and images provided for this species by Breedy et al. (2009: 5–7, 29, 31, 42). Whole colony (Figure 8), branch pattern (Figure 9), and basic sclerite forms (Figure 10) demonstrate the identifiable characteristics for the species.

##### Etymology.

*Rube-* is the Latin for red, or reddish, presumably in reference to this species’ color. However, there is no explanation for species name given by Verrill.

##### Common name.

Purple gorgonian (Gotshall 1994); Purple sea fan; Reddish true gorgon. Common name not specified in Cairns et al. (1991; 2003).

##### Distribution.

From southeastern Pacific Ocean (type locality: Peru [Paita]), to southern and central California (Santa Barbara mainland and Channel Islands). Depth range from shallow subtidal to deeper than 100 meters. An extensive number of specimens were examined; collection location data shows geographic and depth ranges.

##### Biology.

Found at depths usually greater than 10–30 m. Work by Lissner and Dorsey (1986) showed a range of depth for this species along Tanner and Cortes Banks and the Santa Rosa-Cortes Ridge off of southern California as follows: At depths < 49 meters, the species was sparse, at depths ranging from 49–79 meters, the species was very abundant to abundant, from 79–91 meters the species was commonly seen, and at depths below 91 meters was again sparse. In e-mail correspondence with C Bauder (and subsequently T Laidig at NOAA, December 2010), this species may actually occur at depths greater than originally thought, extending much further north than previously reported (the specimen that called attention to this greater depth was photographed by C Bauder at Point Lobos, Carmel Bay, at 66.5 m). A thorough examination of specimens taken from these greater depths at this, and other more northerly locations, should be done. From a list found in Museum records, depth ranges for this species from selected California sites (south to north) are as follows: **Mainland**: Tijuana River: 36 m; Point Loma: 21–42 m; La Jolla: 20–64 m; San Pedro: 17–33 m; Carpinteria: 36 m; **Islands and Seamounts**: Coronados Islands: 39 m; San Clemente Island: 9–20 m; also common around the San Benito Islands off Baja, California.

Several dry specimens examined showed the presence of distinct galls produced by a species of acorn barnacle, projecting out through the coenenchyme. One of the wet specimens examined had a pronounced mass of red algae, with sponge, hydroids, worm tubes, etc. On another wet specimen, white scaly-looking patches were present, one patch so dense it looked like a cushion. On both of these wet specimens, the masses of growth were generally only present on areas of the colony where the axis was fully exposed. There is also evidence of the presence of ovulid snails from the Genus Simnia (Neosimnia) Risso, 1826 (species *S.barbarensis* Dall, 1892 and *S.loebbekiana* Weinkauff, 1881) as well as *Simnialenarufa* (Sowerby III, 1832) in the branches of both California (Santa Barbara, East Beach, Slate Reefs, 24–27 m; 1 April 1967 and off Newport Beach, 6 m; 18 Dec. 1964) and Mexican-collected specimens (from Sonora, Guaymas, Miramar Cove, 0.9–2 m; October, 1965; however, latter of questionable species identification).

##### Remarks.

Type specimen donated to YPM by FH Bradley who originally received it from Mrs George Petrie. Not recorded in early monographs on the alcyonarian fauna of California (Nutting 1909; Kükenthal 1913). While I was unable to examine the type specimen, all specimens in the SBMNH research collection are readily identifiable as this species. In truth, many of the specimens in the SBMNH collection are excellent examples of this species; examination of the type was not necessary.

NMNH has several catalogued lots in their collection with location records from the Santa Barbara Channel and the California Channel Islands (many of these identified to genus only); also several lots identified to species, collected in the area of La Jolla: Scripps Canyon, La Jolla Canyon, as well as the southern part of California and Del Mar. In addition, NMNH has several lots collected by C Limbaugh. Several of these are from the La Jolla area, but as well, from a couple of locations not previously recorded, including the Richfield Oil Island, Redondo Beach, and Rocky Point, in close proximity to Point Vicente, at the south end of Santa Monica Bay. In addition, the Cabrillo Marine Aquarium, in San Pedro, California has a few dry specimens of this species in its museum, and as well, displayed live specimens in tanks on exhibit to the public; generally, all were collected from the local area. This is a very common species in Southern California waters and is an accepted species in the WoRMS Data Base (Cordeiro et al. 2018b).

#### Eugorgia ljubenkovia sp. nov.

Taxon classificationAnimaliaAlcyonaceaGorgoniidae

http://zoobank.org/48F9BA66-D012-44A5-8C8F-1EEAE2279548

**Figure 11.**
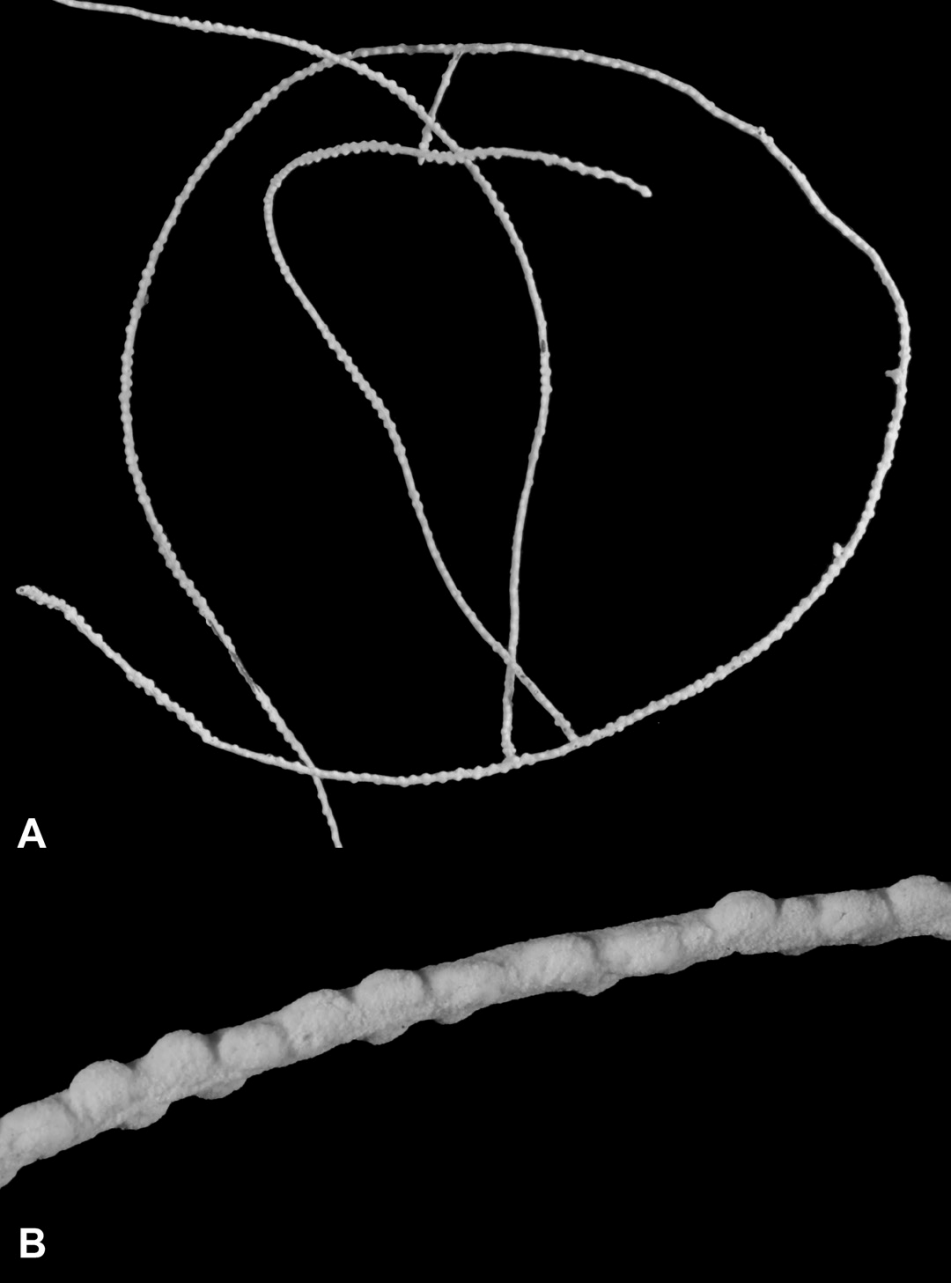
*Eugorgialjubenkovia* sp. nov., SBMNH 472232. **A** View of long, coiled specimen strands; when fully extended, longest strand length ~37 cm. Strand diameter (excluding polyp mound) ~1.0 mm **B** Magnification of branch strand, showing small, rounded polyp mounds.

**Figure 12.**
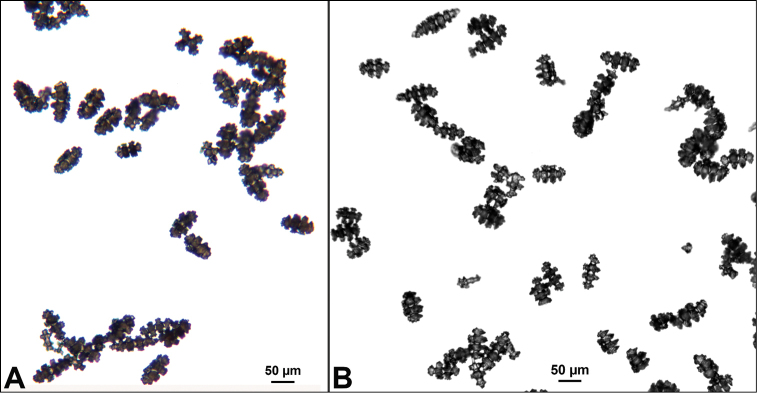
*Eugorgialjubenkovia* sp. nov., SBMNH 472232, light microscopy sclerite array. **A** 10×, showing rounded spindles and double discs. Note apparent quadriradiate, upper right **B** Sclerite array at 10×, illustrating stout, sharp-toothed double discs (seen in some species of *Eugorgia*). Sclerite color is white, but may give colony a very, very pale pink cast when in the tissue. Largest sclerites up to 100 µm, those shorter, rounder up to 70–75 µm.

**Figure 13.**
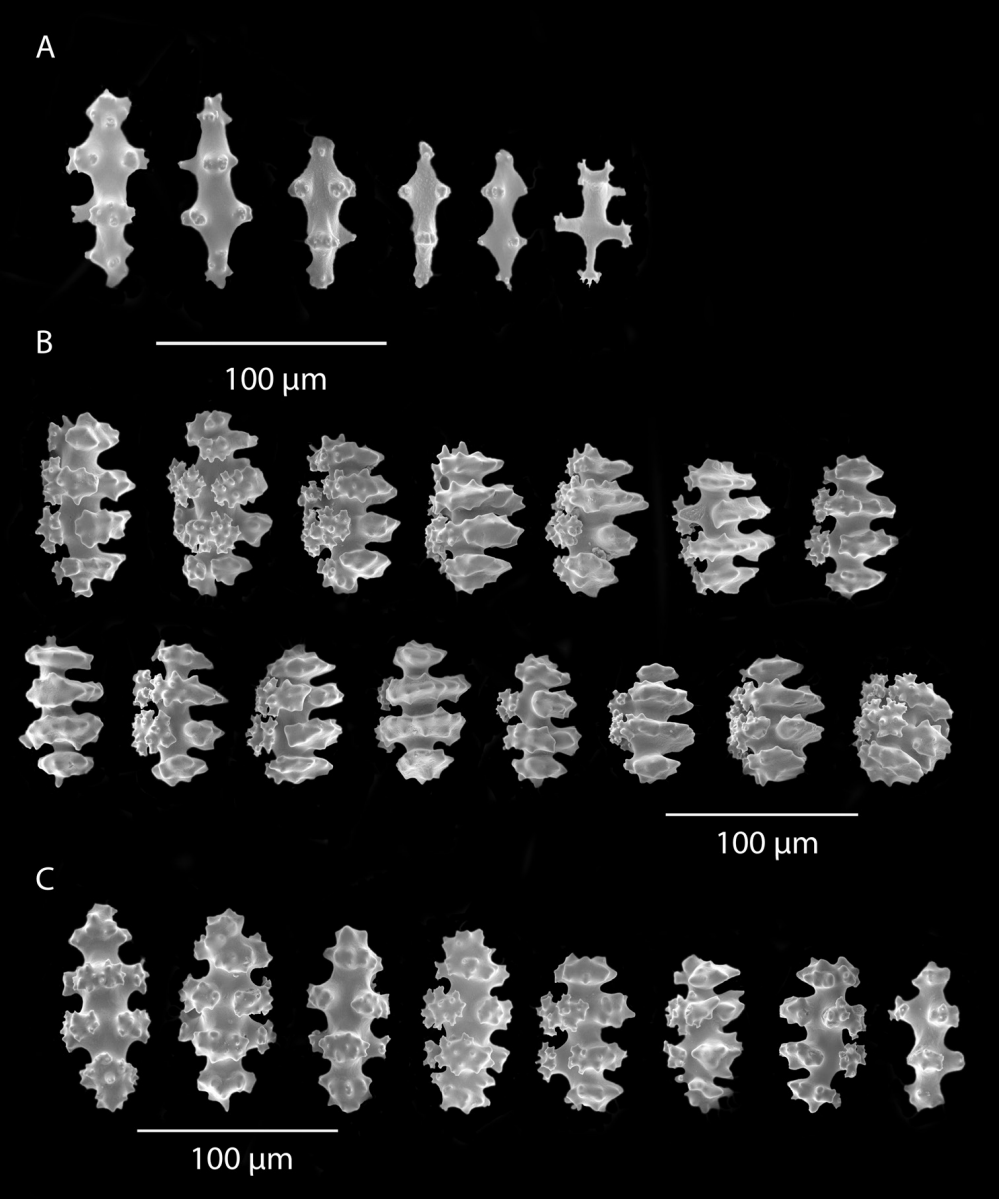
*Eugorgialjubenkovia* sp. nov., SBMNH 472232, SEM image. Sclerite color is white, but may give colony a very, very pale pink cast when in tissue. **A** Anthocodial sclerites **B** Double-discs (teeth evident in most) **C** Coenenchymal spindles.

Figures 11A, B , 12A, B , 13A–C

##### Type locality.

Isla Cedros, Baja, Mexico.

##### Type specimens.

**Holotype** Santa Barbara Museum of Natural History, SBMNH 422333.

##### Material examined.

~5 lots (see Appendix 1: List of material examined).

##### Diagnosis.

Colony an obvious whip-like form, no apparent holdfast, with minimal to no branching (not common to genus), branches fairly slender, with both branch ends pointed; sclerites double-disc spindles, with disc edges quite angular and sharp, characteristic of genus.

##### Description.

*Colonies* (Figure 11A) incomplete, with exception of one (total strand length of complete one, 58.5 cm; length of largest colony fragment, ~37 cm); diameter 1.0 mm (largest diameter up to 3.0 mm, when polyps included); few with tiny holdfast; long, thin, stiff, wiry strands, none or very few primary or secondary branches (unusual for species in this genus); what branches are present come off at right angles to main stem, then curve some five cm distant or more from branching point; branch pattern (one colony) more dichotomous or lateral. Tips of branches (both ends) terminate in small arrowhead configuration. Coenenchyme very thin; axis visible through it in some areas; color of colonies generally pure white, light creamy beige to very, very pale pink, both coenenchyme and polyps; axis red-gold, with greenish cast. Small polyp mounds (no more than 1.0 mm tall, 1.0–1.5 mm broad at base) moderately prominent, rounded, conically broad bumps arranged in nearly opposite (occasionally alternate) pattern, lateral, giving strands flattened appearance from front to back (closer examination revealed polyps on all sides); very thin ridge line (longitudinal ridge) runs down middle of both “front” and “back” of branch; appears as bare “thread,” slightly raised; overall, polyps give branches a distinct zig-zag edge on lateral sides. Polyps (Figure 11B) sit very close together, bases touching; greatest distance between polyps ~1.0 mm. Polyp aperture oval-shaped to thin slit, aligned with long axis of branch. Sclerites (Figures 12, 13) small; predominant type are double disc spindles (Figure 13B), with disc edges quite angular and sharp (common in species of *Eugorgia)*; also, slightly longer, symmetrical spindles (more typical of species in genus *Leptogorgia*, Figure 13C) and occasionally, crosses (quadriradiates); all are colorless.

##### Etymology.

Proposing *Eugorgialjubenkovia*, to honor John Ljubenkov, a southern California cnidarian biologist, colleague and friend of many Southern California Association of Marine Invertebrate Taxonomists (SCAMIT) members.

##### Common name.

John’s wire gorgonian.

##### Distribution.

Known from collection events undertaken by staff of Orange County Sanitation District and one lot taken in South Bay, Isla Cedros (‘Velero IV’) in 1949; thus, at this time, known from southern California and northernmost Baja, Mexico.

##### Biology.

Moderate occurrence, indicated by OCSD collection events; not occurring at great depth (~30–35 m). Hydroids (fuzzy mass) attached to bare axis on one colony (SBMNH 472233); elsewhere on same colony, barnacle galls, barnacles completely covered with gorgonian’s coenenchyme.

##### Remarks.

All colonies have shape of a thin, whip-like *Leptogorgia* species, and may exhibit the same presumed lifestyle as that of *Leptogorgiafilicrispa* Horvath, 2011 or that of species in the genus *Thesea* Duchassaing & Michelotti, 1860. However, sclerites most distinctive in being largely double discs, characteristic of species in the genus *Eugorgia*. Originally, was tentatively identified as a possible *Heterogorgia* Verrill, 1868c by J Ljubenkov; these specimens did not show the characteristic collaret, point and thorn sclerites of that genus (and that genus does not display distinct double discs, as seen here). Originally, SBMNH 422333 was shelved with specimens of the genus *Thesea*; despite the long, thinner branch strands and possible lifestyle similarity, no large spheroidal bodies, characteristic of *Thesea* were found. Also, this species (in five lots), is not additional material of the species *L.filicrispa* (Horvath, 2011), as jagged double discs are not seen in that species, and sclerites in that species can be variably colored. This species is a unique mix of colony form seen in some *Leptogorgia* with the sclerites of a *Eugorgia*. The long, thin wiry condition of the stems may be the result of environmental circumstances, involving both substrate (sandy or soft bottom sediment) and water flow. From examination of all specimens, it seems possible that some strands have no attachment base, but instead have terminal tips at both ends of strand. Further in situ work would need to be undertaken to document the environmental conditions under which this species lives.

#### Genus Leptogorgia

Taxon classificationAnimaliaAlcyonaceaGorgoniidae

Milne Edwards & Haime, 1857

Gorgonia (pars) Pallas, 1766: 160. Milne Edwards and Haime 1857 (pars): 157.
Leptogorgia Milne Edwards & Haime, 1857: 163. Verrill 1864 (pars): 31, 33; 1868b: 387; 1869b: 420. Studer 1887 (in Wright): 64. Bielschowsky 1918: 18. Kükenthal 1919: 851; 1924: 324–325. Bielschowsky 1929: 81. Stiasny 1943a: 87. Bayer 1951: 98–99; 1956a: F212; 1961: 214. Grasshoff 1988: 97; 1992: 54. Williams 1992: 231. Williams and Lindo 1997: 500. Bayer 2000: 609. Breedy and Guzmán 2005: 2; 2007: 6. Horvath 2011: 46. Breedy and Cortés 2011: 63.
Lophogorgia Milne Edwards & Haime, 1857: 167. Nutting 1910c: 3, 4. Kükenthal 1924: 322. Bielschowsky 1929: 73. Stiasny 1943: 87. Bayer 1951: 99; 1956a: F212; 1961: 194. (Type species by monotypy: Gorgoniapalma Pallas, 1766 [South Africa]).

##### Type species.

*Gorgoniaviminalis* Pallas, 1766; subsequent designation by Verrill 1868a.

##### Diagnosis.

Sclerites primarily symmetrical spindles, most without unilateral fusion of warts to form discs; shorter ones may have warts on one side fused like those of disc spindles; long ones symmetrical or with warts on one side simple, conical, elsewhere complex tubercles in various arrangements (several whorls). Coenenchyme generally contains only spindles and radiate capstans with symmetrically developed tuberculation; warts/tubercles mostly in two whorls on capstans. Anthocodial armature flattened rods; sometimes, ovoid platelets. Colonies little-branched, long, slender, whip-like, or short with branching variable: pinnate, lateral or dichotomous, in one plane or bushy; color of colonies highly variable. Colonies either attached to substrate with holdfast or lying free on substrate. Axis consistent for family, containing network of organic filaments, frequently mineralized. Polyps fully retractile into coenenchyme; slightly raised, mound-like, around apertures.

##### Remarks.

Bayer (1951) stated that *Leptogorgia* contains many species in temperate and tropical waters; although represented practically around the world, the center of distribution seems to be the west coast of Central America (Breedy and Guzmán 2007, Breedy and Cortés 2011, Breedy and Guzmán 2012).

#### Leptogorgia chilensis

Taxon classificationAnimaliaAlcyonaceaGorgoniidae

(Verrill, 1868)

**Figure 14.**
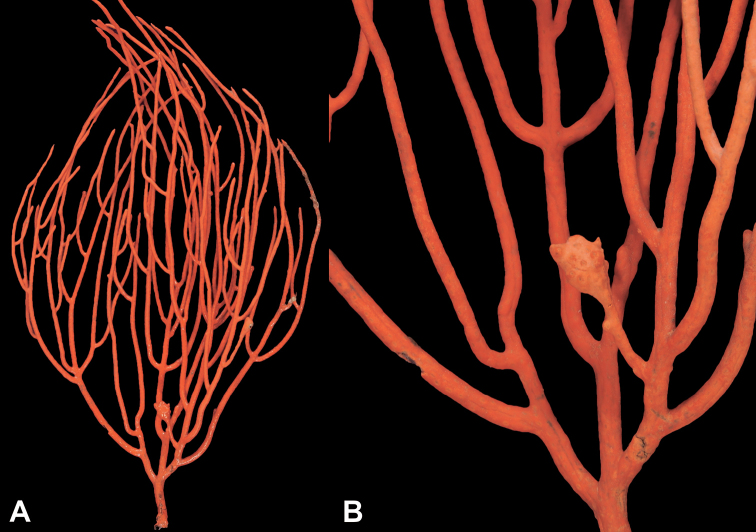
*Leptogorgiachilensis*, SBMNH 422953. **A** Colony measures 25 cm, maximum length, 13.5 cm, broadest width **B** Detail of branching pattern (branch diameter ≤ 2.0 mm), and gall formation created by species of barnacle, a common occurrence on this and other gorgonian species.

**Figure 15.**
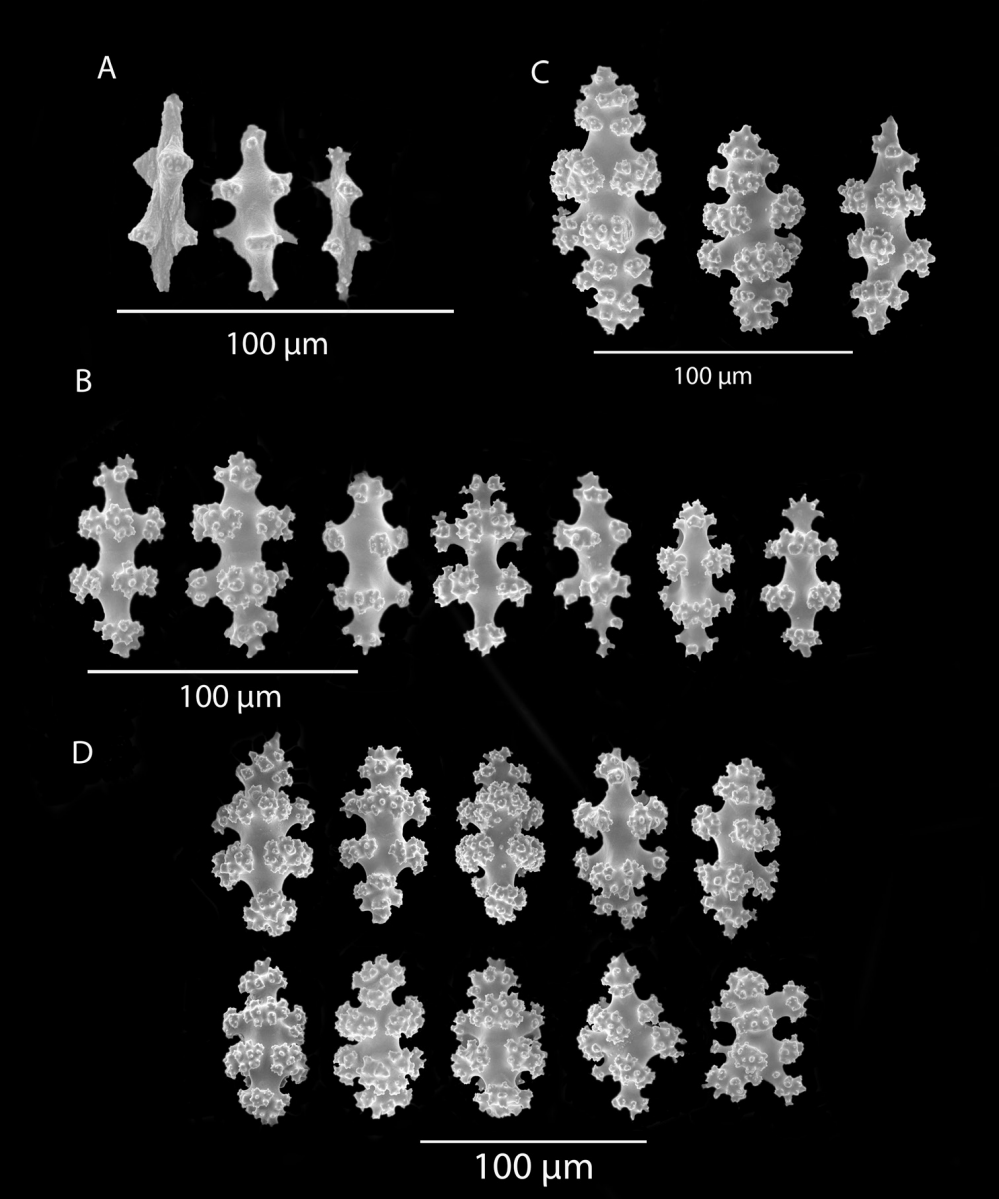
*Leptogorgiachilensis*, SBMNH 422953, SEM image. Color of sclerites is orange-red. **A** Anthocodial sclerites **B–D** Spindles of coenenchyme. Images closely match those shown in Breedy and Guzmán 2007 (fig. 14).

Figures 14A, B , 15A–D

Plexaura rosea Philippi, 1866: 118 (junior homonym, Breedy and Guzmán 2007).
Leptogorgia rosea Phillipi, 1892: 7 (as: Verrill 1868b: 406 (nec Leptogorgiarosea Milne Edwards & Haime, 1857: 134).
(?) Litigorgia (?) rosea: Verrill 1868a; 1868b: 406. Philippi 1892: 7.
Nec Litigorgiaflexilis Verrill, 1868a.
Leptogorgia (?) chilensis Verrill, 1868b: 406.
Leptogorgia chilensis Kükenthal, 1919: 772; 1924: 355. Bielschowsky 1929: 132. Breedy and Guzmán 2007: 22–25.

##### Type locality.

Apparently, originally collected from Chile, south of Valparaiso, and off Algarrobo. For **neotype** (designated here), northeastern Pacific Ocean, North America, USA, California, Santa Barbara County, Goleta, Sands Beach, ~6 m; coll. R/V ‘Vantuna’ Cruise #469, November 2001.

##### Type specimens.

Location of original type specimen not known. **Neotype** (here designated) SBMNH 422953 [wet].

##### Material examined.

~25 lots (see Appendix 1: List of material examined).

##### Description.

*Colony* (Figure 14A) not reticulate; bushy, often lanky; branches spread out, in loosely subpinnate or dichotomous, irregular branching (Figure 14B) pattern; color of living colony orange-red to orangey salmon-pink. Limbaugh (unpublished key) described color as a rich salmon pink; polyps white; dry specimens pale orange to light salmon pink. Branches and branchlets very cylindrical, long, often greater than 30 mm in length, slender (2.0 mm), usually smooth and whip-like, with unbranched, pointed ends. Branches/branchlets lie roughly in one plane (not always); some branching in all directions. Colony height to 3 ft (~92 cm); usually 2 ft (~61 cm) or less. Polyps generally flush in complete retraction, forming oblong apertures, extending in all directions around the branches. Generally, several longitudinal grooves (in bare area between polyps) present. Tentacles on polyps taper at tips and bear two rows of lateral pinnules, slightly displaced to the oral side. Sclerites (Figure 15A–D) commonly spindles having acute or subacute warted ends extending beyond second ring of warts on either side of median girdle; also capstans (two whorls with end tufts), modified as disk-spindles. Anthocodial sclerites small rods, thin, sparsely ornamented; sclerites generally orange in color. What is shown here (Figure 15) comparable to that shown in Breedy and Guzmán (2007: Figure 14, page 24).

##### Etymology.

*Lepto-* is Greek for fine or slender; the root *chilensis-*, likely indicative of the original type locality. No discussion of the derivation of the species name was found.

##### Common names.

Pink sea whip; Pink gorgonian; Red gorgonian; Common red sea whip; Chilean crested gorgon; Carmine sea spray; Violet sea spray (from a variety of field/diving guides, conversations with local divers, etc.).

##### Distribution.

Several general guidebooks, including that by Gotshall and Laurent (1979), state distribution as Monterey Bay to San Benitos Islands in Baja, California. Cairns et al. (1991, 2003) did not list this species. Specimens were collected locally (Santa Barbara area, 9–22 m) for studies done by Satterlie and Case (1978, 1979) on the neurobiology of gorgonian coelenterates. NMNH has numerous lots collected from La Jolla Canyon (USNM 50179), Scripps Canyon (USNM 50191), and southern California (USNM 52442). However, examination and comparison of sclerites taken from many “red whip” forms indicated that likely range of *Leptogorgiachilensis* is from Anacapa Island off the California coast (thus, from the middle of the California Bight), south, perhaps to the coast of Chile. Further discussion regarding distribution of this species can be found in the “Remarks” section of this description. That discussion may further clarify some of the confusion regarding this species of “red whip” amongst several others. Other “red whips” that extend from the middle of the California Bight northward, and overlap with *L.chilensis* in the extreme southern end of their range, may well be one or more different species (see “Discussion concerning diversity of “red whip” gorgonian forms,” following description of *Chromoplexauramarki*.)

##### Biology.

Gotshall and Laurent (1979) mentioned that this species likes offshore pinnacles, depths of 50 to ~200 feet (15–61 m). Another guidebook (Snyderman 1987) stated that: “Reds are very common on the Channel Islands and on offshore pinnacles as far north as Monterey.” “Reds” would certainly include this species, but the term “reds” is not exclusively a reference to this species. Found at depths greater than 60 ft (18 m); at Catalina, 40 ft (12 m). Range given elsewhere as 15–60 m deep. Lissner and Dorsey (1986) recorded a maximum depth of 77 m for this species on Tanner and Cortes Banks, off southern California. From a list for California sites, both mainland and islands, with depth ranges indicated, we see: **Mainland**: Tijuana River: 36 m; Point Loma: 18–42 m; La Jolla: 17–64 m; San Pedro: 12–33 m; Redondo Beach: 12 m; Santa Barbara: 9 m; **Islands**: Rock Pile (Seamount 8 miles S. Coronados Islands): 30 m; Coronados Islands: 15–39 m; San Clemente Island: 5–21 m; Santa Catalina Island: 8–26 m; Anacapa Island: 6–9 m; Santa Cruz Island: 2–6 m; Santa Rosa Island: 5–8 m.

This species has been studied both electrophysiologically and morphologically by Satterlie and Case (1978, 1979), and has been the subject of several studies regarding its (and other gorgonian species) relationships with other organisms, such as the obligate commensal barnacle *Conopeagaleata* (Linnaeus, 1771), formerly *Balanusgaleatus* Linnaeus, 1771(Gomez 1973, Lewis 1978, Standing et al. 1984, Crisp 1990, Langstroth and Langstroth 2000). I have seen in multiple instances that these barnacles cluster as galls, attached to the axial skeleton of this species and are overgrown by the gorgonian’s soft outer tissue. *Balanusnubilis* Darwin, 1854 is recorded as having been seen on the axial skeleton of dead “*Lophogorgia*” (*Leptogorgiachilensis*) in Monterey Bay (Langstroth and Langstroth 2000) (questionable gorgonian species identification); this may be opportunistic as it populates widely different sites in addition to this species. As well, several mollusk species have been recorded in association with this species, such as *Tritoniafestiva* Stearns, 1873, as reported by Gomez (1973) and several snails of the genus *Neosimnia* (now *Simnia*), such as *Neosimniabarbarensis* Dall, 1892 (Theodor 1967, as referenced in Langstroth and Langstroth 2000). An unidentified field guide indicated that the ovulid snail *Delonovolva* Sowerby III, 1881 lives and feeds on the branches of this gorgonian. Still other organisms may be seen associated with this species, such as other species of cnidarian; “red” gorgonian is often colonized by the zoanthid anemone, *Parazoanthuslucificum*, now *Savalialucifica* (Cutress & Pequenat, 1960), and likely other species, ultimately resulting in the death of all or most of the red gorgonian polyps (Patton 1972, as referenced in Langstroth and Langstroth 2000). In the SBMNH collection, data for several wet specimens on the Zoanthinaria shelves indicated this gorgonian as the substrate. A specimen, SBMNH 45570, collected from Avalon area of Santa Catalina Island, has *Epizoanthusinduratum* Cutress & Pequenat, 1960 attached, while SBMNH 45549M, collected from the NE end of Anacapa Island, has *Epizoanthusleptoderma* Cutress & Pequenat, 1960 attached to it and SBMNH 45550, collected from the Pinnacle off the quarry, near Avalon on Catalina Island, has *Savalialucifica* (Cutress & Pequenat, 1960) attached to it. As well, a specimen of *L.chilensis* (SBMNH 265962), recently collected by Scott Clark in 2010, on Platform A as part of a survey for Milton Love, has approximately one third of its branches festooned with a creamy yellow zoanthid. There is some specific substratum choice indicated here, and is apparently common among colonial zoanthids. On SBMNH 422944 (see Appendix 1: List of material examined–Part II), there are large clumps of hydroid, but only on bare axis portions of the branches in the colony.

According to Langstroth and Langstroth (2000), other organisms may be found associated with this species (although there is a question as to species identification of the gorgonian they discussed, as the examples are all from Carmel and Monterey Bays, in northern California; I suspect they may actually be looking at organisms on *Chromoplexauramarki*). They mention the bryozoan *Celleporinarobertsonae*, now recognized as *Costaziarobertsonae* (Canu & Bassler, 1923), the Broken-back shrimp *Heptacarpusflexus* (Rathbun, 1902), which may scavenge on sclerites, mucus and even toxic tissues from the surface of the gorgonian, a caprellid amphipod, specifically a skeleton shrimp, *Metacaprellaanomala* (Mayer, 1903), whose color may derive from their acquiring the pigment ingested while scavenging the gorgonian’s sloughed off debris, and the very small hermit crab *Parapagurodeshartae* McLaughlin & Jensen, 1996 (now recognized as *Pagurushartae* (McLaughlin & Jensen, 1966), as noted in McLaughlin and Asakura 2004), recorded as being found only at depths of several hundred meters (presumably on this species; identification of host gorgonian may be incorrect), in southern California.

As described by Fenical et al. (1981), work was undertaken to extract what has been described as a neuromuscular toxin, lophotoxin, from several species of Lophogorgia (Leptogorgia); *L.chilensis* has subsequently been found to produce this chemical, as well (Fenical et al. 1981). “Therefore, the distribution of toxin-producing gorgonians extends from Panama Bay northward to Point Conception, California” (Fenical et al. 1981). Also, it appears that gorgonians are able to distinguish (chemically?) between self colonies and not-self colonies (Langstroth and Langstroth 2000, citing Theodor 1970).

##### Remarks.

Harden (1979?) stated this as being one of the most common sea whips from southern California; my examinations confirm this. However, the fact that this is such a common species in southern California has led many to assume that all “red whip” forms (or those red and moderately branched forms), are this species, to the exclusion of others. The reality is that there are other red whip species which can easily be mistaken for *L.chilensis*; a cursory look by eye alone can (and has led) to misidentification.

Characteristics ordinarily used for separating *Lophogorgia* from *Leptogorgia*, the flattened branches and arrangement of zooids all around the branches and branchlets, are so variable as to be useless for generic distinctions (Bayer 1951). Round as well as flattened branches may occur in the same colony, and biserial zooid distribution can be found with little difficulty. Furthermore, specimens of *Leptogorgia* (typical in all other respects) may have zooids distributed all around the branchlets. Bayer first placed the genus *Lophogorgia* in synonymy with *Leptogorgia* in 1951; based on the work done by Grasshoff (1988), Bayer (2000) was then able to support that synonymy. Bayer used a specimen from Santa Catalina Island, collected at approximately 15 m to conclude that: “*Leptogorgiachilensis* (= *Lophogorgia*, as labeled).”

As this is one of the most common sea whips from southern California, it is not surprising that it has often appeared in live aquarium displays. The Cabrillo Marine Aquarium, in San Pedro, California, had a number of live colonies of this species on display in the public area; all were collected in the local area. As well, the Aquarium of the Pacific, in Long Beach, had a live display of this species in one of its tanks; these also were collected in local southern California areas. However, it is not likely the only species of “whip” that appears in southern California. In any event, it is likely not a common form in northern California. The transitional areas around Point Conception (and waters northward beyond the Bight) offer some intriguing distributional scenarios that will require further exploration. While of similar appearance in general colony form and color, material collected by staff of Olympic Coast National Marine Sanctuary that I examined in Washington State the summer of 2006 (tentatively identified as *Swiftiaspauldingi*) indicated the possibility of several other species of “red whip” along the western United States’ coast. Based on examinations of several other “red whip” forms, it appeared that the upper geographic limit for this species would be Point Conception, California. “Red whips” further north were determined to be one or more different species. This is covered further in the “red whip” discussion included in the remarks made regarding the quintessential “red whip” of the northwestern California coast, *Chromoplexauramarki*.

Looking at location records for specimens collected and identified as *L.chilensis* (with confirmed identification), I noted that if type locality is correct, *L.chilensis* should range from the colder waters off the coast of Chile up through the warmer waters of Central America and Mexico before again encountering the cooler waters of the southern California Bight. How is this possible? What would the depth parameters, substrate features, distance from shore and specific distributional pattern (continuous or fragmented) look like for this species? The missing type material confounds the issue. The material used for Philippi’s (1866) description of *L.rosea* could not be located and no recently collected material from Chile resembling this species is apparently available. Breedy and Guzmán (2007) used California specimens for their description of this species. They “do not exclude the possibility (that) the material from California actually represents another species, but (to date) it most resembles Philippi’s description.” What is needed is a new specimen collected from the Valparaiso area of Chile, so that a definitive neotype could be established, and then used for comparisons. Until that occurs, I have designated a neotype from among the specimens in the SBMNH collection. Perhaps what we now call *L.chilensis* in southern California waters is actually a southern California endemic in need of its own species name, with a far more restricted range than has been implied previously. This is a prime example of a situation where the presumption that any “red whip” found in the southeastern or northeastern Pacific Ocean is likely *L.chilensis* (considered to be quite common), is faulty. The only way to resolve questions surrounding this species is to intentionally examine all “red whip” specimens (in any collection) that may have been collected from California and to intentionally undertake the collecting of material from Chile to southern California, at discrete intervals noting not only latitude/longitude but depth.

In the multiple examinations made of “red whip” forms, a specimen of what had been identified as *Leptogorgiacaryi* Verrill, 1868 was examined. This was a dry specimen from NMNH (USNM 5988), collected at Catalina Island, California. In examining it, along with several specimens of *L.chilensis*, there appeared no marked differences between these specimens, either in overall colony form or in the appearance of sclerites. My initial impression was that *L.caryi* Verrill, 1868 might not stand as a valid species. Interestingly, Breedy and Guzmán (2007) examined the same specimen from NMNH. Independently, they came to the same conclusion regarding *L.caryi* that I did; the specimen was *L.chilensis*, with *L.caryi* a dubious species designation. Cordeiro et al. (2018c) shows *L.chilensis* as an accepted species, while *L.caryi* is designated as nomen dubium. Additionally, *L.caryi* was linked to another “red whip,” *Euplexaura* (now the genus *Chromoplexaura*, Williams 2013a) *marki*, with the species E. (Chromoplexaura) marki being a junior synonym of *L.caryi* (Cairns et al. 2003). The sclerites in this NMNH specimen definitively put it in the genus *Leptogorgia*; sclerites of any specimen identified as E. (Chromoplexaura) marki certainly did not fit the sclerite description of any in the genus *Leptogorgia*. Thus, E. (Chromoplexaura) marki is not a junior synonym of *L.caryi*. This was subsequently verified (ITIS Report, accessed online June 2011). Further, a specimen identified by Nutting (1909) as *L.caryi* was collected near San Francisco (and well above what I believe is the upper geographic limit for *L.chilensis*). This specimen was supposedly deposited in the Museum of Comparative Zoology at Harvard University, but Breedy and Guzmán could not locate it for examination. Based on its collection locality, it would seem that the original identification of this missing specimen is in error. I have concluded that the range of distribution that was stated in the discussions above, and confirmed generally by Breedy and Guzmán, seems to accurately pinpoint where this particular species is found. That it may overlap several other red whip forms in the northern end of its range only emphasizes the need for thorough sclerite examination of specimens collected, particularly in the transitional area from southern to northern California. Future molecular work on “red whips” in the transitional area of southern to northern California may answer the question more definitively.

#### Leptogorgia diffusa

Taxon classificationAnimaliaAlcyonaceaGorgoniidae

(Verrill, 1868)

**Figure 16.**
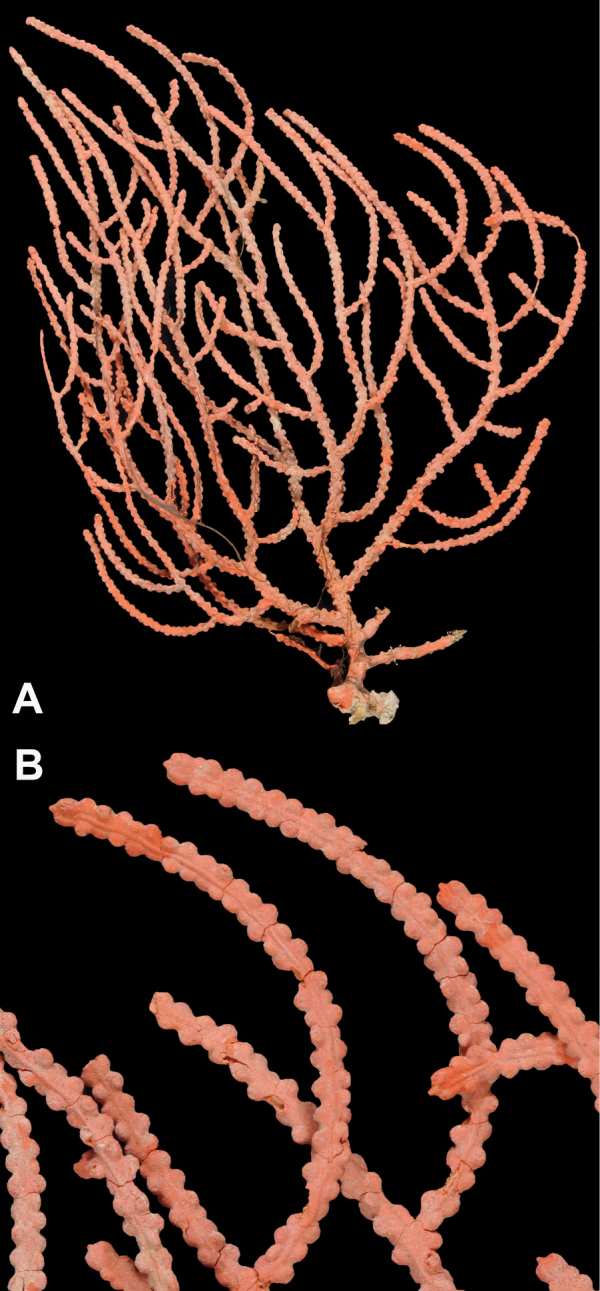
*Leptogorgiadiffusa*, SBMNH 423090. **A** Colony measures 19–20 cm tall, 13.5 cm wide, at widest point **B** Close up, branch tips and placement of polyps on branch surface.

**Figure 17.**
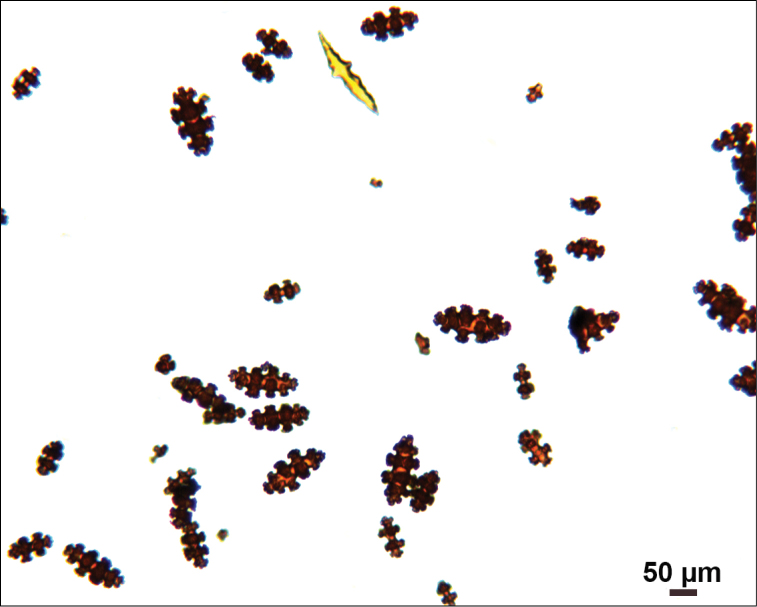
*Leptogorgiadiffusa*, SBMNH 423090, light microscopy, 10×, showing sclerites typical of genus and this species. Note pale yellow anthocodial sclerite, upper middle portion of field, amongst deep red to mauve-pink spindles. Longest sclerites may be part of the anthocodial armature.

**Figure 18.**
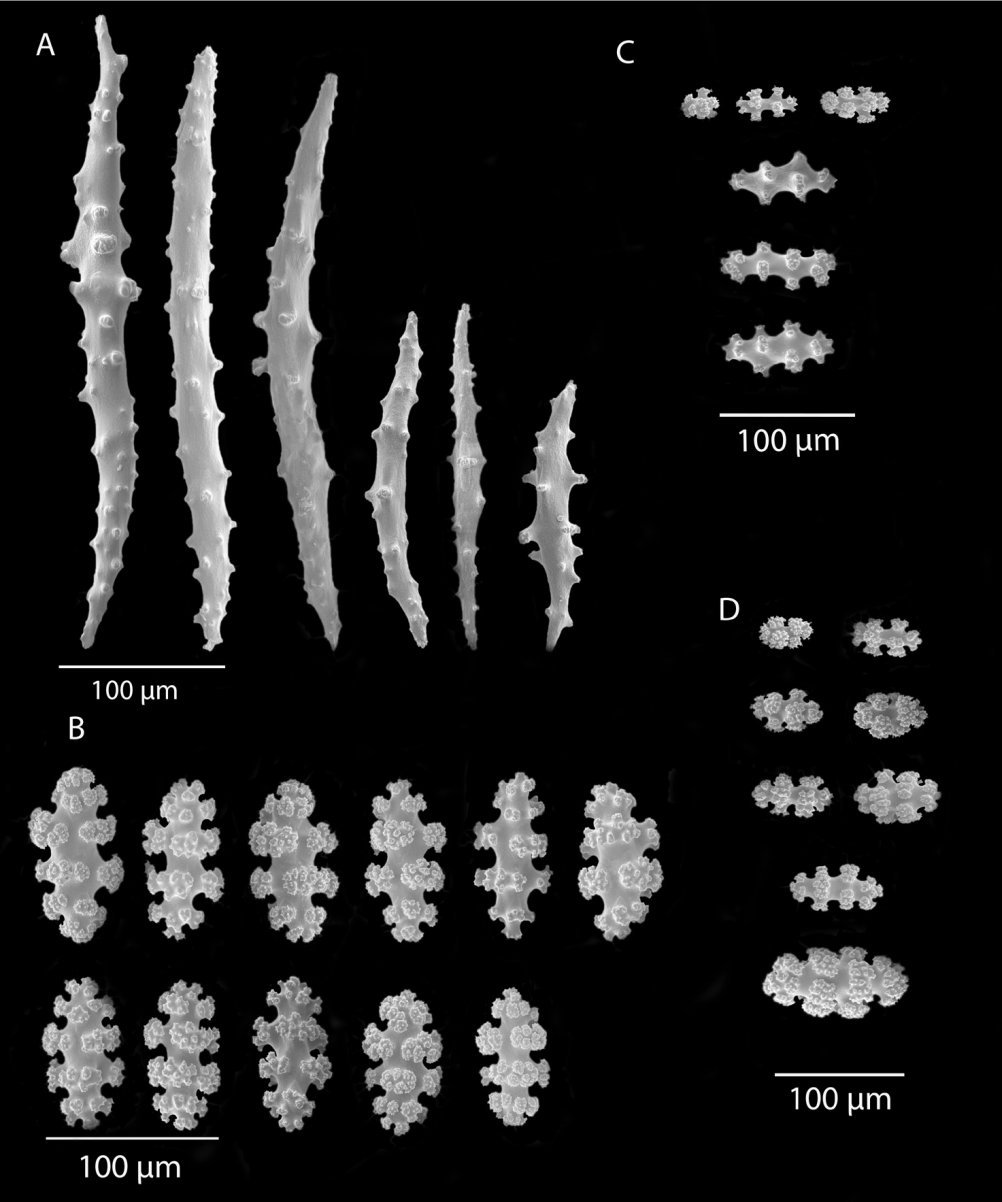
*Leptogorgiadiffusa*, SBMNH 423090, SEM image. **A** Anthocodial sclerites; some were surprisingly and unexpectedly long **B–D** Coenenchymal spindles. Images, for the most part, match those shown in Breedy and Guzmán 2007 (figs 23, 24).

Figures 16A, B , 17 , 18A–D

Litigorgia diffusa Verrill, 1868a; 1868b: 397–398.
Gorgonia (Litigorgia) diffusa Verrill, 1868c: 415.
Leptogorgia diffusa Verrill, 1868a; 1868b: 397–398; pl V, fig. 6; pl VI, fig. 3; 1869b: 421. Nutting 1910d: 5. Bielschowsky 1918: 30; 1929: 112. Kükenthal 1919: 771; 1924: 329–330. Hickson 1928: 413–414. Stiasny 1935: 29. Breedy and Guzmán 2007: 32–37.
Nec Leptogorgiadiffusa: Stiasny 1951: 71 [Guyane Française, Ile Royale] = (Leptogorgiapunicea (Milne Edwards & Haime, 1857) [see Bayer 1961]).
Leptogorgia rubra Bielschowsky, 1918: 29 [nomen nudum]; 1929: 92–94. Kükenthal 1919: 911–912; 1924: 325.
Lophogorgia diffusa : Prahl et al. 1986: 21.

##### Type locality.

(**Lectotype**) Gulf of Panama, Panama, Pearl Islands; additionally (**Paralectotypes**) Gulf of Nicoya, Costa Rica.

##### Type specimens.

**Holotype**, as *Litigorgiadiffusa* Verrill, 1868); YPM 1659a [dry]. **Lectotype**Breedy and Guzmán 2007: YPM 1659 [dry]. **Paralectotypes**Breedy and Guzmán 2007: MCZ 7081 [dry]; YPM 5151 [wet]. Breedy and Guzmán (2007) believe it unlikely that YPM 1659a or 1659b are this species.

##### Material examined.

4 lots (see Appendix 1: List of material examined). I was unable to examine the designated type specimens, but again, distinctive characters of the species made this unnecessary for ID of SBMNH specimens.

##### Description.

*Colony* form (Figure 16A), appearance of branch and polyp placement (with overall effect of polyp placement that of serrated or zig-zag appearance; Figure 16B) and sclerites (Figures 17, 18A–D) correspond with that given in description of the species in Breedy and Guzmán (2007: 32–36), although in the SBMNH material examined, the anthocodial sclerites (Figure 18A) seem exceptionally long.

##### Etymology.

Latin *diffuses*- means spreading, perhaps in reference to open shrub-like appearance that the branches create. No discussion of the species name is given by Verrill.

##### Distribution.

From Panama and Costa Rica to southern California, at least.

##### Remarks.

The lax, flattened branches, large polyps that produce zig-zag appearance, large (and in this case, long) anthocodial rods and the dull brick-red coloring are clear diagnostic features for this species. The species is listed as an accepted form in Cordeiro et al. (2018c).

#### Leptogorgia filicrispa

Taxon classificationAnimaliaAlcyonaceaGorgoniidae

Horvath, 2011

**Figure 19.**
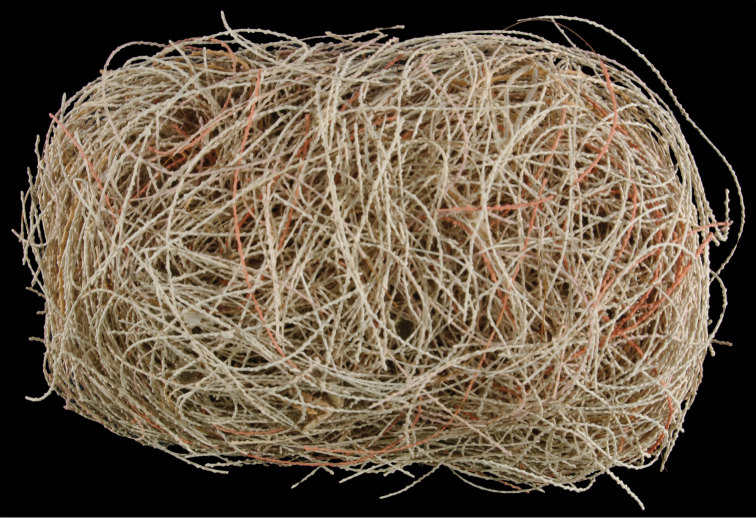
*Leptogorgiafilicrispa*, USNM 1106683-Paratype. Full view of an amazingly large collection of branch strands, showing the multi-colored strands; entire mass measuring 20 cm × 12–13 cm.

**Figure 20.**
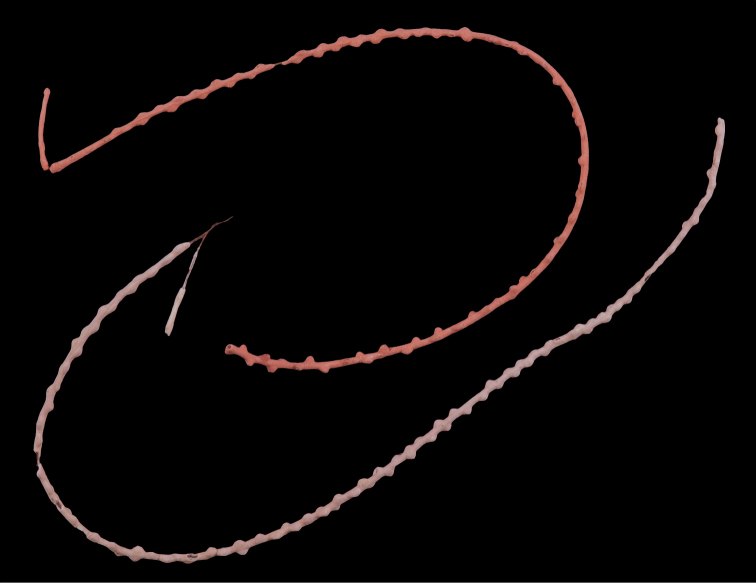
*Leptogorgiafilicrispa*, SBMNH 423057-Holotype. Two single strands, each showing a different pink color variant. Image originally published as figure 1B in Horvath (2011, Proceedings of the Biological Society of WA 124: 1, 47).

**Figure 21.**
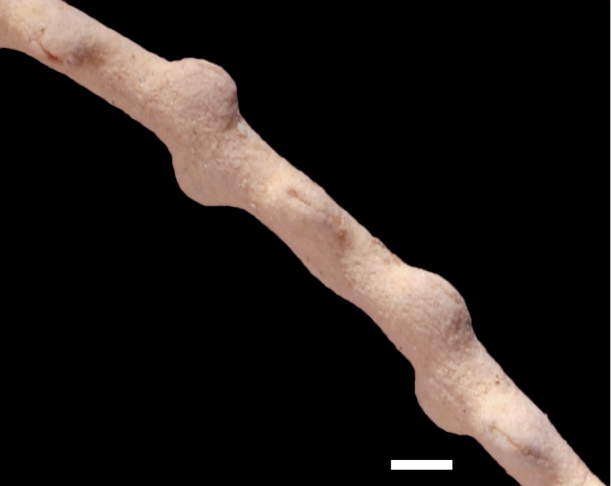
*Leptogorgiafilicrispa*, SBMNH 423057-Holotype. Close-up of branch, showing spacing of polyps; Scale bar: 1.0 mm. Image originally published as figure 1C in Horvath (2011, Proceedings of the Biological Society of WA, 124: 1, 47).

**Figure 22.**
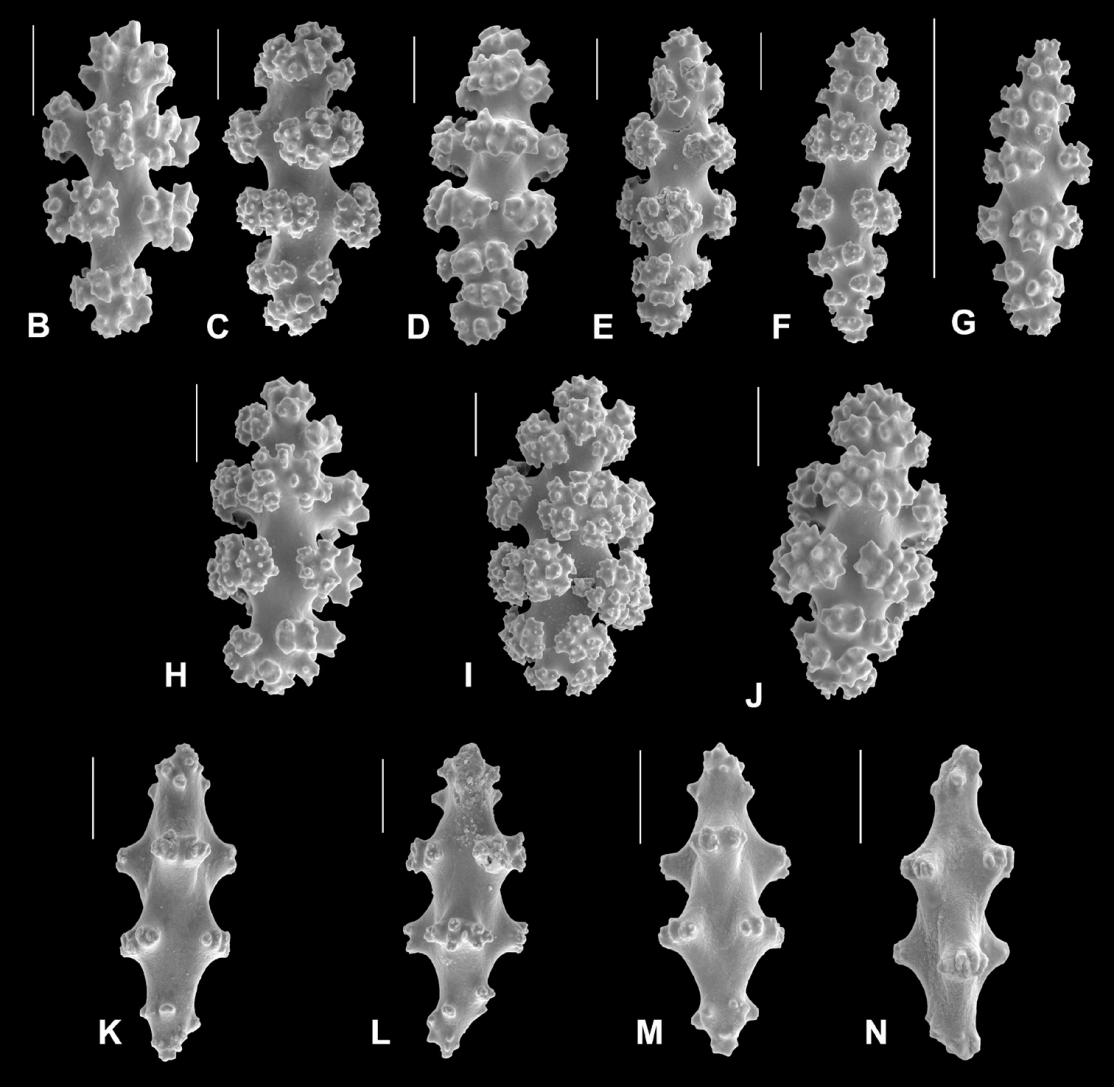
*Leptogorgiafilicrispa*, SBMNH 423057-Holotype. SEM image, originally published as figure 2B–N in Horvath (2011, Proceedings of the Biological Society of WA, 124: 1, page 48). Image prepared by D Geiger, SBMNH. Color of sclerites variable, from white, cream, to pale, bright or deep pink, generally measuring in the range of 55–80 µm; coenenchymal spindles, scale bar 20 µm (**B–F, H–J**), coenencymal sclerite, scale bar 100 µm (**G**); anthocodial sclerites, scale bar 20 µm (**K–N**).

Figures 19 , 20 , 21 , 22B–N

Leptogorgia filicrispa Horvath, 2011: 45–52.

##### Type locality.

Mexico, Baja, California, off Boca Flor de Malva, SE of Punta Tosca, ~24°11'07.04"N, 111°21'03.08"W, 69–87 m.

##### Type specimens.

**Holotype**SBMNH 423057; [dry]; **Paratypes**SBMNH 423079 [dry]; USNM 1106683 [dry]; USNM 1106684 [dry]; USNM 1106685 [dry].

##### Material examined.

~9 lots (see Appendix 1: List of material examined).

##### Description.

*Colony* primarily unbranched; if branched, loosely and little branched in one plane, lateral or pinnate to subpinnate, occasionally dichotomous, not usually bushy; many long (~20–30 cm), slender (0.5–1.0 mm, excluding polyps), whip-like branches (many collected and, presumably, found together as shown in Figure 19), somewhat flattened but never greatly expanded to form lamellar ridges, with free ends more than 50 mm long. Branches very slender, somewhat sinuous from end to end, seeming to curve loosely back on themselves, like fine wire, yet stiff and brittle (Figure 20); tapering towards tips, also very slender. Branches likely grow from these tips; some strands with growth tip at both ends. With specimens available, base seen on several colonies (each colony usually a single strand with none, one or two branches) quite small, and usually affixed to a small rock or pebble; majority of colonies without a base. Not definitively known whether lack of a base (no attachment) is an artifact of collection, or common condition; those with attachment are a more rare situation (no attachment far more commonly seen in collected specimens). Axis very slender; ranging in color from black/dark brown to a translucent brown or reddish brown. Color of living colony, in situ, unknown. In all specimens examined, several uniform color phases were seen. Dry specimen strands exhibited color range from mauve to salmon pink to a much lighter cotton-candy pink to cream to pure white. Polyp-mounds small, conical projections (roughly 1.0 mm in height) on each side of branch (Figure 21); polyps not crowded (1.0–3.0 mm apart), arranged alternately in one or two lateral rows along sides of the branchlets. On some branches, a thin, medial line can be seen running down middle of the flattened branch, between alternate-situated polyps. Sclerites (Figure 22) are spindles, as described by Bayer (1956) for genus, typically with an absence of other specialized forms of sclerite. In this species, spindles thick, tapered; with warts low, rounded; with acute or subacute warted ends, extending beyond a second ring of warts on either side of median girdle. Long spindles generally symmetrical; some with warts on one side simple and conical, elsewhere more complicated. Very few shorter ones with warts of one side fused like those of disc spindles. Flat, tentacular sclerites large; on average sclerites can measure 0.1 mm long by 0.05 mm wide. Generally, sclerites without much color; if colored, typically light pink.

##### Etymology.

The species designation is derived from the Latin root *fili*- for thread, and the Latin root *crispa*- for curled or twisted; designation reflects overall strand appearance, which is thread-like, stiff and wiry; strands of this species reminiscent of the stiff, wiry, curled body of an adult horsehair worm.

##### Common name.

Multi-colored wire gorgonian.

##### Distribution.

Based on collection locations for specimens in SBMNH collection, LACoMNH collection and those examined in collection at NMNH, from at least Ventura, California south to coast of Baja, into Gulf of California. Perhaps southern end of the California Bight is the northern limit for this species.

##### Biology.

A comment was made (Grigg 1972) about gorgonian colonies having “a loosely branched and whip-like shape when located in circular basins where water flow is turbulent.” Apparently this condition can be seen on shallow reefs to depths of 25 m (Grigg 1972). In this instance, it is suspected that regardless of water motion, the species consistently displays this distinctly thread-like form. Noteworthy point: an Atlantic form, *Leptogorgiastheno* Bayer, 1952, is normally unattached to any substrate; generally, many of the strands in the SBMNH material appear to exhibit that condition, as well. Having never seen this species in situ, and with no confirmed reports from other observers, thus far, nothing more can be stated about this species’ biology.

##### Remarks.

The species as described (Horvath 2011), shown as an accepted species in Cordeiro et al. (2018c), had strong similarities to *Leptogorgiasetacea* Pallas, 1766 and shared many with *L.stheno* Bayer, 1952, both of which are species found in the western Atlantic. Differences were apparent in geographic location, coloring of sclerites, and subtly, in branch diameter; may be a twin species of an Atlantic form. Based on numerous strands in the NMNH material as well as material from Boca Flor De Malva at SBMNH and LACoMNH (and two other examples, both dry that were examined [see Appendix 1: List of material examined, Other material examined], one specimen from SW Punta San Juanico, outer coast, Baja, California and the other from deep water off Redondo Beach, California, US), it is possible that this species is actually very common. While perhaps not very obvious, looking nothing like the standard of a sea fan (or even a sea whip), it is suspected that this species is routinely overlooked or regarded as nothing more than a batch of dead coralline algae. Further collection is necessary, and when found, depending on environmental conditions, the question of colonies attached or not should be addressed.

#### Leptogorgia flexilis

Taxon classificationAnimaliaAlcyonaceaGorgoniidae

(Verrill, 1868)

**Figure 23.**
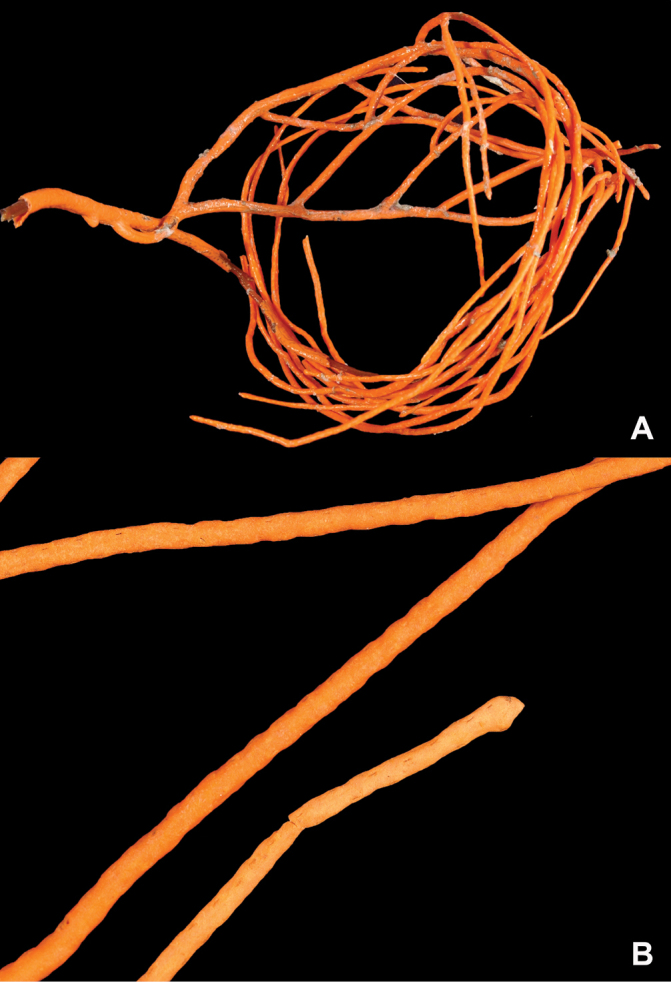
*Leptogorgiaflexilis*, SBMNH 422941. **A** shows colony color, and interesting disposition of branches, with tendency to droop. Colony, gently extended, ~30–40 cm tall **B** Close up of several branches. Note marked point of branch tip on end of lowest branch.

**Figure 24.**
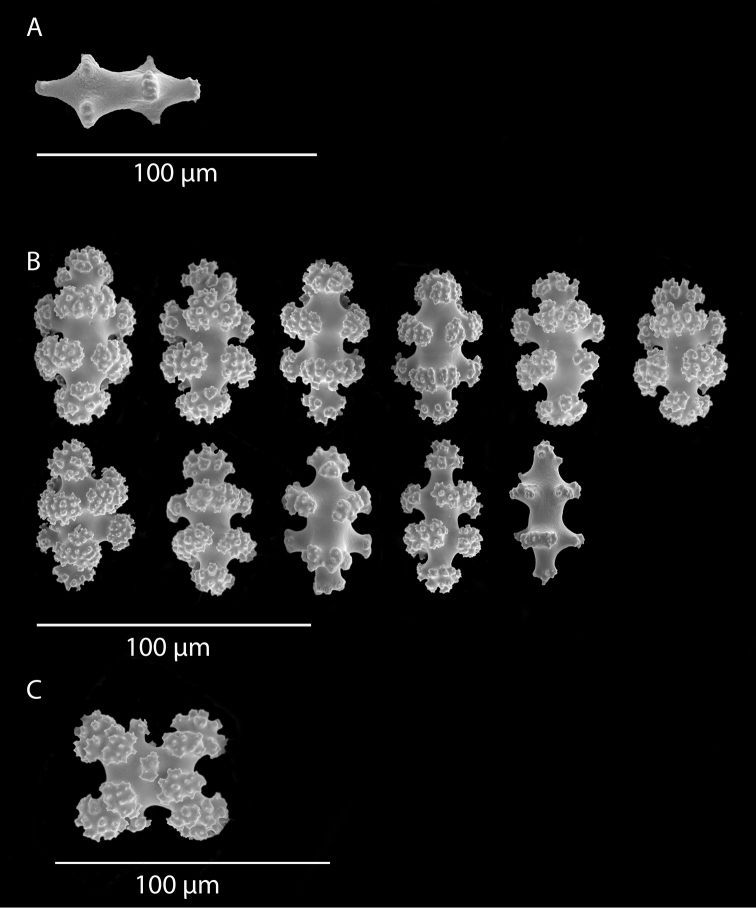
*Leptogorgiaflexilis*, SBMNH 422941, SEM image. Sclerite color deep orange. **A** Anthocodial sclerite **B** Sclerites of coenenchyme (last of which may actually be an anthodocial sclerite) **C** Quadriradiate from coenenchyme. Images match those shown in Breedy and Guzmán 2007 (fig. 30).

Figures 23A, B , 24A–C

Gorgonia (Eugorgia) flexilis Verrill, 1868c: 415.
Litigorgia flexilis Verrill, 1868a; 1868b: 400–401.
Leptogorgia flexilis Verrill, 1868b: 400–401; pl V; fig. 11; 1869b: 421. Nutting 1910d: 5. Bielschowsky 1918: 29. Kükenthal 1919: 771; 1924: 326. Hickson 1928: 414–416. Bielschowsky 1929: 96. Stiasny 1943: 82. Breedy and Guzmán 2007: 40–44.

##### Type locality.

Archipelago Las Perlas, Panama, 11–15 m.

##### Type specimens.

**Syntypes**Breedy and Guzmán 2007: YPM 1553a, b [dry]; MCZ 4123 (722) [dry].

##### Material examined.

5 lots (see Appendix 1: List of material examined). Designated types not examined.

##### Description.

An examination of SBMNH material revealed that colony form (Figure 23A), branch and polyp appearance (Figure 23B) and sclerites (shown here, Figure 24), are comparable with images shown in Breedy and Guzmán (2007: 40–44).

##### Etymology.

The root *flexi*- is Latin for pliant, bendable, referring to the apparently flexible, droopy, slender branchlets of the live colony. However, Verrill does not give any rationale for the species name.

##### Distribution.

Panama, north into lower third of California Bight (off Santa Catalina Island and adjacent California mainland sites).

##### Remarks.

Initially, the drooping branches were considered to be more an artifact of preservation and the containers initially used when collected (branches bent downward so specimen would fit in the jar). However, descriptions by others (Breedy and Guzmán 2007), indicated that this is a normal branch configuration. Initial preservation in harsh chemicals caused drooping, pliant branchlets to become anything but; now quite brittle and easily broken. SBMNH 422942 is a nice, large colony but badly fragmented due to those early preservation efforts. As well, much of its color has leached out, with any particular branch colored from almost white to tan to pinkish red.

*Leptogorgiaflexilis* is an accepted species in the WoRMS Data Base (Cordeiro et al. 2018c).

#### Leptogorgia

Taxon classificationAnimaliaAlcyonaceaGorgoniidae

species A

**Figure 25.**
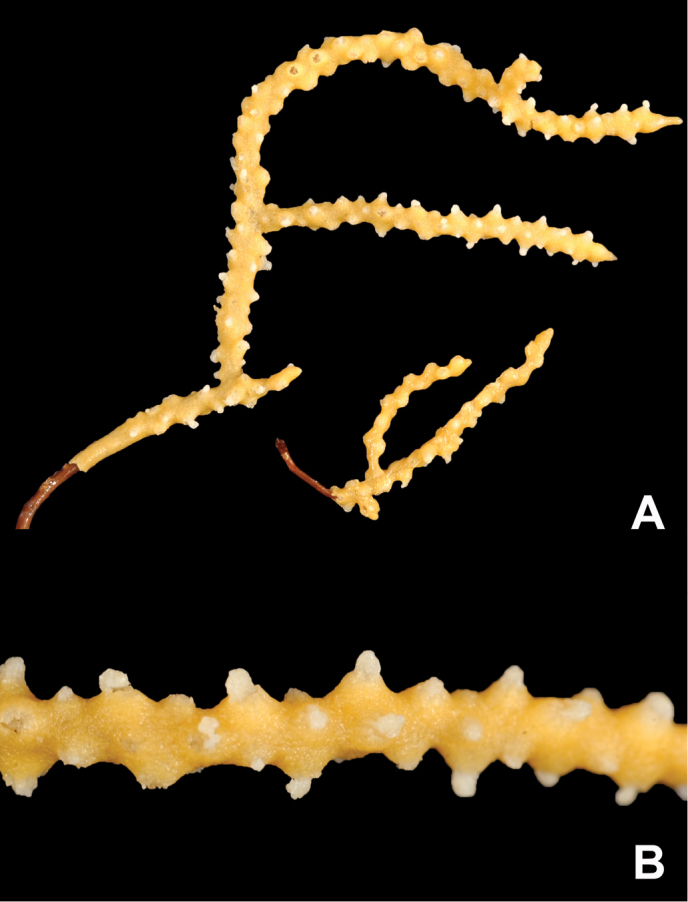
*Leptogorgia* species A, SBMNH 423080. Two specimens from same lot. **A** shows branching, the tortuous condition seen in some branches, and bright yellow color, with prominent calyces/polyps. Larger colony measures 6.0 cm × 3.0 cm **B** Close up of branches, showing bright yellow coenenchyme and prominent, conical mounds with white polyps.

**Figure 26.**
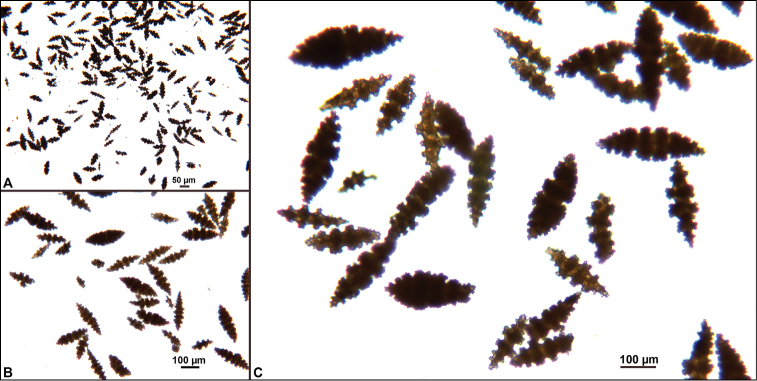
*Leptogorgia* sp. A, SBMNH 423080, Light microscopy image. All sclerites in SBMNH material are deep yellow in color. **A–C** Increasing magnifications of coenenchymal sclerites. In some specimens examined, the longest sclerites measured ~280–360 µm, and the smaller, more slender sclerites measured somewhere between 180–260 µm.

**Figure 27.**
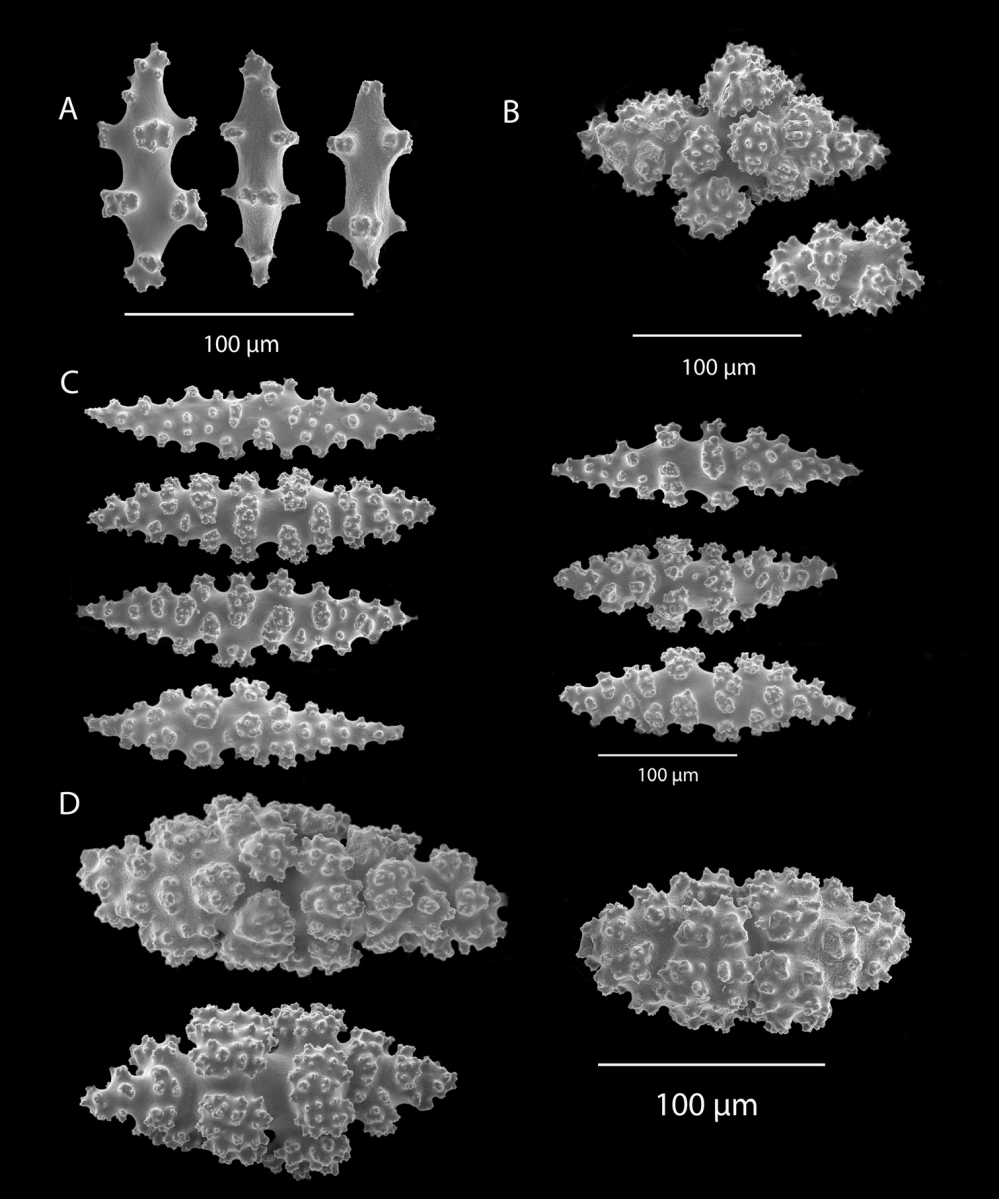
*Leptogorgia* sp. A, SBMNH 423080, SEM image. **A** Anthocodial sclerites **B** Coenencyhmal sclerites of odd shape **C–D** Coenenchymal spindles. Compare this SEM with SEM images shown in Breedy and Cortés (2011).

Figures 25A, B , 26A–C , 27A–D

[? = LeptogorgiatricorataBreedy and Cortés 2011]

##### Type locality and type specimens.

There is a need for further confirmation of species identification regarding SBMNH specimens, through examination of other definitively identified specimens, as well as the type specimens for *L.tricorata* (**Holotype UCR 1833; Paratypes UCR 1834, 1835, 1836** and **1837**). The holotype was collected in Cocos Island National Park, Isla Manuelita NW, taken on 8 September 2006 at a depth of 14 m. The paratypes were collected from Cocos Island National Park as well, either at Isla Manuelita or Roca Sucia.

##### Material examined.

~11 lots (see Appendix 1: List of material examined).

##### Description.

*Colonies* (Figure 25A) non-reticulate; main stem ~14 cm long, arising from thin, flat attachment structure; latter gives off generally dichotomous (or irregular), mostly lateral, few to moderate, elongated, sometimes slightly crooked branches; these may divide again, often not; upright growth pattern in most, overall giving colony the appearance of a candelabra. Stem and branches rounded, nearly uniform, 1.0–2.0 mm diameter, not including polyps. Branches bend outwards in broad curve at axils; terminal branches from 2.5–7.5 cm long, without division, blunt at end. Few branchlets, rounded and slightly crooked. (One lot, SBMNH 422334, a simple, single whip-like, unbranched to minimally branched fragment, where diameter tends to smallest measurements of range, length ~37 cm, but not complete; other fragments much shorter, as above). Stem, branches and branchlets covered on all sides with prominent conical polyps, when extended (Figure 25B); when contracted, nearly flush with branch surface; apertures circular. Polyps measure 0.2 mm tall (extended), 1.7 mm wide; spacing between them 2.0–2.5 mm apart. Arrangement of polyps does not delineate median groove. Color of all colonies, regardless of colony shape, bright lemon yellow or gold; most sclerites bright lemon-yellow or gold; the few straight, less warted sclerites, pale or colorless. Sclerite shapes (Figures 26A–C, 27A–D) not diverse; mostly spindles, heavily warted; warts form regular belts; belts either evenly spaced (six to seven belted rings) or belts much closer together, largest at middle of spindle and outwards toward spindle tips progressively smaller, creating in silhouette sclerites that appear in elongated diamond shape (Figures 26C, 27C); some few (Figure 27D) of these with dense triangular collection of warts at each end with very narrow, median waist; very few straight, not as heavily warted, spindles. In a comparison with images from Breedy and Cortés (2011, Figure 2), similarities between the sclerites shown in their image and the one included here in Figures 26C and 27 are strong, with exception of tentacular sclerites (rods); SBMNH specimens may be *L.tricorata* Breedy & Cortés, 2011.

##### Distribution.

From specimens examined within the California Bight, limited range from Cortes Bank up to California Channel Islands, but see also Breedy and Cortés (2011) and “Remarks” below.

##### Biology.

Barnacle galls present on a number of specimens (SBMNH 423084 and SBMNH 422903).

##### Remarks.

This assemblage of specimens still not identified with certainty; despite the apparent similarity with *Leptogorgiatricorata* Breedy & Cortés, 2011, it seemed unlikely that a species from the shallow waters of Cocos Island would be seen in the California Bight. Yet, its species name, using an adjective derived from the Latin root *tricoratus*-, meaning to make tricks, is applicable, as no one I spoke to who regularly collects within the Bight (LACSD, OCSD) recalled ever seeing this species. Nearly all specimens in the SBMNH collection were collected in southern California in 1940 and 1941. A few specimens more recently collected (recent being late 1970s) are also included in the collection. Since then, however, no specimens that might be this species have been encountered or reported in any collecting events to the present. All specimens examined have slightly thicker branch diameter than that seen in *Thesea* Duchassaing & Michelotti, 1860 (which they can resemble on a superficial level; this especially true of fragments of SBMNH 422334; one other specimen in collection, SBMNH 13304, from a station off Point Loma, is identical), and color that is generally bright lemon yellow-gold, with very markedly colored sclerites, which display a very angular, elongated diamond-shape (refer to Figures 26C, 27C here and Figure 2 in Breedy and Cortés 2011). None of the large, spheroidal bodies common to *Thesea* were seen in these specimens; *Thesea* was eliminated as a possibility. What prevents a positive identification (as *L.tricorata*) was a difference in aperture shape (circular vs. oblong) when polyps are contracted and complete absence of the tentacular rods, characteristic of *L.tricorata*. Multiple sclerite arrays were prepared, none of which displayed even a hint of the tentacular rods. While the material is older, it is in very good shape, having always been kept as wet specimens, with no evidence of formalin contact. Until further specimens can be found and collected from waters in southern California, within the Bight, and thoroughly examined, it seemed best to place these in the genus *Leptogorgia* without species designation (*L.tricorata* does seem a strong possibility; however, lack of tentacular scleritic rods is problematic).

An additional piece of information regarding *L.tricorata* can be found in the work of Soler-Hurtado et al. (2017). Based on molecular analysis, they have proposed that *L.tricorata* should now be considered a derived form in the genus *Pacifigorgia* Bayer, 1951 (see page 226, Soler-Hurtado et al. 2017). Cordeiro et al. (2018d) does not show this proposed emendation of the genus *Pacifigorgia* in the WoRMS Data Base; *L.tricorata*, however, is shown as an accepted species in the genus *Leptogorgia*. I would add that the branching morphology in the SBMNH specimens is not reflective of branching patterns usually seen species of *Pacifigorgia*.

### 

#### Family Plexauridae

Taxon classificationAnimaliaAlcyonaceaGorgoniidae

Gray, 1859

##### Diagnosis.

Colonies of very diverse form, generally with thick branches arising laterally, dichotomously (in some, pinnately). Polyps completely retractile or forming distinct calyces into which anthocodiae can be withdrawn. Axis with wide, chambered central chord; peripheral zone of loculated horny material, usually containing nonscleritic calcareous matter (common tendency toward heavy calcification of base in old colonies). Coenenchyme thick, perforated by system of longitudinal canals surrounding axis, delimiting outer coenenchymal layer from inner one (axial sheath), which differ in spiculation. Sclerites usually include some form of club; some with spindles only, oval bodies, rods or large quadriradiates.

##### Remarks.

Due to the highly variable nature of genera and species placed in this family, this is a complex, often confusing group of organisms. Ultimately, the best means to understanding this family was to study, in total, each of the several genera placed in it that are seen in California waters. In this part (Part II), emphasis has been placed on *Chromoplexaura* (formerly *Euplexaura*) *marki* Williams, 2013a (part of a collective group referred to as the “red whip” species) and genera *Muricea* Lamouroux, 1821 and *Placogorgia* Studer, 1887 (Wright and Studer 1889). While the genera *Swiftia* Duchassaing & Michelotti, 1864 and *Thesea* Duchassaing & Michelotti, 1860 are also included in this family, it was necessary to cover those with a more extensive study, discussed in Part III. The genus *Thesea*, as represented in California waters, additionally requires still further examination; ongoing study of that genus is in progress, and will require a separate discussion, to be presented at a later date.

#### Plexaurinae

##### Family Plexauridae [= Muricidae]

###### Genus Chromoplexaura

Taxon classificationAnimaliaAlcyonaceaPlexauridae

Williams, 2013

Chromoplexaura Williams, 2013a (part): 31, 34–35.

####### Type species.

*Euplexauramarki* Kükenthal, 1913.

####### Diagnosis.

Tall, erect, generally planar colonies, bright red; if branched, lateral (not extensively branched, if present, at all), from single, basal stem. Upper branches slender, elongate, most slightly curved, distally less dense; denser proximally and lower in colony. Polyps fully retractile; on all sides of branches and stem, as numerous slightly rounded, low to flat protuberances. Sclerites also red; robust spindles, and radiates, some ellipsoidal to sub-spherical in shape; prominent sclerite a long spindle with prominent, cone-shaped caps at each end and obvious median “waist” (herein referenced with new terminology: the double-dunce cap or double-dunce). Contains a single species from the temperate eastern Pacific (generally, California to Washington; slight possibility of presence in Canadian (even Alaskan) waters).

####### Etymology.

Derived from the Greek *chroma*- referring to color, and the gorgonian generic name *plexaura*- in reference to the bright color of the colonies.

####### Remarks.

Diagnosis for the genus *Euplexaura* (Kükenthal, 1913a) was examined for comparison with that of the recently proposed genus *Chromoplexaura* Williams, 2013a. The species placed in this new genus is well represented in the SBMNH collection, fitting the description given by Williams (2013a, 32–39). The original placement of the temperate Eastern Pacific species in the genus *Euplexaura* was adhered to for an entire century, based on the original description of Kükenthal (1913a). That original description was little referenced, and specimens of the species in this genus collected along the California coast post-1913 were often misidentified. Both the genus (as represented in CA) and the locally collected species received virtually no further attention until my work began on the SBMNH collection in 2002; a subsequent inquiry of Dr Williams (e-mail conversation, March 2011) was made, regarding what his perspective on the species was. Williams’ (2013a) establishment of a new genus for this temperate gorgonian is justified.

###### Chromoplexaura marki

Taxon classificationAnimaliaAlcyonaceaPlexauridae

(Kükenthal, 1913)

Figures 28 , 29A, B , 30A, B , 31A–C , 32A, B , 33A, B , 34A–C , 35A–E , 36 , 37A1–37 , 4 , 1B , 2B , 3 , 38A–E

Euplexaura marki Kükenthal, 1913: 266–269; text figs G, H, J, K, pl 8 fig. 11; 1924: 93–94.
Chromoplexaura marki (Kükenthal, 1913): Williams 2013a: 36–39; figs 12–17.

####### Type locality.

For the original specimen, USA, Southern California, 64–616 m. (Identification cannot be confirmed.) For proposed **Neotype**, collected in Northeastern Pacific, USA, California, Monterey County, Monterey, BLM Reference Station 360 (Burch #40128), ~22 m; coll. T Burch, 18 August 1940.

####### Type specimens.

Repository for the original type specimen unknown. Proposed **Neotype** (designated here), SBMNH 423060 [dry].

####### Material examined.

~60 lots (see Appendix 1: List of material examined).

####### Description.

**Figure 28.**
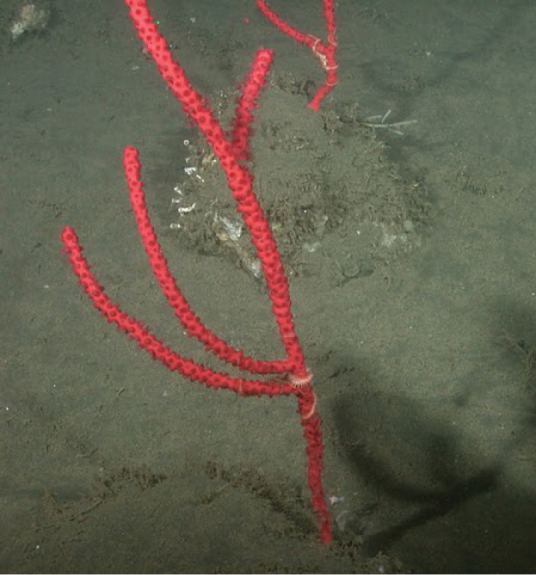
In situ image of what appears to be *Chromoplexauramarki*, DSCN5297. Colony seen off Oregon coast. Without initial examination of sclerites, originally thought to be *Swiftiasimplex*; examination of sclerites aligned it with *C.marki*. There can be remarkable similarity in external gross appearance between the two species. Image courtesy of Peter Etnoyer, NOAA, 2010.

**Figure 29.**
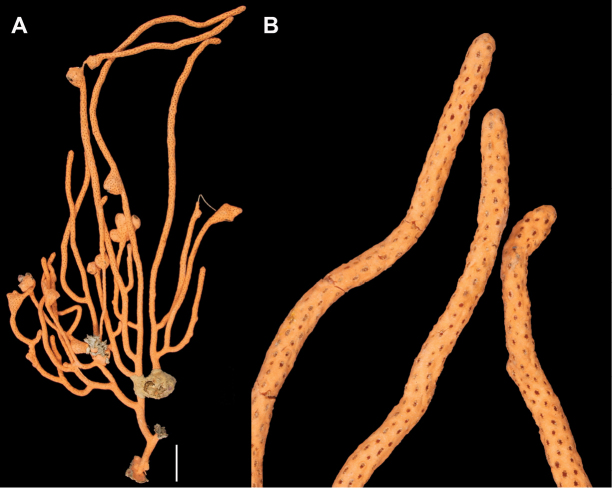
*Chromoplexauramarki*, SBMNH 423060 (Neotype). **A** This specimen, measuring 24–26 cm at greatest length, bears two different types of barnacle, a species that appears to be that of an acorn barnacle (forming the galls on this specimen), and clusters of a barnacle species that appears to be a “*Lepas*-type.” The sclerites from this colony aligned with known specimens of *C.marki***B** Branch tips at greater magnification.

**Figure 30.**
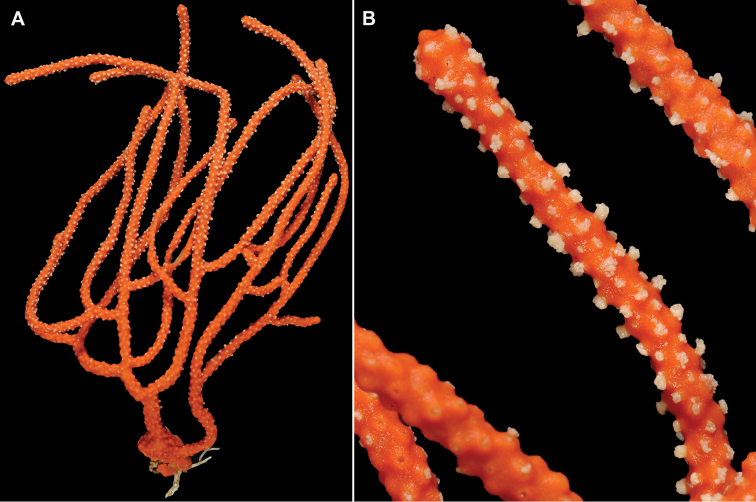
*Chromoplexauramarki*, SBMNH 423062. **A** Specimen (whole colony). Sclerites did not always consistently match sclerites seen in the species, illustrating variable nature of sclerite forms. Colony measures ~16 cm × 10 cm **B** Close up, branches and branch tips, clearly showing bright red-orange color and distinctly white polyps of the species *C.marki*.

**Figure 31.**
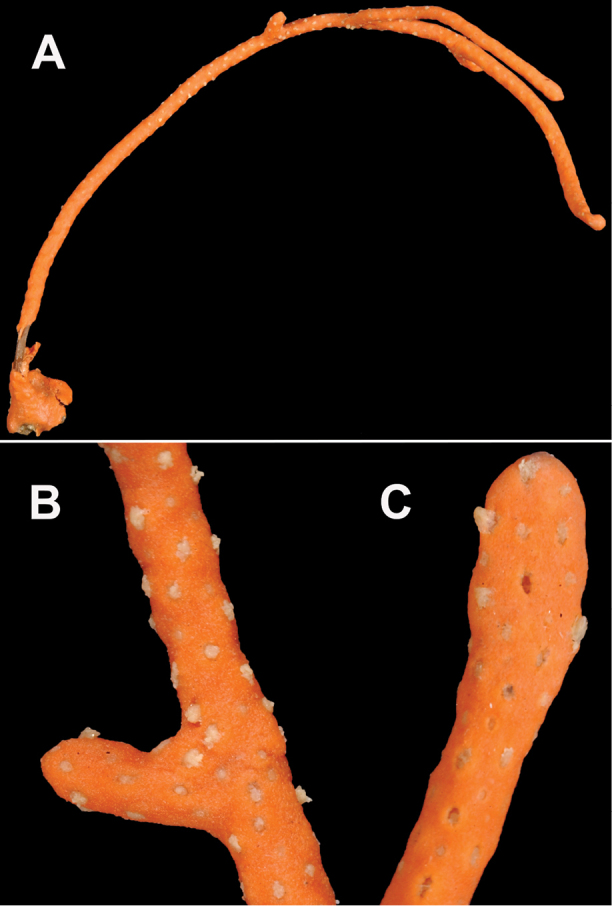
*Chromoplexauramarki*, SBMNH 423072. **A** Colony with coenenchyme of a bright orange-red color with conspicuous white polyps. Sclerites did not always consistently match sclerites seen in the species, illustrating variable nature of sclerite forms. Specimen measures ~15.5 cm × ≤2 cm **B** Branch close-up, showing conspicuous white polyps **C** Close up of branch tip.

**Figure 32.**
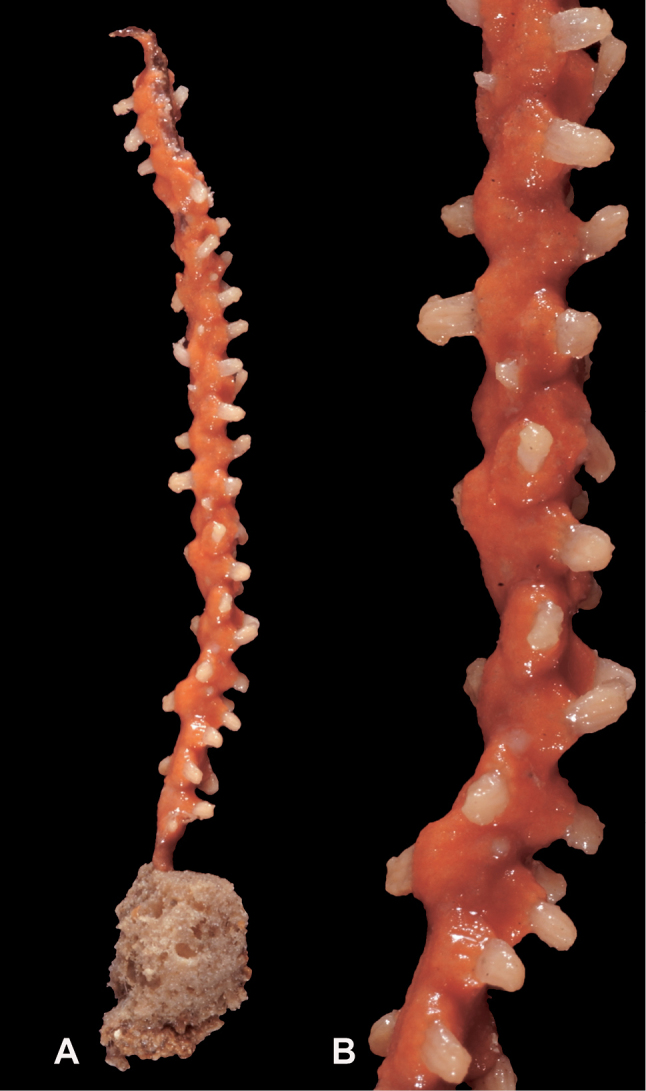
*Chromoplexauramarki*, SBMNH 265948. **A** Very small colony, 4.0 cm in length, excluding mass at base, which appears to be a sponge. Sclerites did not always consistently match sclerites seen in the species, illustrating variable nature of sclerite forms **B** Close up of extended polyps on single branch, each measuring ≤ 1.0 mm in height.

**Figure 33.**
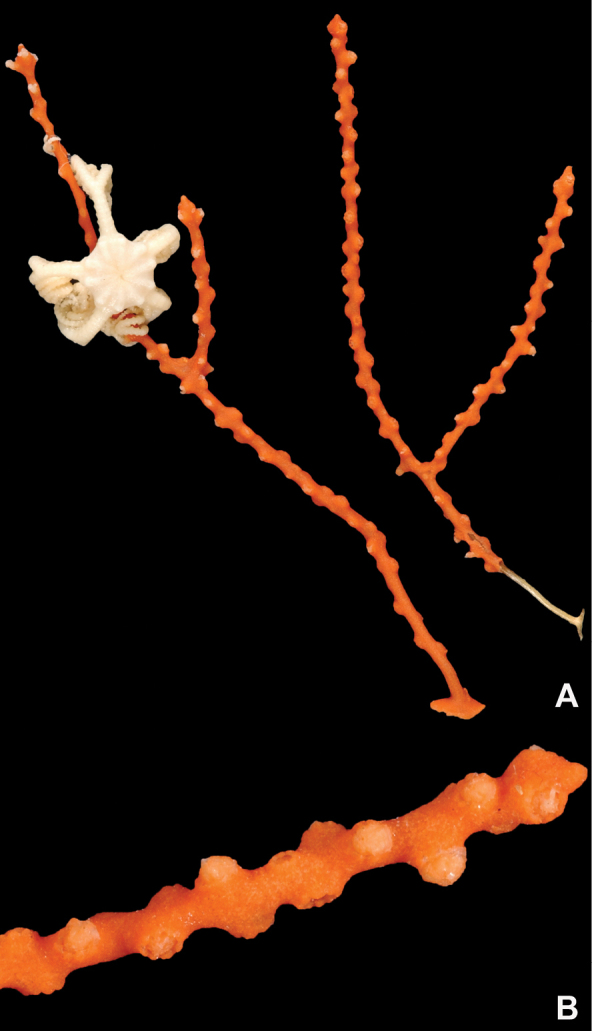
*Chromoplexauramarki*, SBMNH 265935. **A** Two colonies, larger of the two (only 7.0 cm tall) with conspicuous brittle star attached. Sclerites did not always consistently match sclerites seen in the species, illustrating variable nature of sclerite forms **B** Close up of branch tip, showing placement of calyces, color of polyp body and tentacles.

**Figure 34.**
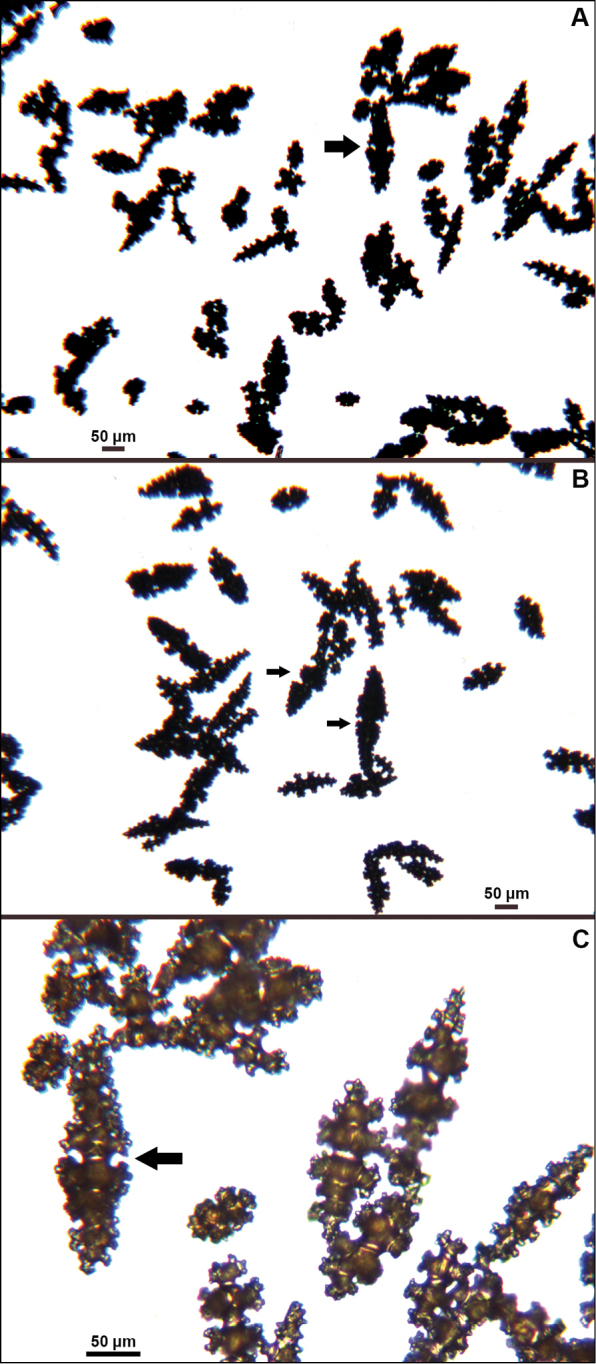
*Chromoplexauramarki*, SBMNH 423060, light microscopy arrays. **A** 4× magnification. Note in particular the “triangular-capped” spindle designated with arrow. This sclerite form is never seen in specimens from the genus *Swiftia* (some species in genus *Swiftia* can look superficially like this species in overall colony form) **B** Additional image at 4× magnification of sclerites, illustrating further examples of “triangular-capped” spindles appearing commonly in *Chromoplexauramarki***C** 10× magnification of sclerites, showing variety, with particularly clear example of the “triangular-capped” spindle, indicated with arrow.

**Figure 35.**
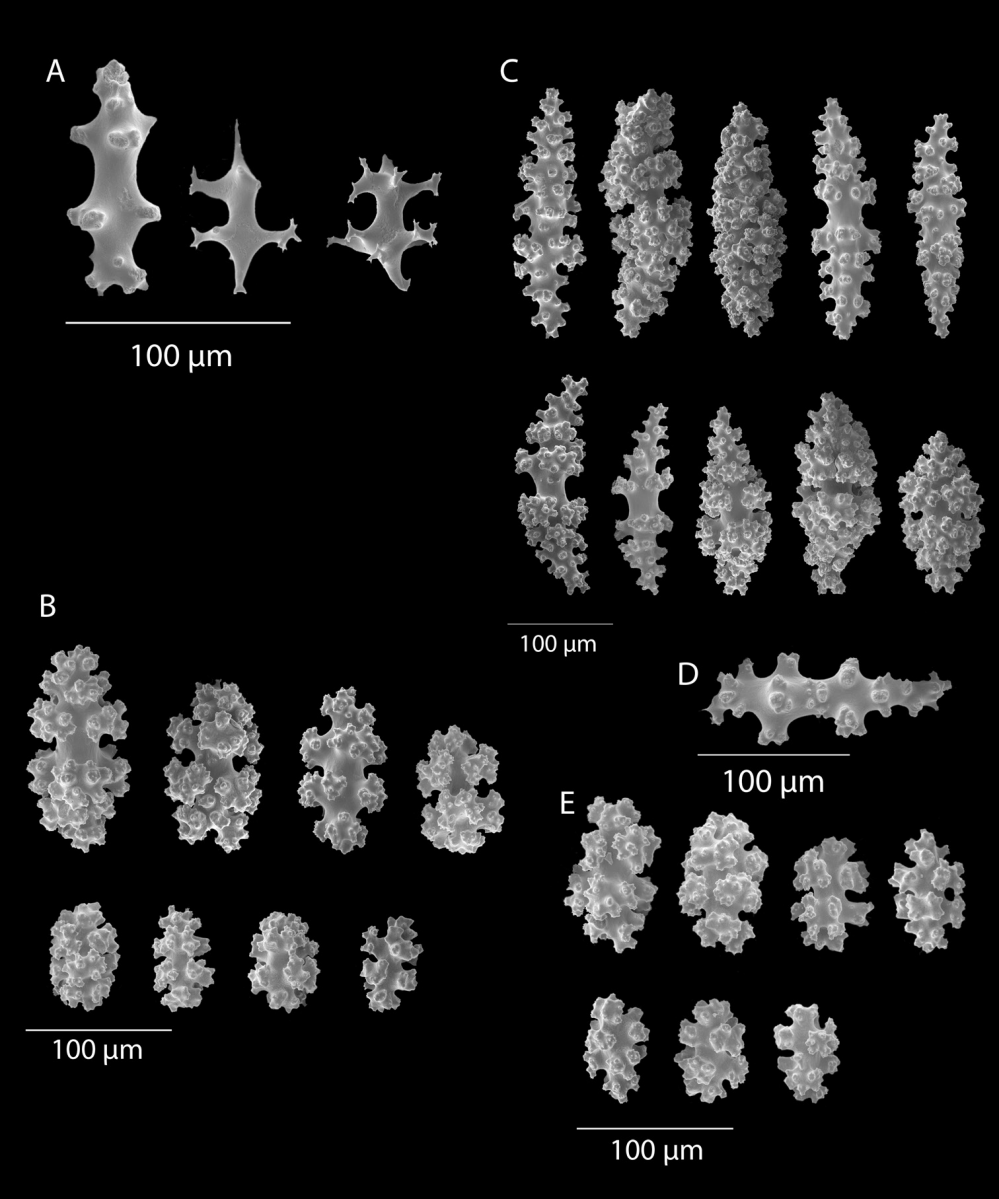
*Chromoplexauramarki*, SBMNH 423060, SEM image. **A** Anthocodial sclerites **B** Small coenenchymal forms **C** Larger, distinctive “double dunce-cap” spindles **D** Odd spindle **E** Still smaller coenenchymal sclerites. Images match variety shown in Williams 2013a (figs 14–17).

**Figure 36.**
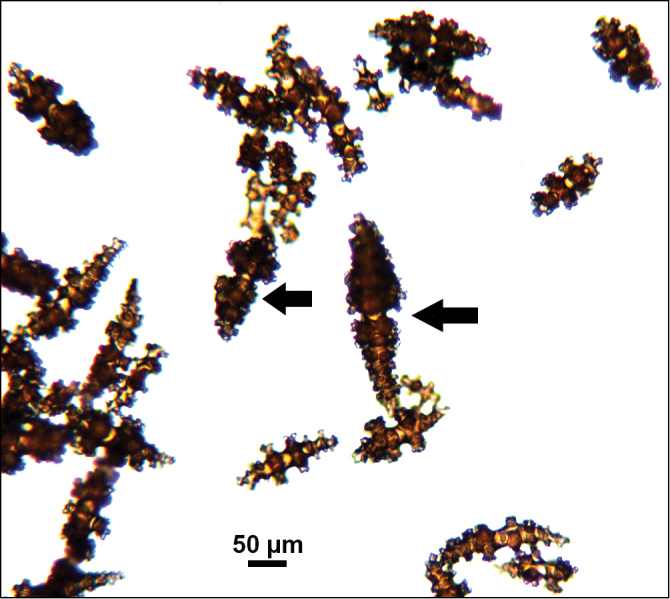
*Chromoplexauramarki*, SBMNH 423062, light microscopy array of sclerites, 10× magnification. Two sclerites, marked with arrows, are those that define the species (compare with those shown in Figure 34). The large central sclerite, just to left of image center, is > 100 µm in length, while non-capped spindles measure 100–130 µm.

**Figure 37.**
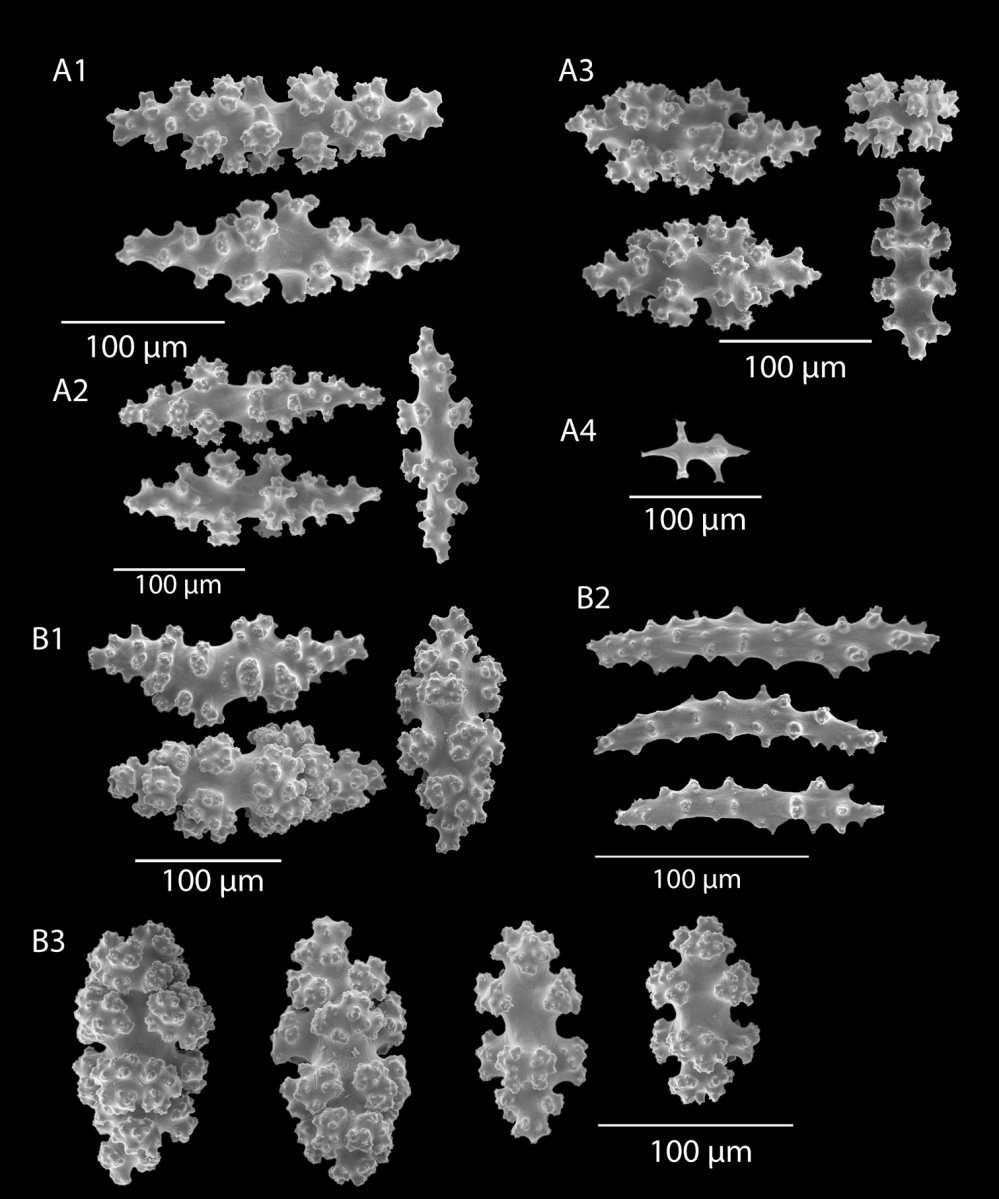
*Chromoplexauramarki*, SEM images. **A**SBMNH 423061 **A1** Double dunce-cap forms **A2** Slightly smaller double dunce-cap forms **A3** Coenenchymal sclerites **A4** Anthocodial form **B**SBMNH 423072 **B1** Double dunce-cap sclerites **B2** Elongated spindles from coenenchyme **B3** Coenenchymal sclerites. Images match variety shown in Williams 2013a (figs 14–17).

*Colony* (Figures 28, 29A, 30A, 31A, 32A, 33A) shape can be a wide, broad, moderate to sparsely branched fan, typically in one plane or simple, unbranched; initial branching lateral, progressing to branches that tend to project more up than out; sometimes projecting/winding more in a “front” or “back” direction; with broader membranous base; main branches can divide repeatedly, all secondary branches off larger ones having same diameter; round in cross-section; distal ends often slightly swollen; see Figures 29B, 30B, 31B, C (See Remarks below for further discussion of overall colony shape). Axis proteinaceous, generally well calcified, not very flexible, with hollow core. Color of axis variable between white, yellowish, and light brown. Color of living colony base, stem and branches bright coral red (orange-red), bright red, or dark red; red color enhanced by color of the sclerites, which are a bright, pale, transparent red. Polyps are (pale) yellow-colored when living; (Johnson and Snook (1927) stated the color of *E.marki* to be coral-red, with the living polyps yellowish white). When polyps visible in preserved specimens, they appeared white/cream. Coenenchyme moderately thick, rising in wall of polyp as eight very short folds. Polyps sit ~2.0 mm distant; polyps ~2.0 mm high, 1.0 mm broad, when extended; polyps fully retractile into coenenchyme surface, forming very low, rounded bumps (“polyp-mound”); aperture suggestive of a goblet/chalice shape; fortification of polyps weak. Kükenthal (1913a) stated that there are no calyces in this species, or at least are so negligible as to be virtually nonexistent (in describing *Euplexauramarki*, he indicated that there should be two transverse rings of large sclerites, one over folds in the polyp head and a second just below insertion of the tentacles). Sclerites of polyp body red, while sclerites of tentacles smaller, colorless, transparent, but of similar form. Occasionally, tentacle sclerites can be a bent spindle (Figures 34A–C, 35A–E, 36, 37A1–4, 1B–3, 38A–E). Found initially in a specimen identified as this species (USNM 51500) was a sclerite form that was not clearly featured in sclerite descriptions for the genus, or other known species (Figures 34A–C, 35C, 36, 37A1, B1, 38B): in outer surface of branch coenenchyme lie sclerites whose basic form is a thick spindle, but on these sit two or more belts of very big, jagged warts. Through the development of these large warts, the spindle can have a contour that is nearly oval (these might be the tuberculate spheroids mentioned in original description; apparently a key sclerite form, for the genus *Euplexaura*, at least) to a distinct diamond shape. Occasionally, one can find on both ends of these spindles, dense triangular caps (Figure 34A–C, 35C, 36, 37A1, B1, 38B) separated by a smooth, usually thin, median waist, so that in this case, the term double spindle (double-head) could apply; these are characteristic and conspicuous of multiple specimens examined, and henceforth referred to as the double-dunce cap. Size of these outer spindles fluctuated considerably, with smallest only 0.05 mm in length, but often bigger (~0.2 mm). Those deeper into coenenchyme of branches had similar form, but warts were more rounded (Figures 35B, 37A3, B3, 38C). All of the more superficial sclerites are red, making the strong bright red color of the colony fairly pronounced.

####### Etymology.

Species named in honor of EL Mark of Harvard University.

####### Common names.

MBARI (seen in a hall display, Summer 2008) referred to this species (and to any species from northern California appearing as a “red whip”) as “Red licorice gorgonian”.

####### Distribution.

Southern California; littoral and coast-abyssal (Kükenthal, 1924, as *Euplexauramarki*). Johnson and Snook (1927) noted the species living in deep water, taken with a dredge; specimens were collected off the Oregon coast, and are either in the Oceanography Department of Oregon State University, or in the personal collection of FP Belcik. Nutting (1909) reported numerous collection points, at stations near San Nicolas Island, and for stations near Point Piños Lighthouse, Monterey Bay. Likely, range extends from southern California to waters off Washington coast. There is the possibility that the species extends further north, to Alaska; further examinations of specimens from that area are in progress.

####### Biology.

An unidentified, anecdotal comment indicated that this form is seen in the assemblage of organisms found at the head of Carmel Submarine Canyon, located offshore at San Jose Creek Beach, near Carmel, California; considered part of a deep-water assemblage that begins to appear at depths between 21–30 m, where turbulence is minimal and fine sediments accumulate on surface irregularities of rock walls. Between 30–61 m, the fauna appears to change very little, suggesting that many of these deep-water forms extend to greater depths.

The neotype designated here (SBMNH 423060, Figure 29) bears on several branches, enlargements that are in actuality gall-like growths, containing epizoic barnacles of the genus *Conopea* (likely *Conopeagaleata*). This is a consistent, common obligate commensal barnacle of gorgonians (Langstroth and Langstroth 2000). On SBMNH 423069 (previously SBMNH 40612), a large cluster of commensal acorn barnacles was seen, on bare-axis portions of the branches. Another specimen, SBMNH 423078, had attached to its bare axis something having, in general appearance, the wooly, cotton-like spittle-bug mass that insects are known to produce on plant stems. Conspicuous brittle stars are intertwined on branches on the specimen from off Newport, Oregon, SBMNH 423073.

####### Remarks.

Cordeiro et al. (2018f) lists *Chromoplexauramarki* as the only species in the genus *Chromoplexaura* in the WoRMS Database.

Kükenthal (1913a) had initially suggested that this species may equal *Psammogorgiaarbuscula* (Nutting, 1909) but later stated that characteristics of this species were completely different. The separation of these two species is reflected in Kükenthal (1924), with separate descriptions for *E.marki* and *P.arbuscula* (syn. *Echinogorgiaarbuscula*). Bayer (1956a), in his description of the two genera in question, *Euplexaura* Verrill, 1869 (colony in one plane) and *Psammogorgia* Verrill, 1868 (colony bushy), indicated some slight overlap.

Kükenthal (1913a; 1924) noted that prior to the discovery of this species, other species in the genus (*Euplexaura*) had only been found in the area of East Asia, from Japan to Singapore and West Australia. It appeared that this was the first red-colored member seen in *Euplexaura* and was the first of the genus from the west coast of North America.

In overall colony shape, some branching occurs; however, more often colony is a single, or rarely branched, stem; any branching from base results in a single or very scarcely branched “whip.” This would have been unique to this eastern Pacific species, along with its obvious, predominantly red sclerites, if it were truly in the genus *Euplexaura* (in most species of the genus, the sclerites are colorless); hence the need for the establishment of the new genus by Williams (2013a). In general colony color and shape, it would be quite easy to simply assume that this organism is *Leptogorgiachilensis*, but an examination of sclerites reveals the distinct differences.

Cairns et al. (2003) had this species listed as a junior synonym of *Leptogorgiacaryi* Verrill, 1868; as noted previously, this is not the case. According to unpublished notes by Bayer, C. (E.) marki might have been synonymous with *Psammogorgiaspauldingi* (now referred to as *Swiftiaspauldingi*). A possible synonymy was considered, with both *Swiftiaspauldingi* (Nutting, 1909), and/or *Swiftiasimplex* (Nutting, 1909). After examinations of multiple samples of what has been labeled as this species and those labeled as *S.spauldingi* or *S.simplex*, if any synonymy were to exist, it would be that between C. (E.) marki and *S.spauldingi.* With the very obvious large, broad spindles, the double-dunce sclerite, I consider this a separate species, but it does exhibit a strong superficial similarity to *S.spauldingi* and there are some shared sclerite forms. An initial conclusion arrived at some years ago (regarding synonymy with *S.spaulding*), seemed to have support with the discovery of a comment made by Bayer (1979). While the statement was an unexpected one to find in this particular article, finding it was noteworthy. It read “The colonies of *A(delogorgia) telones* are similar in general aspect to those of *Euplexauramarki* Kükenkthal (= *Psammogorgiaabuscula* sensu Nutting, not Verrill) and the closely related (if not identical) *Psammogorgiaspauldingi* Nutting, both of which have longer and less sinuous branches.” However, no anthocodial rods in the form of a fingerbiscuit, a key characteristic sclerite of species in the genus *Swiftia* Duchassaing & Michelotti, 1864, have been found in C. (E.) marki specimens, and the initial conclusion was dismissed. A further discussion of California “red whip” diversity follows below and correlations are discussed in Part III of this collection review, on Swiftiacf.spauldingi, but also in the description for *Swiftiasimplex*.

An examination of several specimens (collected by P Etnoyer on a West Coast Survey for NOAA, in the Fall of 2010) was done at Etnoyer’s request. Made available were actual specimens, along with several in situ shots. In digital images, the little-branched colonies were a dirty brick-red or pink (Figure 28); coloring was seen in both extended polyps and throughout branch coenenchyme. An initial diagnosis of the specimens via still images was *Swiftiasimplex*. However, upon physical examination, the polyps themselves were actually white (indicating that only the tentacles were the pinkish color of the coenenchyme) and an examination of the sclerites revealed the large, broad double-dunce spindles so characteristic of C. (E.) marki. If one were to see a colony with little branching, having an overall dirty brick-red to pink color, and did not dissect out a polyp to reveal their white color, or examine the sclerites, an erroneous identification could be made. These specimens presented something of a quandary. Superficially, they looked very much like confirmed *Swiftiasimplex*, yet the sclerites revealed something different. There is a possibility that C. (E.) marki has color variants, with one looking very much like *Swiftiasimplex*. Thus, specimens previously identified in various museum collections as C. (E.) marki may not belong to the genus *Chromoplexaura* at all if double-dunce sclerites are not found, but finger-biscuit rods are.

MBARI has encountered many single or few-branched whips in their investigations. Many of these specimens have been recorded in video and in still photography; a few have actually been collected. A number of principal investigators identify many of these distinct whips as being this species (in genus *Euplexaura*, now *Chromoplexaura*). However, some of those identified as this species may actually be *Swiftiasimplex*; in overall shape very comparable, but in *S.simplex*, the color leans to a dull brick red rather than the usual bright red hue, and polyps of *S.simplex* are always the same color as the coenenchyme, not the “. . . .white, cream or yellow” polyps described for this species by Kükenthal (1913a, 1924), Johnson and Snook (1927) or Williams (2013). Collection of an array of these “whips” when encountered, along with examination of their sclerites and molecular testing of tissue could help to clear up any confusion surrounding these red whip species; in collaboration with E Berntson, M Everett and their colleagues at Northwest Fisheries Science Center (Port Orchard and Seattle, Washington), those needed examinations are currently being conducted.

**Figure 38.**
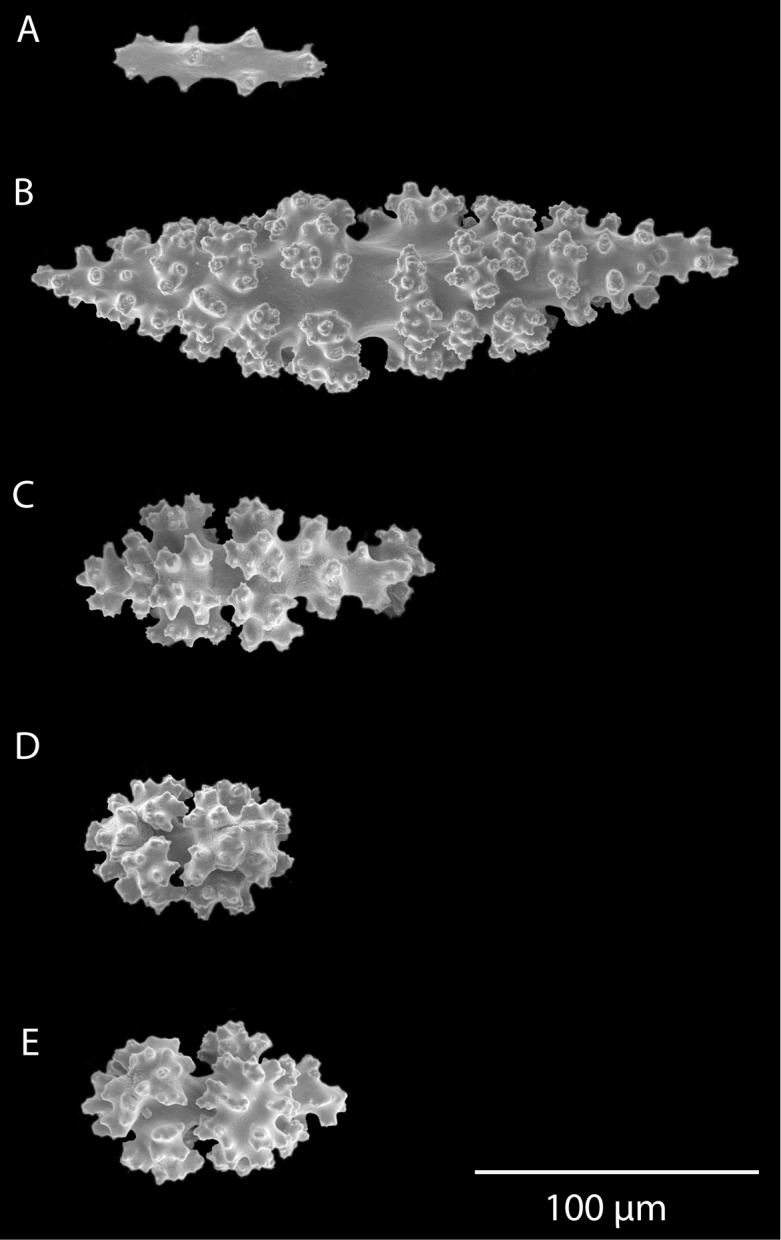
*Chromoplexauramarki*, SEM images, representative forms. SBMNH 265937. **A** Anthocodial form **B** Typical double-dunce-cap **C** Smaller (developing?) double dunce-cap **D–E** Coenenchymal sclerites. Images match variety shown in Williams 2013a (figs 14–17).

##### Discussion concerning diversity of “red whip” forms

**Map A1.**
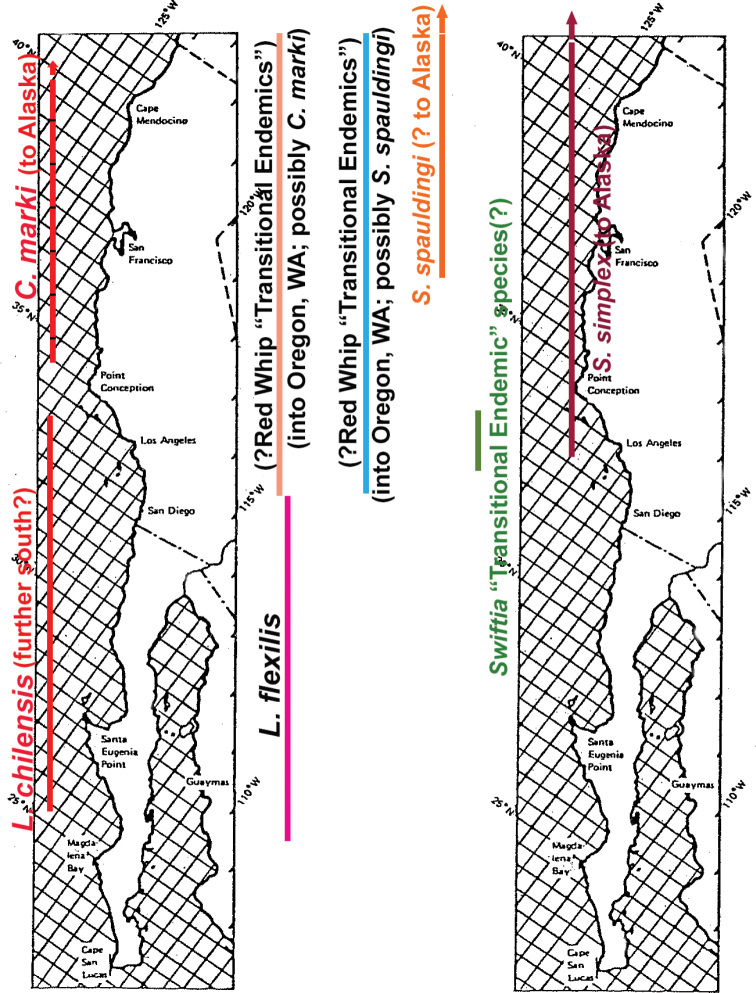
Distribution of gorgonian “red whip” forms. Includes species that are predominantly seen as a whip rather than a branched colony as well as several species that, while usually branched, do frequently display a slender, whip-like body type. Ranges determined from all material examined, with emphasis on specimens housed in SBMNH collection.

From a morphological perspective, with numerous sclerite preparations having been conducted by myself and my research students, it is reasonable to discuss the “red whip” *Chromoplaxauramarki*, and its high degree of variability that could mistakenly lead investigators to name it something else (especially true if colony samples are not taken and/or no sclerite examinations are done). “Red whip” gorgonian species within the California Bight (and in geographic areas immediately adjacent, north- and southwards) present challenges. In the SBMNH collection were found a number of “red whip” specimens, identified by earlier investigators, as the widely common *Leptogorgiachilensis*, but whose sclerites and/or colony form were not that of *L.chilensis*. *L.chilensis* appears as a predominantly thin-branched, but none-the-less, highly branched species, usually seen as a broad fan. Based on location records found for specimens at the NMNH, CAS, etc., it appears that *L.chilensis* is the major “red” species from approximately Central California southward, primarily inhabiting the subtidal zone, often sharing space with *Eugorgiarubens* and both *Muriceacalifornica* Aurivillius, 1931 and *Muriceafruticosa* Verrill, 1868a. (See distribution map for “red whips,” Appendix 2: Map A1 and Appendix 3: Table of characteristics, Table A1. For comparison, distribution of another species of *Leptogorgia*, *L.flexilis*, which could be mistaken for *L.chilensis*, is shown along with distribution range for several of the whip-like species from the genus *Swiftia*.) The other specimens of “red whip” in the SBMNH collection, usually (but not always) appeared as less branched or completely unbranched, slender, but not thin, colonies with very different arrays of sclerites. Locality data indicated their appearance as being far more common in waters of the northern Central California coast, extending northwards, slightly overlapping the range of *L.chilensis*, but usually from waters of greater depth. Potential species would thus be: Chromoplexaura (Euplexaura) marki, *Swiftiasimplex*, or *Swiftiaspauldingi*.

Within this group of predominantly California-collected “red whip” specimens, further subgroupings could be made (based on the appearance of the sclerites): Subgroup 1, “red whip” forms that closely resembled examples of colony variation in Chromoplexaura (E.) marki, but which might be specimens of *Swiftiaspauldingi* and a few indeterminate specimens, looking far more like *S.spauldingi* than anything else. These few specimens are colonies with shorter, chunkier, more heavily warted spindles, along the lines of those from the specimen at NMNH (USNM 78385), labeled as *Swiftiaspauldingi*, collected in Monterey Bay. Further collection efforts are required, particularly in the transitional area of California’s Point Conception, so as to document extent of occurrence in and near the California Bight. The notion of cryptic species or transitional endemics may apply here, not an unusual occurrence in a region like the California Bight; an equally likely possibility may be that of hybridization between certain species.

Other remaining small, odd whip-like colonies (in five separate lots), SBMNH 265946, 265947, 265948, and 265949, comprising Subgroup 2, bore a stronger superficial resemblance to a specimen collected by J Ljubenkov (which he misidentified as *Muricellacomplanata* Wright & Studer, 1889, and as yet, are still not confirmed as to their identification). These are the most difficult to link with a known genus or species. Most of these very small fragments are a bright, vivid red (or reddish pink), and have noticeably white polyps. At this point, they most closely fit very small specimen examples of the morphological variety seen in *C.marki*. But, the sclerites generally did not closely match those seen in known “red whip” species. There are few to none of the double dunce-caps of *C.marki*, the simple spindles of *L.chilensis*, or the spindles and rods that can typically be seen in any species from the genus *Swiftia* (however, *Swiftiakofoidi* (Nutting, 1909) does not always display the definitive rod-shaped sclerite). Unfortunately, compounding the problem is the overall condition of the specimens; they are very small, without much coenenchymal tissue. There is now so little material to work with that without collecting more material, continued work with these specimens would destroy what is left; none of these are identified to my satisfaction. Without more material, collected in the same areas as these lots, I cannot proceed further with a conclusive identification (see Appendix 1: List of material examined.)

Two additional lots (SBMNH 423066 and 423067) presented with sclerites somewhat intermediate between that seen in those labeled as *L.chilensis* and the above mentioned subgroups. Location data indicated the possibility of them being examples of *L.chilensis*, but sclerites did not fit as cleanly as would have been expected. I considered the possibility of these specimens being part of an array of cryptic species (within one or more of these genera and species) or that these gorgonians are either transitional endemic or hybridized forms. Currently, these are listed as material examined labeled *L.chilensis*, but further examinations may alter that placement. Based on location records, each of these several specimens may demonstrate a need for establishing different colony types as different subspecies (cryptic species?), but could equally show degree of overlap (and possible morphological transitions) of various known species. An attempt has been made to show key characteristics of each (refer to Appendix 3: Table A1). Overlap of these gorgonians confirmed my understanding of the California Bight as being an area of tremendous diversity (and as a result, an area where confusion regarding very similar-looking forms can occur), an area understood ecologically as an environmental disjunction. This overlap supports the admonition to anyone working in this geographical area, encountering these gorgonians, to collect and examine multiple specimens and to do extensive sclerite preparations in those comparisons. Further collection efforts will be helpful in documenting the extent of occurrence of each of these species, not only in the California Bight, but in areas adjacent to it, both north and south, with final determination likely coming from molecular comparison studies, which could confirm such concepts as cryptic species or regional (Point Conception) transitional endemics. Somewhat fortuitously, an interesting and pertinent statement was discovered in the introduction to the first volume of “New Zealand Inventory of Biodiversity: Volume I: Kingdom Animalia,” edited by Dennis P Gordon (2009). To quote: “Often, what we think is a single species turns out to be a complex of closely related cryptic (hidden) species that resemble each other so closely their existence had been overlooked.” This may well be the situation for “odd red whips” and several species of *Swiftia* (along with other genera) in the eastern Pacific. Appendix 2: Map A1 shows the distributional ranges and geographical overlap of known species and possible endemic intermediates.

Pertinent specimens at CAS were examined. For a substantial gorgonian collection of eastern North Pacific Ocean species, CAS has taken the correct stance on “red whips” from California and parts north; twenty four out of twenty five lots (identified as *Euplexauramarki*, now *Chromoplexauramarki*) are from Morro Bay and areas north, with most from Monterey Bay and Oregon. According to the database, they do not have specimens of either *Swiftiasimplex* or *Swiftiaspauldingi*; three specimens are listed as *Euplexaurasimplex* (genus name usage not seen elsewhere, erroneously proposed by Harden). Material from several other sources (NOAA, NMFS, MBARI, etc.) were examined to broaden the scope of my understanding of eastern Pacific “red whips”. There was little doubt that “red whips” seen by MBARI would likely not be L.chilensis. That would be the case for two reasons: depth at which colonies are seen, and location where MBARI researchers are doing their work (well north of the California Bight). A dry specimen of Chromoplexaura (Euplexaura) marki collected in Monterey, California, housed in the Smithsonian collection (USNM 51500), was of interest. It displayed sclerites very different from what would be seen in *L.chilensis*, but also did not clearly display the key sclerite form for the genus *Chromoplexaura*, the large double dunce-cap sclerite. Of further interest was the fact that NMNH listed only three specimens of *Leptogorgiachilensis*, all from La Jolla, San Diego or southern California, but had two lots of *Lophogorgiachilensis*, taken off the Washington coast! These were collected in 2006 at depths of 84 and 232 meters, on that year’s NOAA “Deep Sea Coral and Sponge Habitat Expedition.” These latter two are more likely either *Swiftiaspauldingi* or one of the other species mentioned above.

##### Genus Muricea

Taxon classificationAnimaliaAlcyonaceaPlexauridae

Lamouroux, 1821

Muricea (pars) Lamouroux, 1821: 36. (pars) Blainville 1834: 509. (pars) Ehrenberg 1834: 134. Dana 1846: 673. (pars) Milne Edwards 1857: 142. Duchassaing and Michelotti 1864: 14. (pars) Kölliker 1865: 135. (pars) Verrill 1868b: 418–419; 1868c: 411; 1869a: 449–450. Kent 1870: 84, pl 41, figs 13–17, (?) 36–37. (pars) Studer 1878–1879: 649. Studer 1887: 58. Wright and Studer 1889: 93, 133 + pl. Gorzawsky 1908: 8. Nutting 1910a: 9. Kükenthal 1919: 752, 835; 1924: 141. (pars) Riess 1929: 383–384. Aurivillius 1931: 102–103. Deichmann 1936: 99. Bayer 1956a: F210; 1959a: 12; 1961: 179–180. Tixier-Durivault 1969; 1970a, b, c: 154. Bayer 1981c: 930 [in key only]; 1994: 23–24. Marques and Castro 1995: 162. Hardee and Wicksten 1996: 127–128. Castro et al. 2010: 779. Breedy and Guzmán 2015: 6–7; 2016b: 7–8.
Emuricea (pars) Verrill, 1869a: 449. Studer 1887: 58. Wright and Studer 1889: pl LVI. Nutting 1909: 718. Thomson and Simpson 1909: 258. Kükenthal 1919: 836. Reiss 1919: 397–398. Kükenthal 1924: 149–150. Thomson 1927: 48–49. Reiss 1929: 397. Aurivillius 1931: 50 (emended). Deichmann 1936: 104.
Eumuricea (Muricea) Bayer, 1981: 930 (key). Breedy and Guzman 2015: abstract, 28.

###### Type species.

*Muriceaspicifera* Lamouroux, 1821, by subsequent designation Milne Edwards and Haime 1850: lxxx. Kükenthal (1924) listed *Muriceamuricata* (Pallas, 1766) as the type. [*M.specifera* was later synonymized with *Muriceamuricata* Pallas, 1766 *apud* Bayer, 1961: 179–180]

###### Diagnosis.

Arborescent colonies richly branched (dichotomously or laterally), often in one plane. Branch diameter moderate to very thick, tendency to curve upwards, most nearly parallel to one another, tips tending to slightly swollen. Calyces shelf-like, on all sides, close-set, prickly, tubular or distinctly projecting (at right angle or upwards); stiffened by large, fusiform sclerites; aperture wide and eight-rayed; polyps fully retractile. Axis purely horny; weakly loculated (if at all). Sclerites usually fusiform, long, often massive, spindles (up to 3.0 mm in length), obviously sculptured, with strong outer or terminal spines, or both, arranged in calycular wall longitudinally; rarely some irregular forms. Anthocodial sclerites numerous, small spindles, forming at most weak, slightly differentiated transverse crown or collaret below tentacles, converging on bases of tentacles. Sclerites in genus stated as generally, markedly stockier, denser and thicker; a bit larger overall, than those seen in many other genera.

###### Remarks.

The genus *Muricea* may contain at least a dozen species specifically found in the eastern Pacific; however, species descriptions, and their potential synonymies, needed review. The work of Breedy and Guzmán (2015, 2016a, 2016b) has been of help. But, the California Bight is a complex area in the eastern Pacific, and may hold some surprises with regards to this genus. To date two, perhaps three, species are commonly recorded from the California Bight; others, however, occasionally may appear. A more extensive and thorough discussion of this genus as it appears specifically in the California Bight may be required. A further investigation is also necessitated by the fact that the number of species of Mexican *Muricea*, in both the SBMNH collection and other collections, is far greater than the number of species currently known to occur within the California Bight. While the review of the genus *Muricea* and its species by Breedy and Guzmán (2015, 2016a, 2106b) is helpful, further investigation of *Muricea* found in the eastern Pacific waters of California and upper Baja, Mexico is still required; specimens from the SBMNH and LACoMNH will be helpful in such investigations.

##### Muricea californica

Taxon classificationAnimaliaAlcyonaceaPlexauridae

Aurivillius, 1931

**Figure 39.**
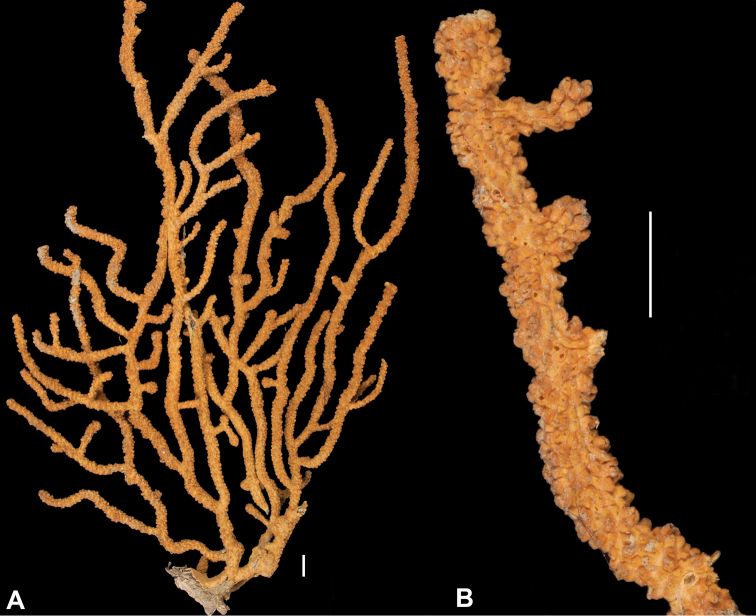
*Muriceacalifornica*, SBMNH 422921. **A** Whole colony, 25.5 cm × 15 cm **B** View of branch tip magnified to show prominent, somewhat rounded calyces.

**Figure 40.**
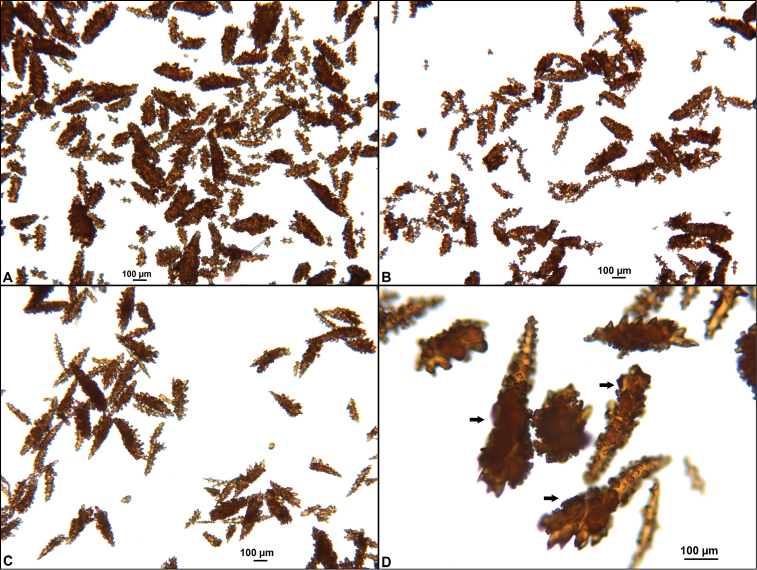
*Muriceacalifornica*. **A–C**SBMNH 422911, SBMNH 422914, and SBMNH 422361, respectively; light microscopy arrays, 4× magnification, showing diverse mix of sclerites seen in the species **D** Light microscopy array, the “torch-like,” multi-toothed sclerites so typical of *M.californica*, shown at 10× magnification. In **D** the middle, upside-down torch measures some 580 µm, the middle upright torch measures ~400 µm, while the torch lower down measures 480 µm.

**Figure 41.**
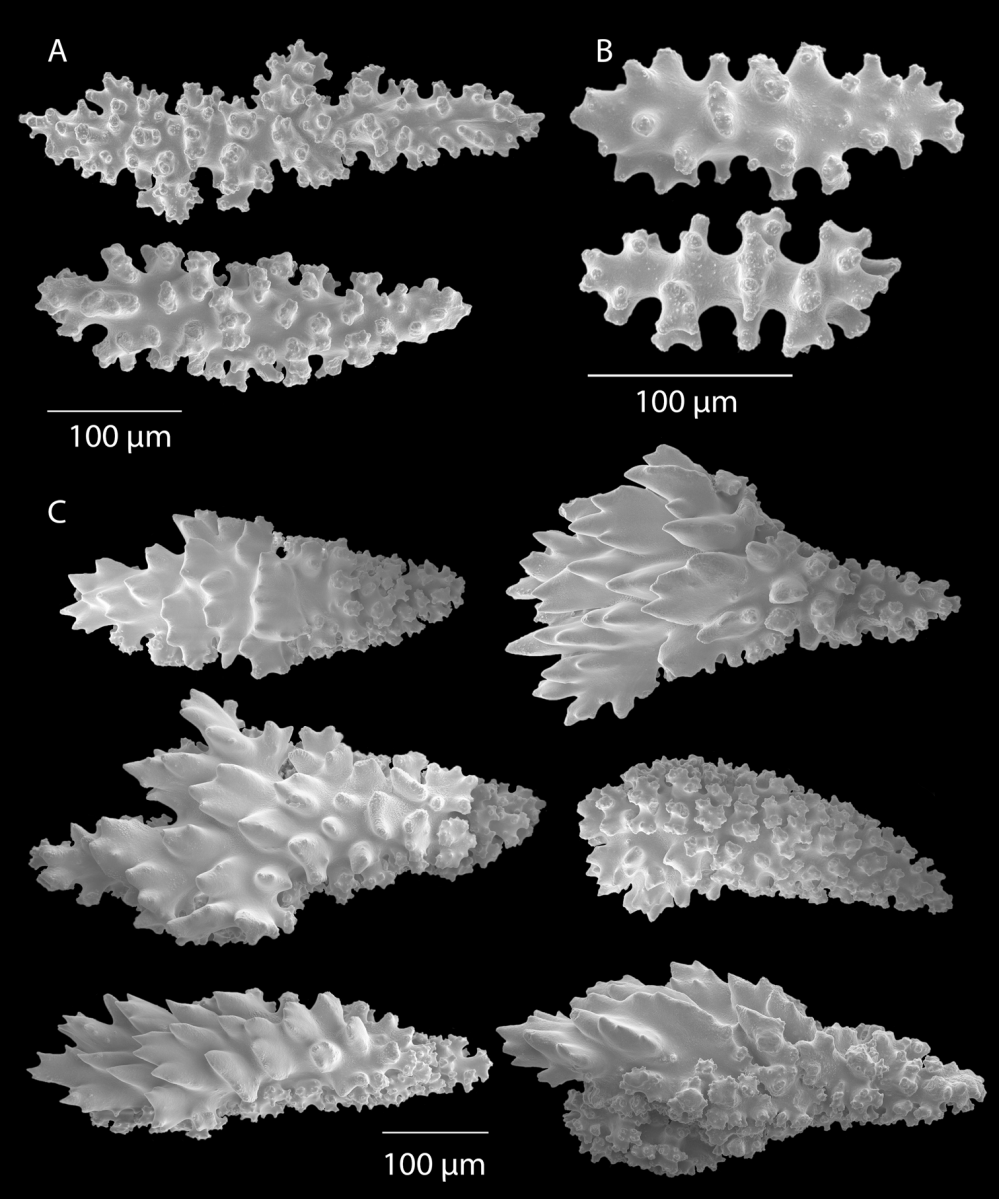
*Muriceacalifornica*, SBMNH 265938, SEM image. **A, B** Axial sheath sclerites **A** Very jagged spindles **B** Moderately “toothed” spindles **C** Calycular/coenenchymal torch-type spindles. Compare/contrast with those shown in Breedy and Guzmán 2016 (fig. 21).

Figures 39A, B , 40A–D , 41A–C

? Gorgoniaplantaginea Valenciennes, 1846: pl 15 (non Lamarck).
? Muriceaappressa Verrill, 1864: 37; 1866b: 329; 1869a: 44. Grigg 1970: xiv, 20, 25, 207; 1977: 280.)
? Muriceaappressaflavescens Verrill, 1868a; 1869a: 446 (? nec Verrill, 1864: 37).
Muricea californica Aurivillius, 1931: 111–114, fig, p 113. Hardee and Wicksten 1996: 130–132. Breedy and Guzmán 2016b: 32–34.

###### Type locality.

North Pacific Ocean, California Channel Islands, Santa Catalina Island, Gulf of Santa Catalina, 2–27 m.

###### Type specimens.

**Syntype**USNM 44188 [wet]; **Lectotype** USA: Swedish Museum of Natural History 1122 [wet]. Syntype specimen was examined; a common form in California waters, often easily identified.

###### Material examined.

~32 lots (see Appendix 1: List of material examined).

###### Description.

*Colony* (Figure 39A) non-reticulate; up to 100 cm wide, 120 cm high, usually 60 cm or less. Loose, dichotomous, irregular branching primarily in one plane, forming heavy fan-shaped colony; some primary and secondary branches extend out of plane. Branches thick, averaging 2.0–5.0 mm in width; curve to lie parallel with main branch. Branching lateral, terminals of even thickness or tapering slightly. Outer coenenchyme mostly occluded by calyces (Figure 39B). Calyces distally open cups, erect, very elongated, prominent, conical in shape, 0.8–1.1 mm tall, 1.0–1.5 mm across, 1.0–2.0 mm apart (close together, but not overlapping), protruding 45 to 90 degrees away from branch when polyp extended (extendable to 3.0 mm). When polyp not extended, calyx lying close to and curving into stem, broad and smooth (like bracts in a partially closed pine cone). Upper lip varies, from those without sclerites to having definite lip. Calyces extend in all directions around branches. Tentacles taper at tip; bear two rows of lateral pinnules that are slightly displaced to the oral side. Color of living colony generally rusty brown; ranges from golden-brown to dark reddish orange to reddish brown to brown to dark brown. Axis reddish brown at base; becomes light yellow-brown at tips. Polyps most commonly yellow; golden orange, bright yellow, pale yellow, creamy yellow, even white; all polyps of a branch the same color. Possibility that more than one color of polyp per colony occurs (demonstrated in digital images sent to me by Mary Wicksten, and examination of specimens). Dry colonies dark rusty brown. Sclerites (Figure 40A–D) rust red to golden-brown. Sclerites predominantly club-shaped with large, rounded spines or pointed tubercles projecting from broader, club-shaped end; other end tapering, covered with tubercles (Figure 41C). Outer coenenchyme consisting of small sclerites, to 0.5 mm long, torch-like, with processes often continuing irregularly down one side, other end tapering, covered with tubercles. Some spindles fusiform, bent very slightly in middle, or having large processes in middle projecting outward. Inner coenenchyme spindles small, fusiform, set with distinct tubercles.

**Figure 42.**
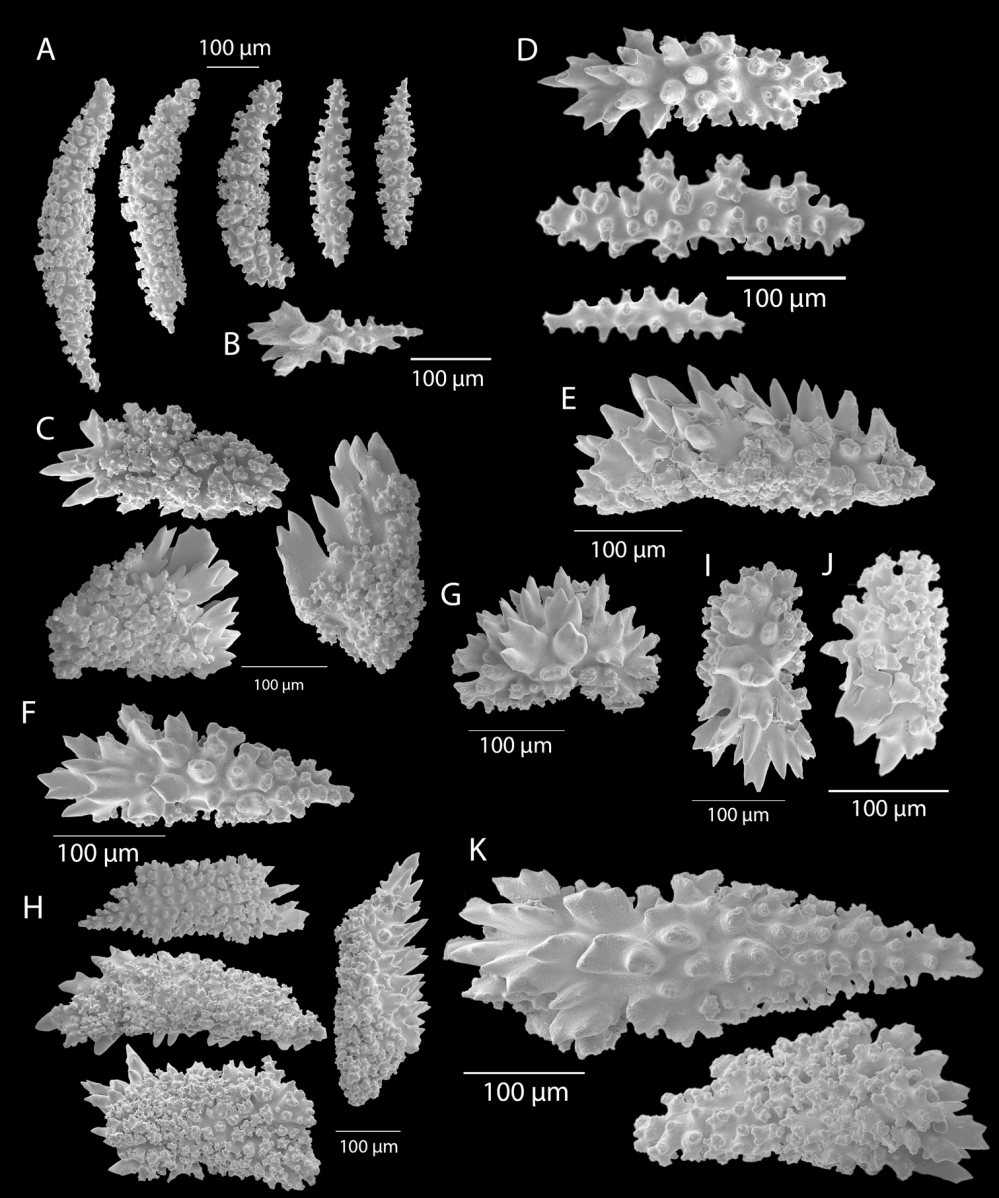
*Muriceaplantaginea* (= *Muriceaappressa*), SBMNH 422909 (Ecuador) and SBMNH 422417 (Baja CA Sur). Image included for comparison between sclerites found in *M.californica* and those seen in *M.plataginea* [= *M.appressa* Breedy & Guzmán, 2016]. SEM image. **A–F**SBMNH 422909 (**A–C**); **E–F** Calycular/coenenchymal sclerites **A** Elongated, jagged spindles **B** Small torch-type **C** Rounded, stout torches **D** Shorter, jagged spindles (axial sheath) **E** Unilateral spinous spindle **F** A second unilateral spinous form tending to torch shape **G–K**SBMNH 422417 Calycular and coenenchymal sclerites **G** Fan-shaped sclerite **H** Large, truncated unilateral spinous spindles **I** Stout prickly spindle **J** Rounded, unilateral spinous type **K** Torch-types typical of *M.californica*. Compare sclerites **A–F** with those shown in Breedy and Guzmán 2016 (fig. 15, for *M.plantaginea*) and sclerites **G–K** with those shown in Breedy and Guzmán 2016 (fig. 21, for *M.californica*).

Sclerites examined compared to those shown by Hardee and Wicksten (1996); there can be dense coverings of warts, but condition not seen on all sclerites; some sclerites have dense covering of warts at one end, but not at other end. On largest sclerites, warts are large, very few a bit bigger than those shown by Hardee and Wicksten (1996) for *M.fruticosa*. Many more of largest sclerites have flame-like teeth at one end, not down entire length of one side. In drawings and photographs examined of those believed to be, or labeled, this species, much larger tubercle bumps are seen. Regarding flame-like teeth, on some sclerites there are scattered, randomly-placed spines running down the side, but many more have flame-like teeth only coming off one end (reminiscent of a flaming torch; Figure 41C). Surface bumps can be dense, but not always, not over whole surface (true of some). For this species, stocky, dense clubs and torches very evident; overall, sclerites give impression of being a bit larger and more densely warted than those that may be from *Muriceaappressa*. Refer to Figure 42, shown as *Muriceaplantaginea* (Valenciennes, 1846); largest sclerites, however, not nearly as large as those seen in *M.fruticosa*.

###### Etymology.

Surmise that the name *californica* refers to the species type locality; no explanation for the species derivation was found.

###### Common name.

California golden gorgonian; California’s purple one; California rust gorgonian; Brown sea fan (so called in a variety of field/diving guides).

###### Distribution.

Stated as ranging from Point Conception, through Baja (Santa Maria) to the Gulf of California; range extending further south is possible. Grigg (1977) remarked that if *M.californica* is identical to *M.appressa* (See Harden 1969; Grigg 1970 for rationale), southern limit for this species is then Zorritos, Peru. From the following list of California sites, running from south to north, note depth ranges indicated. On the **Mainland**: Point Loma: 5–18 m; La Jolla: 3–27 m (USNM 50190, was collected at 11 m, in La Jolla, at the Torrey Pines kelp bed, 5 miles north of Scripps Institution); USNM 77286 was collected at Corona Del Mar, Newport Bay; Newport Beach: 2–12 m; San Pedro: 3–18 m; yet another at NMNH (USNM 52485) was collected along the southern coast. **Islands**: Coronados Islands: 5–24 m; Santa Catalina Island: 5–30 m.

###### Biology.

Common in kelp beds (Ricketts, 4^th^ Ed. 1968); found at depths greater than 3 m, perhaps being one of the most common gorgonians from southern California (Hardee and Wicksten 1996). Grigg (1977) noted it as a species seen in the rocky sublittoral zone off California at depths between one and 30 meters.

As colonies grow, they form annual growth rings in the skeleton (Grigg 1974; Bertsch 1984). Colonies grow separately sexed, requiring 5–10 years to reach sexual maturity with a maximum longevity of ca. 50 years (Grigg 1975). Grigg (1977) indicated that mortality could be caused by abrasion from suspended particulate matter when there are high waves, smothering by sand and by encrustation due to presence of zoanthid cnidarians *Savalialucifica* (Cutress & Pequenat, 1960) and *Epizoanthusinduratum* Cutress & Pequenat, 1960. On specimens of *Muricea* (potentially this species) located on Shale Reef, between Corona del Mar and Laguna Beach, *Savalialucifica* was found (a wet SBMNH specimen, as yet uncataloged). As well, SBMNH 422359 has a heavy growth of some form of epizoanthid. Presence of the colonial anemone *Corynactiscalifornica* (Carlgren, 1936) may also be an important source of mortality.

Satterlie and Case (1978) used this species extensively in studies on the neurobiology of gorgonian coelenterates, which they obtained locally (Santa Barbara area, at depths of 4–11 m). In a study done by Lissner and Dorsey (1986), it was noted that while this species was common around the Channel Islands and rocky areas of the mainland, it was conspicuously absent on the Tanner and Cortes Banks, and on the Santa Rosa-Cortes Ridge. In an anecdotal notation made by R Grigg, the reason could be that populations of *Muricea* species may be limited by cold water and/or poor dispersal abilities of the larvae. Grigg (1977) stated that *Muricea* species rarely cover more than 1% of the sea bed, where space is fully occupied.

Considering associations this species has with other organisms, Humes and Lewbel (1977) reported two species of *Acanthomolgus* Humes & Stock, 1972 (cyclopoid copepods) for the first time from the eastern Pacific in association with this gorgonian, from an area near La Jolla, California (Quast Reef). They indicated that these copepods are consistent members of the epifaunal community on the gorgonian, found with the gorgonian throughout the year. In association with SIO/BIC #CO 1600, there appeared to be the exoskeletal remains of either skeleton shrimp, or some other small, (and now pale) crustacean. Notes from H Bertsch (1984) indicated that sometimes the ovulid snail Simnia (Neosimnia) vidleri (GB Sowerby III, 1881) could be found eating this gorgonian; Grigg (1974) reported that the only fish known to feed on *Muricea* species is the Garibaldi *Hypsypopsrubicunda* Girard, 1854 (from Clarke 1970). A specimen, from Baja, South Bay, Isla Cedros (SBMNH 422363), had very well developed acorn barnacle galls, completely overgrown and covered by healthy-looking coenenchyme; only the barnacles’ uppermost valves were visible. They appeared to have been well protected and secured to their gorgonian host. One additional specimen (SBMNH 422362) displays a bare axis at the tip of several branches; obvious barnacles are attached to these bare-tip sites. Overall, extensive organismal growth is uncommon on *Muricea* specimens, both wet and dry, housed in the SBMNH collection.

###### Remarks.

Conflicting comments about this species (comparing it with *Muriceafruticosa* Verrill, 1868a) have been made, particularly with regards to polyp color (Ricketts 3^rd^ Ed, 1962; Ricketts et al. 5^th^ Ed, 1985; Harden MS thesis 1969; unpublished pencil notation, Harden; Harden PhD dissertation 1979). In reading these it was clear to me that some identified the species by presence of yellow polyps, while others did so by white ones. There is no doubt that discrepancies regarding polyp color have carried into current identification of the species. It would be well to remember that color is hard to determine in underwater situations, in ambient light conditions, or with artificial light sources. In some of the oldest descriptions, polyp color was not always clearly stated, most likely due to collection procedures and gaps of time that ensued between collection and actual examination. There is evidence, based on my own examinations, that *M.californica* can have yellow or white polyps (even different colored polyps in different locations within the same colony). Confirmation came via e-mail correspondence with M Wicksten, and is stated in Hardee and Wicksten (1996: 129, 138). Divers comment that often colonies closely situated side-by-side, with basically the same colony form (thickly branched, usually in one plane), with the distinctively common brown coenenchyme, can display markedly yellow polyps in one colony and in the other (closely adjacent) one, obvious white ones. Because the colony form is so similar (perhaps due solely to current flow in the immediate area, thus an environmental condition), but polyp color so different, the two are considered (and identified) as being different species, that with yellow polyps, *M.californica* and those with white polyps, *M.fruticosa*. Yet, there is the question as to whether polyp color can be an accurate means of identification in situ, especially under low light conditions, or if color variation is genetically inherent. Questions that still require further study: 1) are colonies (yellow polyps vs. white polyps) two distinct species? 2) If so, are the two species so similar that they can hybridize? Or, 3) do two color morphs of this species exist (in southern California, at least, each with the characteristic large, planar, generally dichotomously branched colony shape)? Only an examination of sclerites, with notation made of polyp color at the time of collection, could clearly answer these questions. Channel Islands National Park, in annual fish surveys, identifies *M.fruticosa* as that with white polyps, while *M.californica* has yellow polyps. The Aquarium of the Pacific in Long Beach (via phone communication with P Clarkson, March 2003) identifies them in the same way. Unfortunately, most specimens originally identified as this species in the SBMNH collection had either sat in formalin since initial collection, had been in alcohol quite a long time, or are extremely dry; there is no color to be seen in the polyps (even if at one time they had color) and data labels usually do not indicate polyp color at time of collection. Fresh material has been requested from local sources, as they incidentally collect, to help clarify identification of this, and very similar looking species.

Allen (1976) stated that *M.californica* had been common at Corona del Mar in the past, and D Kushner from Channel Islands National Park (phone comm., March 2003) stated that there has been an enhanced abundance of *M.californica* observed in recent years at San Onofre, almost to the point of taking over, becoming quite abundant in the area. This is an area where I would expect to find this species, as it appears to be common in southern California, extending into Baja, but this growth spurt seemed a somewhat unusual event. The small collection of dry specimens (and of living specimens on public display) at the San Pedro Cabrillo Marine Aquarium holds many colonies identified as this species, collected in the surrounding local area and off the southern California Channel Islands.

Coloring of actual sclerites was often of little help, but observation of sclerite size and shape was crucial. Some of the specimens identified originally as *M.californica* (and for that matter, some identified as *M.appressa*) were actually the typical variant of *M.fruticosa*, but because of condition of colony, method of preservation and transitional sclerite forms, it was initially difficult to clearly separate the species; calyx appearance was used as well, but again, groupings into distinct clusters was not always easy/clear. There was inclination to think that some specimens, listed as *M.californica* (likely because of overall colony shape), but from far more southern (Mexican or even Central American) collection locations, might actually be *M.appressa* or *M.fruticosa*. Without fresh material (having specific data location, observation of polyp color recorded, along with more extensive and careful extraction and preparation of the large and jagged sclerites, so as not to break them or break off the teeth), can these *Muricea* specimens be confidently assigned a species name. As *Muriceacalifornica* seems the most common form, those listed in the Appendix 1 (List of material examined) are listed as *M.californica*, unless it was very clear, based on calyx appearance and sclerite form/size, that they were another species. Examination of recent, locally collected colonies (SBMNH Sea Center, Aquarium of the Pacific, Long Beach and OCSD), while not numerous in quantity or large in size, was invaluable. Sclerite size and shape were noted, along with calyx shape and confirmed polyp color. It would appear that *M.californica* has a wide range of polyp coloring (rarely white to commonly yellow or rich gold), while its sclerites are distinct in not having the large, densely warted, rounded spindles common to *M.fruticosa* (which only displays white polyps). In situ identification, therefore, can be challenging if the colony in question has white polyps; both *M.californica* (occasionally) and *M.fruticosa* (typically) have white polyps, while *M.appressa* (as *M.plantaginea*, included briefly below, for comparison) seems to always display yellow polyps, (which is normal for *M.californica*). Despite the challenges regarding in situ identification, *M.californica* (as well as *M.fruticosa* and *M.appressa* (= *M.plantaginea*) are all recognized as separate species in the WoRMS Database (Cordeiro et al. 2018g).

##### Muricea plantaginea

Taxon classificationAnimaliaAlcyonaceaPlexauridae

(Valenciennes, 1846)

Figure 42A–K

Gorgonia plantaginea Valenciennes, 1846: pl 15.
nec Gorgoniaplantaginea Lamarck, 1815: 163.
nec Euniceaplantaginea Valenciennes, 1855: 13; Milne Edwards and Haime 1857: 146, 151.
Eunicea tabogenesis Duchassaing & Michelotti, 1860: 17; 1864 [1866]: 111. Kükenthal 1924: 145.
Eunicea ransoni Stiasny, 1937: 331, 334–336, figs 5, 6, 7.
? Muriceaappressa Verrill, 1864: 37 [January]; 1866: 329; 1868a: 412; 1869a: 444–446; pl VIII, fig. 13. Kükenthal 1919: 752; 1924: 145. Reiss 1929: 390–391. Hardee and Wicksten 1996: 132–136 (syn. n).
Muricea appressa var. flavescens Verrill, 1869a: 446. Kükenthal 1919: 752; Kükenthal 1924: 145 (syn. nov.). Hickson 1928: 371–372. Reiss 1929: 389–390. Stiasny 1943: 72–74.
Muricea plantaginea Lamarck, 1836: 333. Breedy and Guzmán 2016b: 25–32.
Muricea californica Aurivillius, 1931: 111–114 [according to Grigg, 1977: 280, after Grigg, 1970: xiv, 20, 25, 207].
Muricea tenella Verrill, 1869a: 446–448. Kükenthal 1919: 752; 1924: 145. Hickson 1928: 371–372. Reiss 1929: 389–390. Stiasny 1943: 72–74.

###### Type locality.

**Holotype** Mazatlán, Mexico, Voyage sur la Frégate La Vénus, MA Du Petit Thouars, 1836–1839. Also, Peru, Tumbes Department, Zorritos, 3–5 fm [6–9 m]. Specimen from NMNH (USNM 33585, and many others) collected in the North Pacific, Panama.

###### Type specimens.

**Syntype** YPM 1616A of Muriceaappressavar.flavescens. As well, housed at NMNH, USNM 33585, listed as a **Syntype**, with SEM image #2517; [dry]. Syntype specimen at NMNH was examined.

###### Material examined.

A number of lots housed in SBMNH collection (see Appendix 1: List of material examined).

###### Remarks.

Included here is a brief commentary on this species, and an SEM plate (Figure 42A–K) is provided as a means of comparison, because as Grigg (1977) stated “this could be synonymous with *M.californica*.” However, the work of Hardee and Wicksten (1996) led them to conclude that *M appressa* (= *M.plantaginea*) is not synonymous with *M.californica*. Based on my own observations and research, *M.appressa* is more likely to be found south of the California Bight (Baja, CA Sur, Ecuador, Galápagos, etc.). Johnson and Snook (1927) made mention of storm-washed living specimens of *Eunicea* Lamouroux, 1816 (but no reference to a species) with the living polyps yellowish white (the black and white photograph of a specimen shown in that volume looked most like a somewhat worn specimen of either *M.californica*, or perhaps *M.fruticosa* typical). While those identified as *M.appressa* in the SBMNH collection generally seemed more prickly in overall appearance (as compared to *M.californica*), along with slightly smaller-diameter branches, any cursory visual inspection of gorgonian specimens from this genus could misidentify species. A more intentional study of calyx shape along with further comparisons of sclerites from freshly collected specimens over the total range of the Bight to clarify the possible synonymy of this species with *Muriceacalifornica* is underway. I am inclined to keep *Muriceaappressa* (= *M.plantaginea*) a separate species while this further study is being conducted.

Several additional locations were noted for this species in Verrill’s (1864; 1869a) description: Panamá and the Pearl Islands, in pools at extreme low water; ex. FH Bradley; also, JH Sternbergh and FH Bradley. Also, records from Nicaragua, Corinto; ex. JA McNiel and from Mexico, La Paz; ex. J Pedersen. Note that all of these locations lie well south of the California Bight’s southern boundary.

Compounding the confusion surrounding *Muricea* species, particularly in the southern portion of California’s geographical range, is that in the following description of *Muriceafruticosa*, two very distinct colony forms must be mentioned: that which looks very much like the typical *Muriceacalifornica* (the typical colony shape, albeit with white polyps, according to most encountering it in the field) and that with a far smaller, stiffer, shorter-branched cespitose or fruticose bushy shape, a distinctly different variant of *M.fruticosa* according to Verrill (1868a; 1869a). As this latter variant is not encountered in the southern California Bight it is not discussed here.

##### Muricea fruticosa

Taxon classificationAnimaliaAlcyonaceaPlexauridae

Verrill, 1868

**Figure 43.**
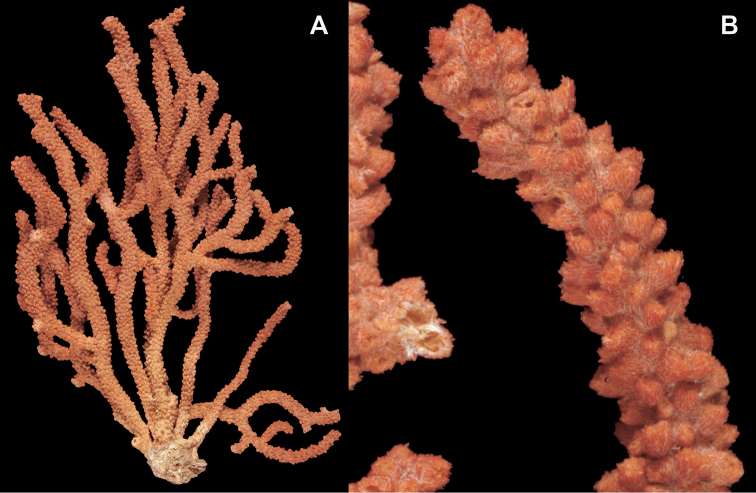
*Muriceafruticosa*, SBMNH 422430 (as seen in California waters). Colony shows pale base area tending to darker, colored branch tips. **A** Whole colony, 15 cm tall × 11.0–11.5 cm broad **B** Closer view of branch tip showing conical calyces forming shelf-like projections off branch surface.

**Figure 44.**
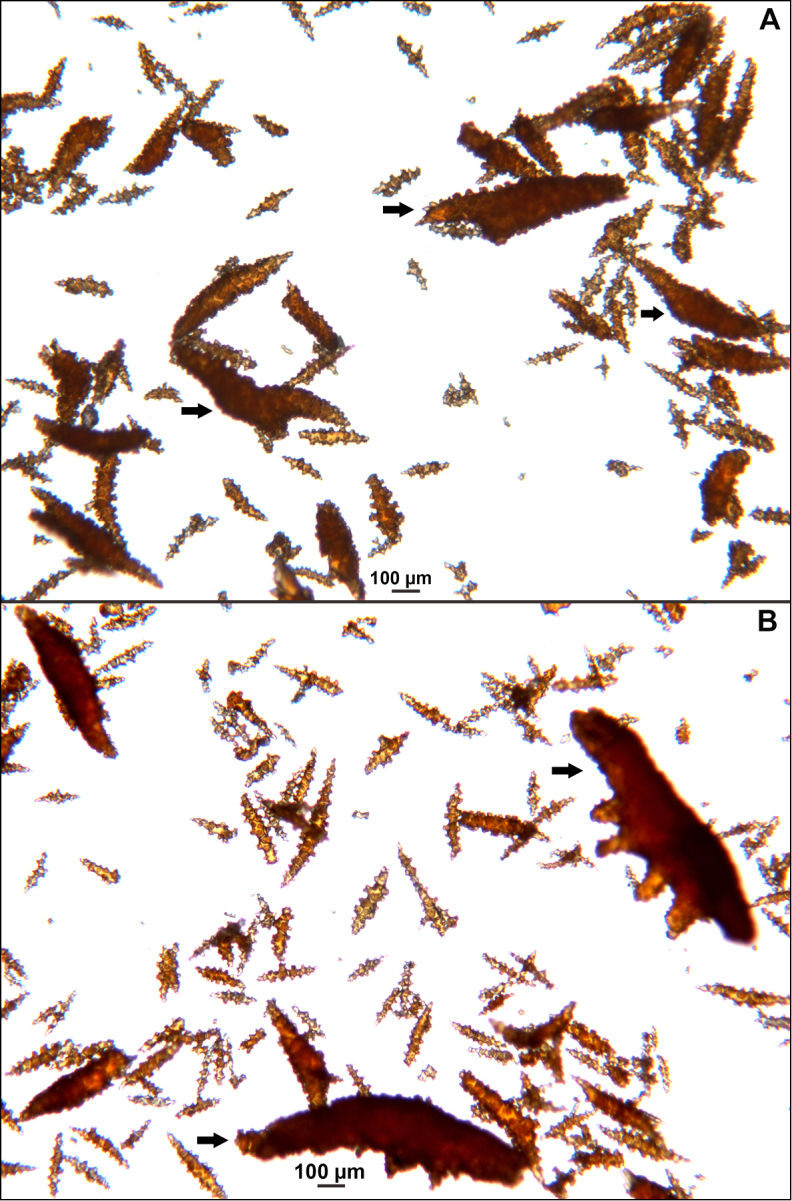
*Muriceafruticosa*, SBMNH 265940, light microscopy arrays. **A, B** 4× magnification, with diverse sclerites shown for the species. Of particular interest are prominent sclerites, indicated by arrows, in **B** (the tardigrade-like form mentioned in text description). In **B** the lower, middle sclerite measures some 1300 µm in length, the one up and to the right of that measures 1133 µm; thinner spindles measure between 200–300 µm in length.

**Figure 45.**
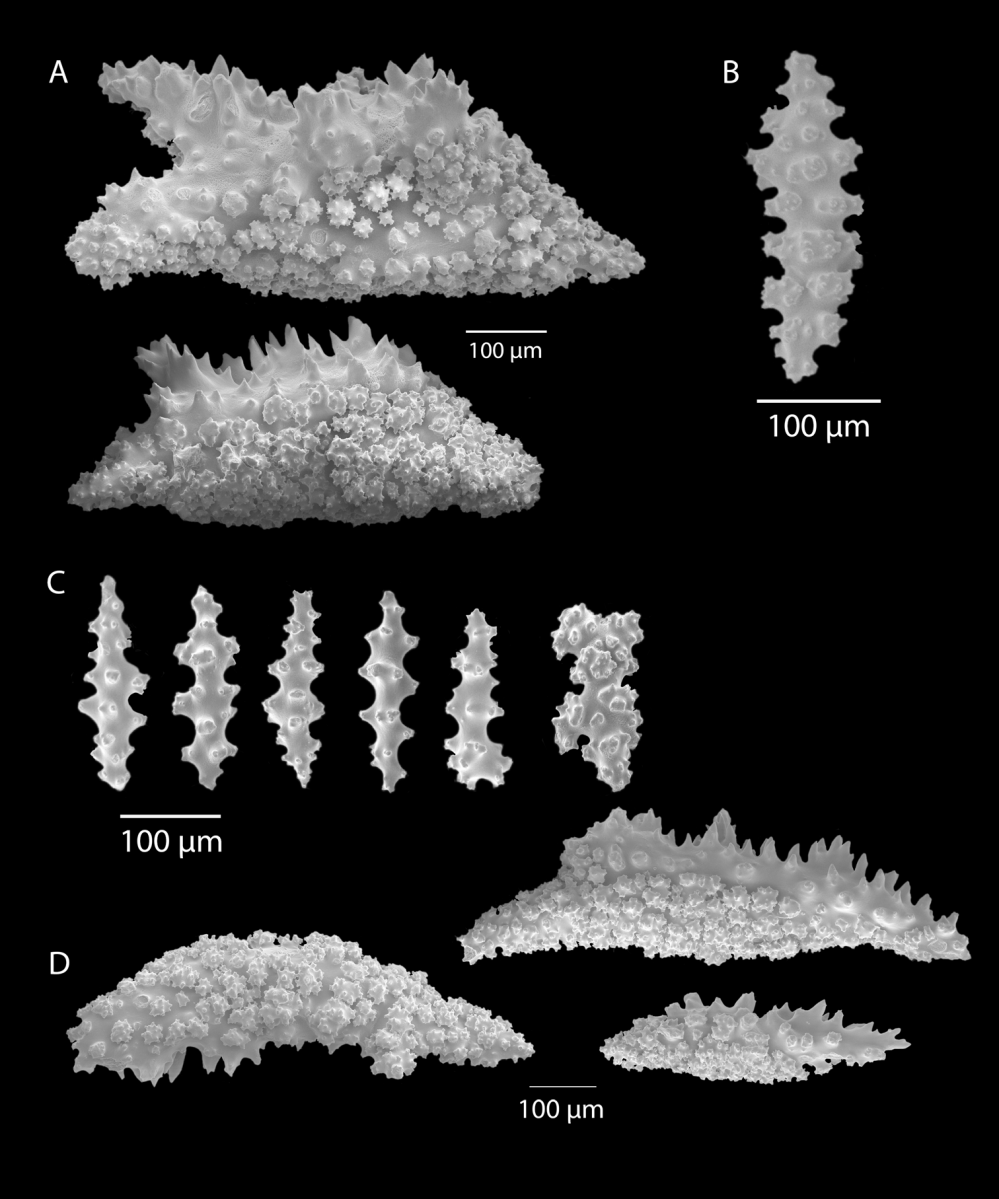
*Muriceafruticosa*, SBMNH 265940, SEM image. **A, D** Calycular and coenenchymal unilateral spinous spindles **B, C** Axial sheath **A** Large nudibranch-type sclerites **B** Typical spindle **C** Smaller spindles **D** tardigrade-type spindles.

**Figure 46.**
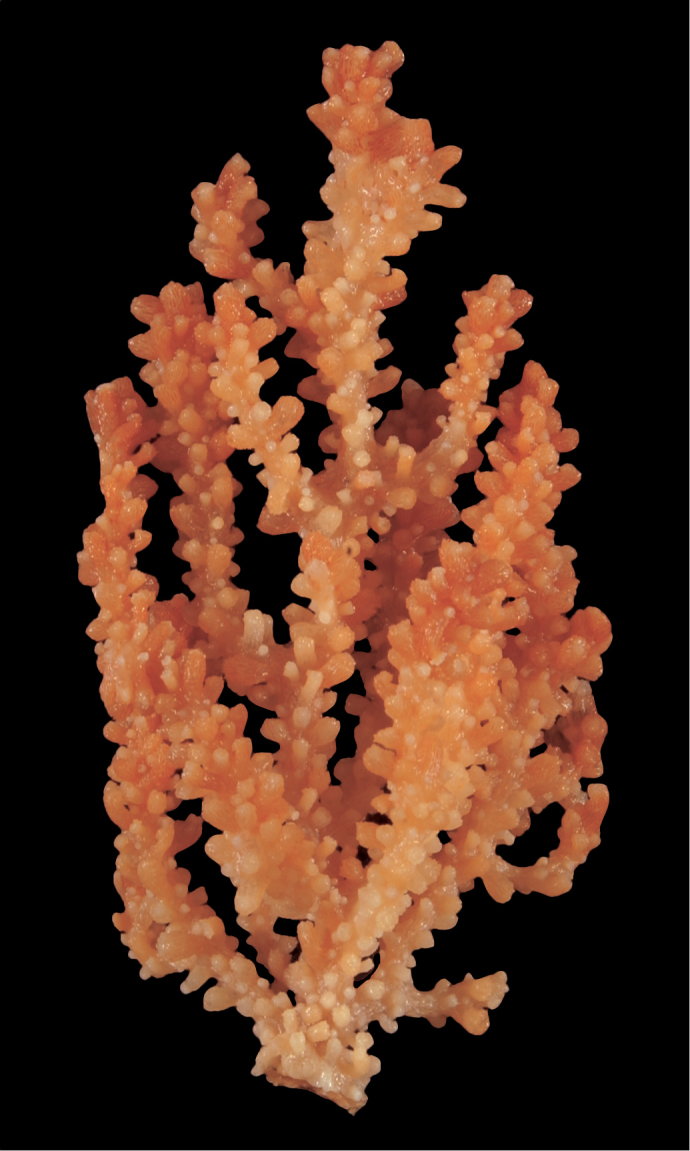
*Muriceafruticosa*, SBMNH 265945. Colony measures ~7.5 cm × 4.0 cm. Visible in image is the pale cream/yellow base/lower trunk; trunk intensifies and darkens to brownish orange towards branch tips. Notice swollen appearance of branch tips.

**Figure 47.**
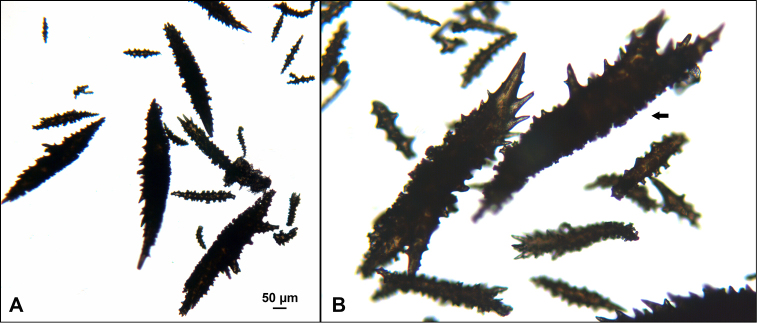
*Muriceafruticosa*, SBMNH 265945, light microscopy arrays. **A** 4× magnification, showing large spindles with pronounced conical spinules; central, vertically aligned one, measures 650 µm **B** 10× magnification of similar large spindles. Sclerite denoted by arrow is 0.875 mm in length, with a maximum length for this spindle form ~1.0 mm.

**Figure 48.**
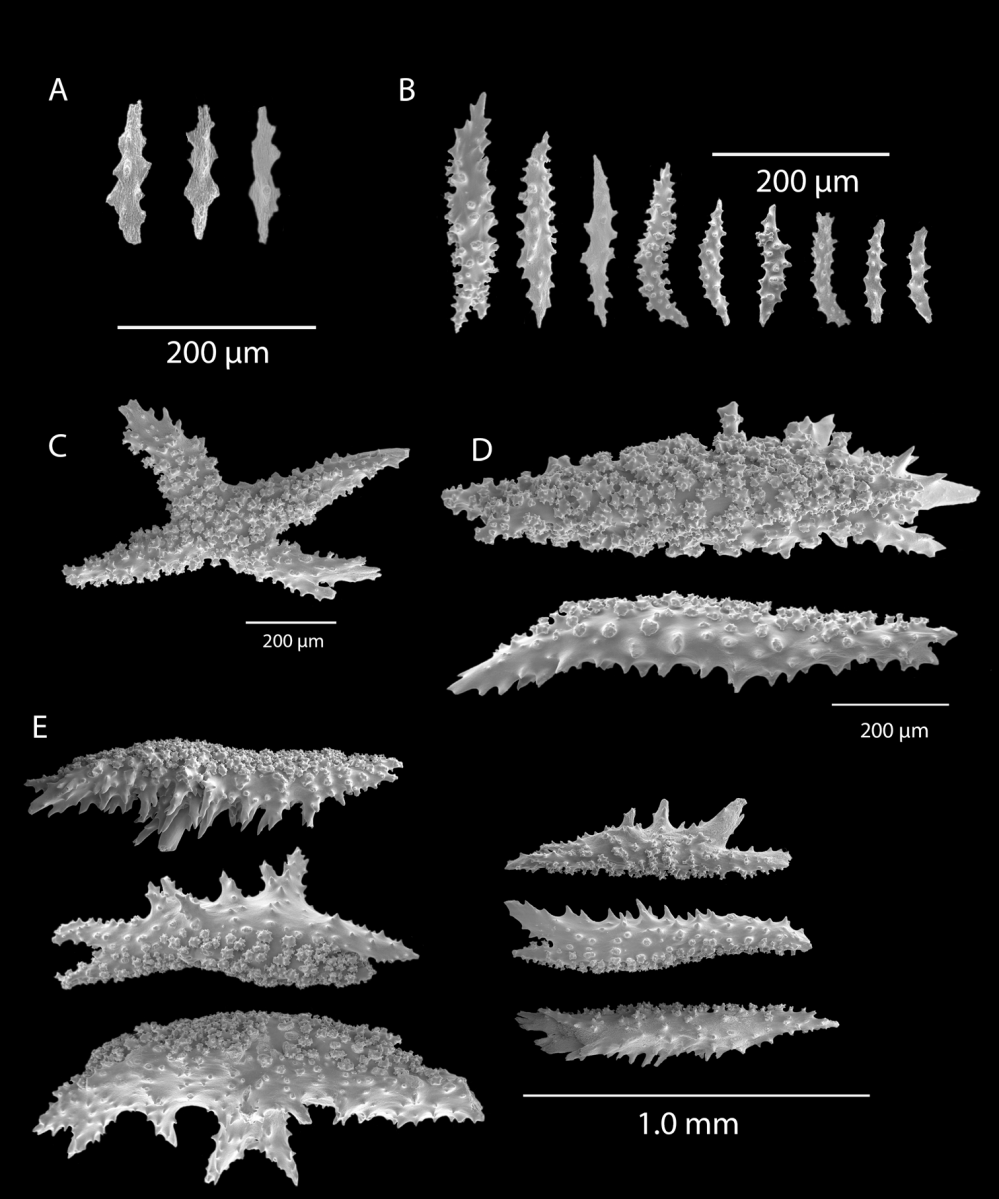
*Muriceafruticosa*, SBMNH 265945, SEM image. **A** Possible anthocodial sclerites **B** Spindles from the axial sheath **C** An unusual quadriradiate spindle from coenenchyme **D** Calycular/coenenchymal unilateral spinous spindles **E** Prominent calycular/coenenchymal tardigrade-type spindles.

Figures 43A, B , 44A, B , 45A–D , 46 , 47A, B , 48A–E

Muricea fruticosa Verrill, 1868a; 1869a: 428–430; pl 7, fig. 2. Kükenthal 1919: 752; 1924: 142–143. Hardee and Wicksten 1996: 129–130. Breedy and Guzmán 2016b: 9–14.
Muricea fruticosa var. typica Verrill, 1868a; 1869a: 428–430. Kükenthal 1924: 142.
Muricea fruticosa var. miser Verrill, 1869a: 430. Kükenthal 1919: 752; 1924: 143.
Thesea crosslandi Hickson, 1928: 354–356 (syn. nov.).
Pseudothesea crosslandi (Hickson, 1928). Stiasny 1943: 64–66 (syn. nov.)

###### Type locality.

(**Lectotype**) Panama, Pearl Islands, 11–14 m.

###### Type specimens.

**Lectotype** YPM 1574C [dry]; additionally, YPM 1660 [wet], YPM 1792 (fragment from Lectotype) [dry], and YPM 3067C [dry], are all listed as **Syntypes**. There are several specimens listed as **Syntypes** at NMNH: for instance, USNM 52292 [dry], with SEM images from stub #239. Syntype material at NMNH was examined.

###### Material examined.

~14 lots (see Appendix 1: List of material examined).

###### Description.

*Colony* (Figures 43A, 46) fairly large; dense, abundant branching; not reticulate. Branching irregularly dichotomous (also seen as cespitose/fruticose, tightly bush-shaped variant, with rather small, somewhat clavate branchlets outside California Bight; variant description not discussed here). Prominent, spreading, spinose calyces create rough texture to branches of colony. Colony (of Figure 46) very bushy; not in one plane. Colony stands up to three feet (90 cm) tall (Figure 43A), but usually shorter (30 cm). Main stem stout, short, arising from large, irregular base, usually dividing at once into several large, thick, unequal main branches, rapidly dividing and subdividing in irregular manner; branching extensive, such that main branches soon lost among crowded, crooked secondary branches. Branches and branchlets usually not more than 7.0–12 mm apart; branching can be in one plane, but not always. Small branches near ends often divide in irregular dichotomous manner, sometimes coalesce; very numerous, nearly equal in size, usually distinctly curved and crooked, spreading out at origin with a broad curve. Terminal branchlets short, 7.0–40 mm long, 2.0–3.0 mm thick, often curved, not tapering, either ending evenly or clavately, with obtusely rounded ends. In overall appearance, can look much like the colony shape of *M.californica* (perhaps reflective of similar environmental conditions). Color of living colony generally brown (darker) with white polyps; mostly deep reddish orange, rusty-brown; branchlet tips and calyces deep reddish brown, color generally fading to yellowish brown in proximal portion of branchlets, fading into light yellow, tinged with brown in main branches and trunk. Dry specimens orange-rusty brown, while polyps are pure white, situated on upper side of calyces; near distal end, aperture filled with yellow polyp sclerites, arising from bases of tentacles. Horn-like axis yellowish wood-brown at base and in larger branches (Hardee and Wicksten 1996 stated axis dark brown at base); darker reddish brown, translucent in smaller branches; light amber-yellow, translucent, slender up into branchlet tips. Calyces close together, but not overlapping, spreading outward and upward, 45 degrees from branch when closed, nearly 90 degrees with polyp extended; prominent, with conspicuous shelves opening distally, conical to columnar, larger and closer (1.0 mm) toward tips (Figure 43B), approximately as high (1.0–2.0 mm) as they are broad (1.0–1.5 mm); on larger branches low, rounded, without prolonged lower lip, better developed than at base where they are flatter, small and spread apart (2.0 mm). Those better developed have an obvious lower lip, sharp and long with very large, long, stout sclerites as spindles (some of which approximately as long as the calyces) which lie parallel to each other, projecting past upper margin of lower lip, giving colony a prickly feel when touched; upper lip small or barely noticeable. As calyces do not overlap, outer, thin coenenchyme easily seen lying between calyces, characterized by extremely large, stout, sclerites, visible to naked eye, and curving around them, often larger than calyces; these sclerites may be missing near base of colony or in poorly preserved specimens. Sclerites (Figure 44A, B) vary in color from brownish yellow and yellowish white to deep reddish brown. Largest sclerites (of outer coenenchyme), reddish orange-brown in color, up to 3.0 mm long; several shapes visible (Figures 44A, B, 45, 47A, B, 48A–E). One shape, very distinct, mostly stout, blunt and truncate, almost rectangular; longer, large, massive ones rather thick in middle, tapering somewhat abruptly at ends, densely, evenly covered by small tubercles (Verrill 1868 stated longer ones covered by small, sharp spinules on one side; other parts covered with crowded rough warts; these I refer to as tardigrade-like sclerites; Figures 44A, B, 45D, 47B, 48E). Second largest form irregularly fusiform, covered with tubercles; third form (Figure 48D) hook-shaped either on one end, both ends curving inward, or one end forked, the other tapering to a point; the latter often covered on ends with small, sparse, occasionally spiny tubercles, becoming more densely covered with tubercles toward the center, usually found around base of calyces. Medium-sized sclerites (~1.0 mm) more regular, fat (stout) in middle usually tapering to acute points; one side or one end covered with quite large, very sharp, simple projections, other side with densely crowded, rough microtuberculate warts. Sclerites of inner coenenchyme distinctly smaller, color ranging from yellow to white, fusiform, slender, often tapering to sharp point, covered with distinctly raised tubercles or warts (Figure 48B). Calyx sclerites long, up to 1.6 mm, very irregular and oddly shaped, mostly fusiform, one end often forking slightly or very noticeably, evenly covered with distinct tubercles, with some unilaterally spinose projections.

Generally, sclerites shown in Hardee and Wicksten (1996) appear to have extremely dense coverings of warts and bumps, the latter smallish in size. As well, they seem to cover a greater portion of the surface area of the sclerite as compared to those seen in drawings for sclerites of *M.californica*. Examinations of sclerites, for specimens identified as this species, reveal that sclerites can have flame-like teeth, these running almost the full length of the sclerite, on a side (what are termed nudibranch sclerites (Figure 45A), as those of Matamoros-Rosales (1984), rather than at an end such as seen in a torch. Very largest sclerites (tardigrade-like) rounded, densely warted, with projections coming off one of the longer sides, creating the appearance of legs and claws (like those of a tardigrade; Figures 44, 45D, some in Fig. 48E). Generally, sclerites have very dense tubercle coverings. The largest sclerites in this species are decidedly larger than those of *M.californica*; this will be the case despite what might have been concluded from having only looked at polyp color. This agrees with data provided in both Harden (1969) and Grigg (1970).

The colony shown in Figure 46 displays a few odd features, warranting further mention. Branching primarily pinnate to dichotomous. Colony measures ~7.5 cm tall, 4.0 cm wide. Slightly central main stem runs entire height of colony, with some slight curving laterally in random sections of stem; multiple branches all begin directly above or from base, many coming off of main stem. Branches often angle out a very short distance then curve upwards. Primary branches average 3.0–4.0 mm long, diameter ~1.0–2.0 mm (excluding calyces); diameter appears consistent from axillary branch points to branch tips. Calyces very columnar (1.0–2.0 mm wide, ~3.0 mm tall), heavily covered with longitudinally-oriented sclerites; polyps, many partially to fully extended from calyces, cream or light yellow; appear smooth. Calyces on all sides of branches, in some areas of colony very dense, in other areas calyces with some little distance between themselves, where sclerites can be seen on coenenchyme surface, lying sometimes longitudinally with the branches, sometimes not; latter more transverse (or oriented slightly in a triangular pattern) at base of calyces; calyces cover entire colony, right down onto coenenchymal surface of colony’s base. Calyces generally appear distinct reddish brown due to large, conspicuous sclerites. Color of colony generally darker distally, reddish brown, grading from free branch tips of colony to much lighter yellow or cream proximally, in stem and base (due to light-colored sclerites, usually small in size); polyps appear very light yellow, cream to white. Majority of calyx-bearing polyps appear such that they give colony an overall swollen appearance (as though branches of colony are covered with small, round grapes). This is the most unique feature of this colony; this may reflect an active reproductive state at the time of collection; specimen’s reproductive condition is still a question. Some calyces, scattered on all branches, tend to curve upwards slightly. The sclerites shown in both light microscopy (Figure 47A, B) and SEM (Figure 48A–E) in some ways match those for the colony of Figures 43–45, and yet in other ways do not clearly point to it being the same species. Based on this single specimen in the SBMNH collection (SBMNH 265945), from Long Beach, California, it requires further study. The biology of this colony, and its swollen appearance, has not been further explored or explained to date, other than to indicate that specimen was collected from very far into the back channels of Long Beach Harbor, quite a distance from open water (D Cadien, LACSD, pers. comm.).

###### Etymology.

Latin, *fruticosu*- meaning shrubby (bush-like); Verrill gave no specifics as to the derivation of the species name.

###### Common name.

Brown gorgonian; Fruitful purple one; Bushy rust gorgonian; Robust gorgonian (as indicated in various field/diving guides).

###### Distribution.

Potentially from Panama, up along California coast, perhaps as far north as Los Angeles County, California, with maximum, though infrequent, northern limit Point Conception, California. In a list of California sites, showing depth ranges, the following are indicated: **Mainland**: Point Loma: 5–14 m; La Jolla: 3–12 m; (USNM 50192 was collected at 11 m at La Jolla, in the Torrey Pines kelp bed, 5 miles north of Scripps Institution); Newport Beach: 0–12 m; San Pedro: 0–15 m; Santa Barbara: 5–8 m; Naples Reef: 5–11 m; **Islands**: Coronados Islands: 2–12 m; San Clemente Island: 21 m; Santa Catalina Island: 5–18 m.

###### Biology.

Seen more commonly in southern areas of California kelp beds (Ricketts, 4^th^ Ed. 1968); also, offshore pilings. Seen as well in lowest intertidal zone, outer Los Angeles Harbor; one of the most common species in southern California, in 15–30 m depths, Point Conception to Baja California (Grigg 1977; Gotshall 1994, 2005). Both this and *M.californica* seem to prefer subtidally occurring solid substrata at depths between 1.0–30 m (Grigg 1977). Lissner and Dorsey (1986) reported that while this species is common around the California Channel Islands and rocky areas of the mainland, it is conspicuously absent on the Tanner and Cortes Banks and the Santa Rosa – Cortes Ridge. Grigg (anecdotal communication) reported that populations of *Muricea* may be limited by cold water and/or poor dispersal abilities of the larvae. Grigg (1974) stated that this species is ca. one-tenth as abundant as *M.californica* off La Jolla. Based on work done by Grigg, it was estimated that a colony 30 cm high is ~20 years old. As very few colonies are seen larger than that, few colonies likely exceed this age. Mortality attributed mostly to abrasion, occurring when particulate matter is suspended in the water during periods of high waves and by smothering coming from accumulations of sand. One untitled and unpublished identification guide stated that colonies are able to survive in some of the most polluted near shore waters, as well as uncontaminated offshore waters. It appears this species is immune to encrustations known to cause mortality in *M.californica*. (SBMNH 422390, collected by MacGinitie in Newport Bay, if indeed this species–original identification indicated it was, based on white polyps, but sclerites do not support the identification–does have several galls produced by an acorn-type barnacle, on bare axis as well as on an area covered by coenenchyme.) Apparently fed upon by only one species of fish, the Garibaldi *Hypsypopsrubicunda*. Grigg (1974) calculated that between 10% and 15% of annual growth of this species and *M.californica* is cropped by Garibaldi predation.

###### Remarks.

Gift of FH Bradley; collected below low, low-water mark. Panama and (?) Pearl Islands, 6–8 fm (11–15 m) (Verrill 1869a). Identified as Muriceafruticosavar.typica (?).

There are two specimens at NMNH identified as this species, with SEM images: USNM 57171, SEM 237-238 (label reads ‘Albatross’ 28-29) and also, “*Muriceafruticosa* Verrill,” collected by Limbaugh, from St. (Cape) Lucas Canyon, SEM image 999. NMNH has an additional lot (USNM 52486), collected at Point Vicente, California, along with material in the “Limbaugh” Collection, which could well be this species, having been collected from the following locations (multiple lots): from California, at Huntington Beach Gas & Electric Steam Plant discharge pipe; from Baja, at Turtle Bay.

For all colonies of *Muricea* found in the California Bight, there is the possibility of other species in the genus not previously reported as appearing in the Bight, which may have very similar shape, etc. to the commonly recognized species described here. Perhaps there are new, undescribed species. It may well be that previously described species other than the standards make appearances in the Bight, and do so more often than previously thought. This possibility is supported by the fact that climatic factors can greatly expand ranges, even if only temporarily. A number of Mexican species may occasionally (or more often) make an appearance in the California Bight during certain substantial climatic/atmospheric events, such as an El Niño.

This conclusion has some support; Hardee and Wicksten (1996) in their closing paragraphs state “(a)lthough our material could be identified as (simply) *M.fruticosa* and *M.californica*, it is possible that other species of *Muricea* occur in southern California.” They recommended that a comparison of a series of specimens be made. This would help to clarify which previously described species are valid and which are “merely growth forms.” I agree with that recommendation. Fresh *Muricea* colonies, collected in a very intentional manner, from south to north, both within the Bight and to the south outside the Bight, must be done; that collection process is underway, with the help of the Santa Barbara Museum of Natural History’s Sea Center staff, staff of the Aquarium of the Pacific in Long Beach, California and staff of both the Los Angeles (LACSD) and Orange County (OCSD) Sanitation Districts. With the dramatic weather events we have recently experienced here in California, it will be interesting to see whether other species of the genus are making an appearance, for any length of time.

##### Genus Placogorgia

Taxon classificationAnimaliaAlcyonaceaPlexauridae

Studer, 1887

Placogorgia Studer, 1887: 56 [without species]. Wright and Studer 1889: 113. Nutting 1912: 83. Kükenthal 1919: 841; 1924: 209–210. Deichmann 1936: 141–142. Bayer 1956a: F206; 1959b: 54–55. Grasshoff 1977 (pars): 26. Muzik 1979: 80–81.
? nec Placogorgia Nutting (part), 1910a: 76 [= Discogorgia Kükenthal].
Clematissa Studer, 1887: 106–107.
Pseudothesea Kükenthal, 1919: 843.
Discomuricea Gordon, 1926: 521.

###### Type species.

*Placogorgiaatlantica* Wright & Studer, 1889; SM Wright and Studer 1889 (= *Pseudothesea* Kükenthal, 1919).

###### Diagnosis.

Colonies usually branched laterally in one plane; main stem generally long; primary branches with tendency to curve upwards; primary branches tend to run parallel with main stem, tips button-shaped, prominent swellings. Polyp height moderately low, on all sides of branches; especially dense at branch tips. Calyces truncated, cone shaped, armed with spindles (thorn scales). With crown (collaret) and points arrangement (= operculum of Paramuricidae): each of eight points composed of two-three pointed, convergent spindles in triangular arrangement above collaret of spinous rods, latter forming spiny transverse ring; fairly large triangular space free from sclerites between each point, situated in tentacle base. Thorn scales of calyx typically large, coarse, thick; wider than tall, each with broad, abundantly branched basal root (broad, flat), and a (usually) short, stout, more or less laciniated but usually strong, blunt spine; these sclerites overlap like roof tiles. Coenenchymal sclerites diverse spindles, simple, branched, often flattened, occasionally with one or more projections. Outer coenenchyme with long, often bent, sclerites (spindles), blunt points on both ends; at calyx base these form enclosing annular ring.

##### Placogorgia

Taxon classificationAnimaliaAlcyonaceaPlexauridae

species A

**Figure 49.**
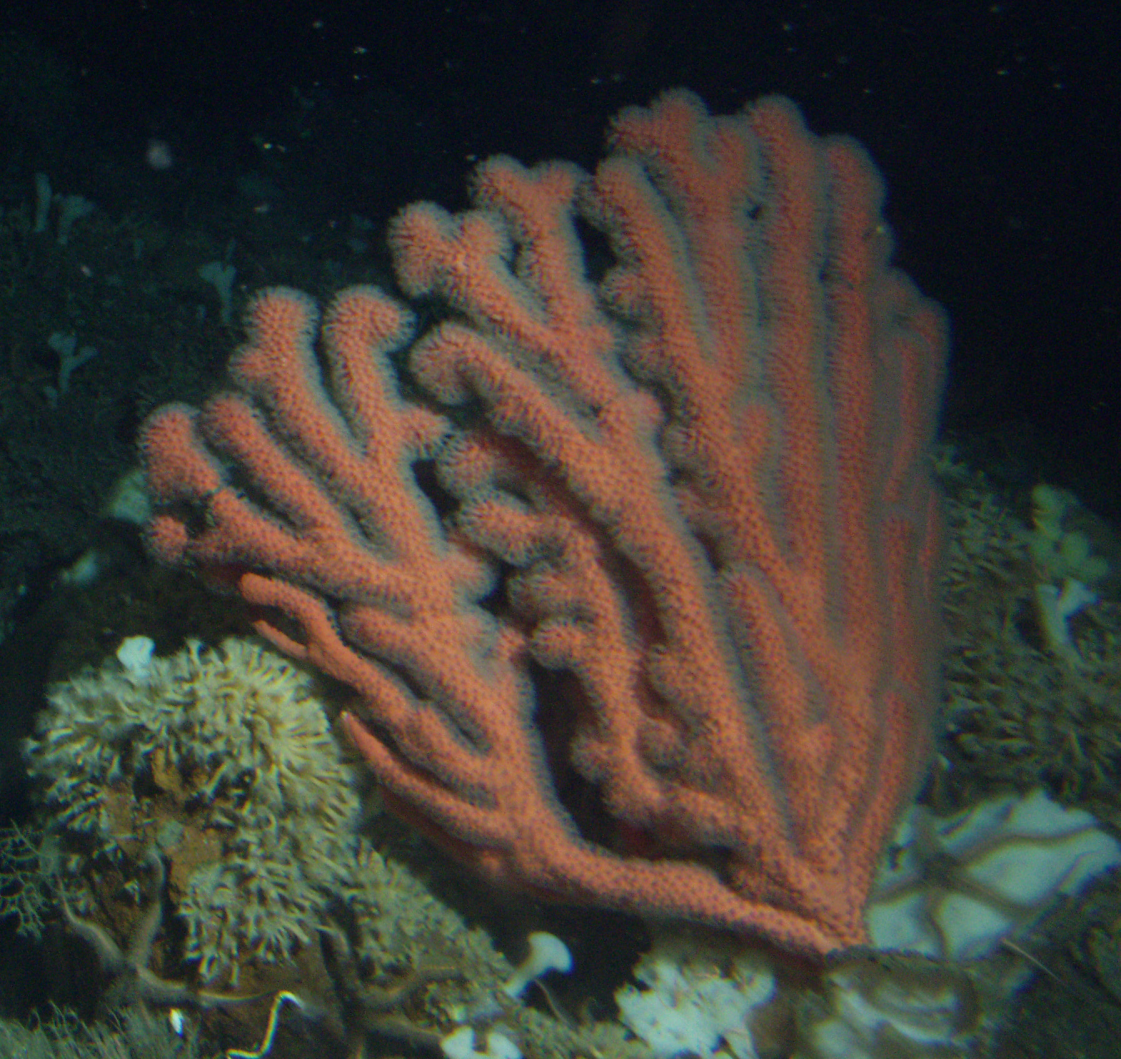
*Placogorgia* species A. In situ image, as seen in the Santa Barbara Channel. Image 6435B_Snook_018, taken by L. Snook. Image courtesy of Milton Love, UCSB.

**Figure 50.**
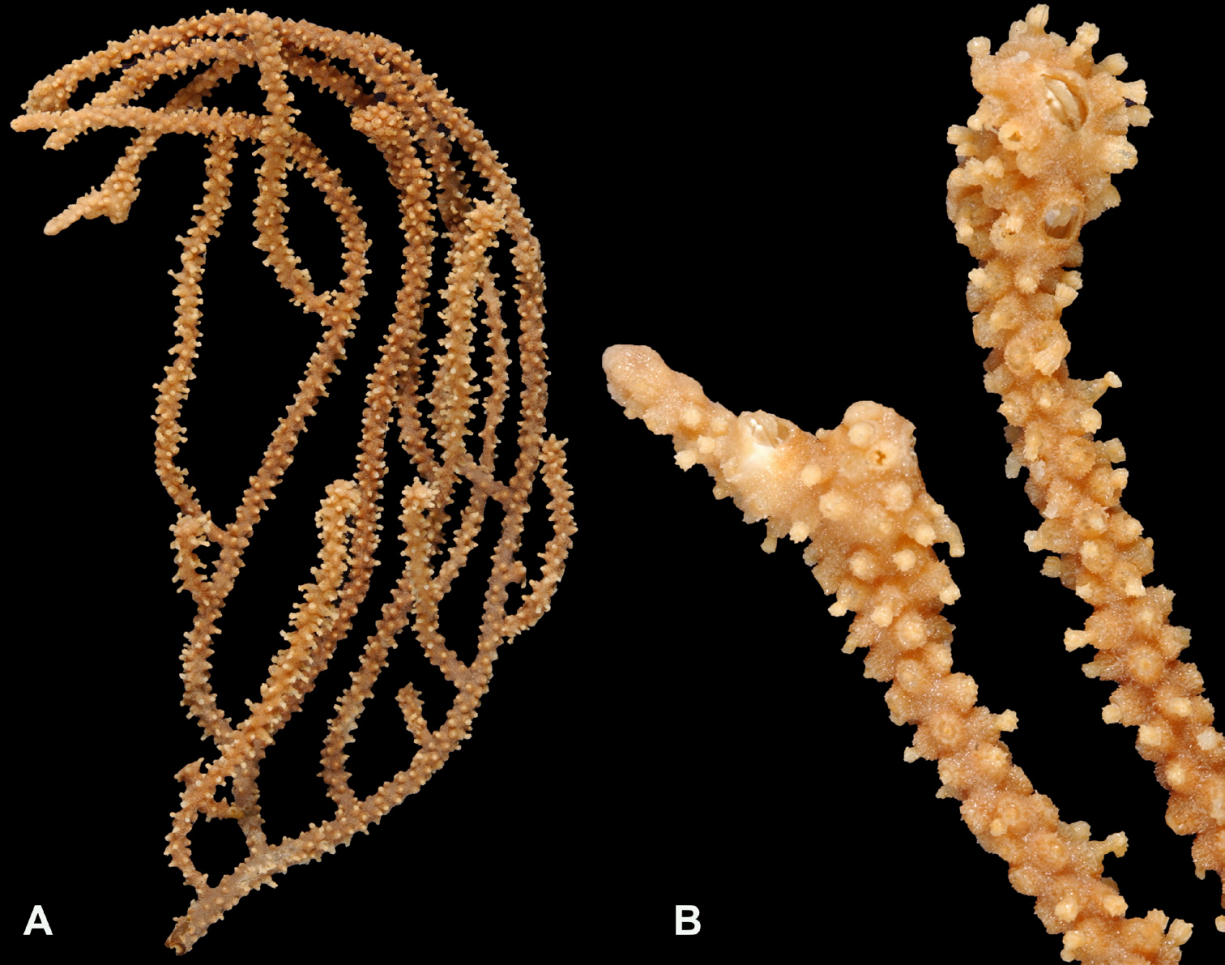
*Placogorgia* species A, SBMNH 422970. **A** Colony measures 28 cm tall (excludes missing base) × 9.0 cm broad **B** Magnification of branch tips; one tip malformed due to presence of barnacle gall.

**Figure 51.**
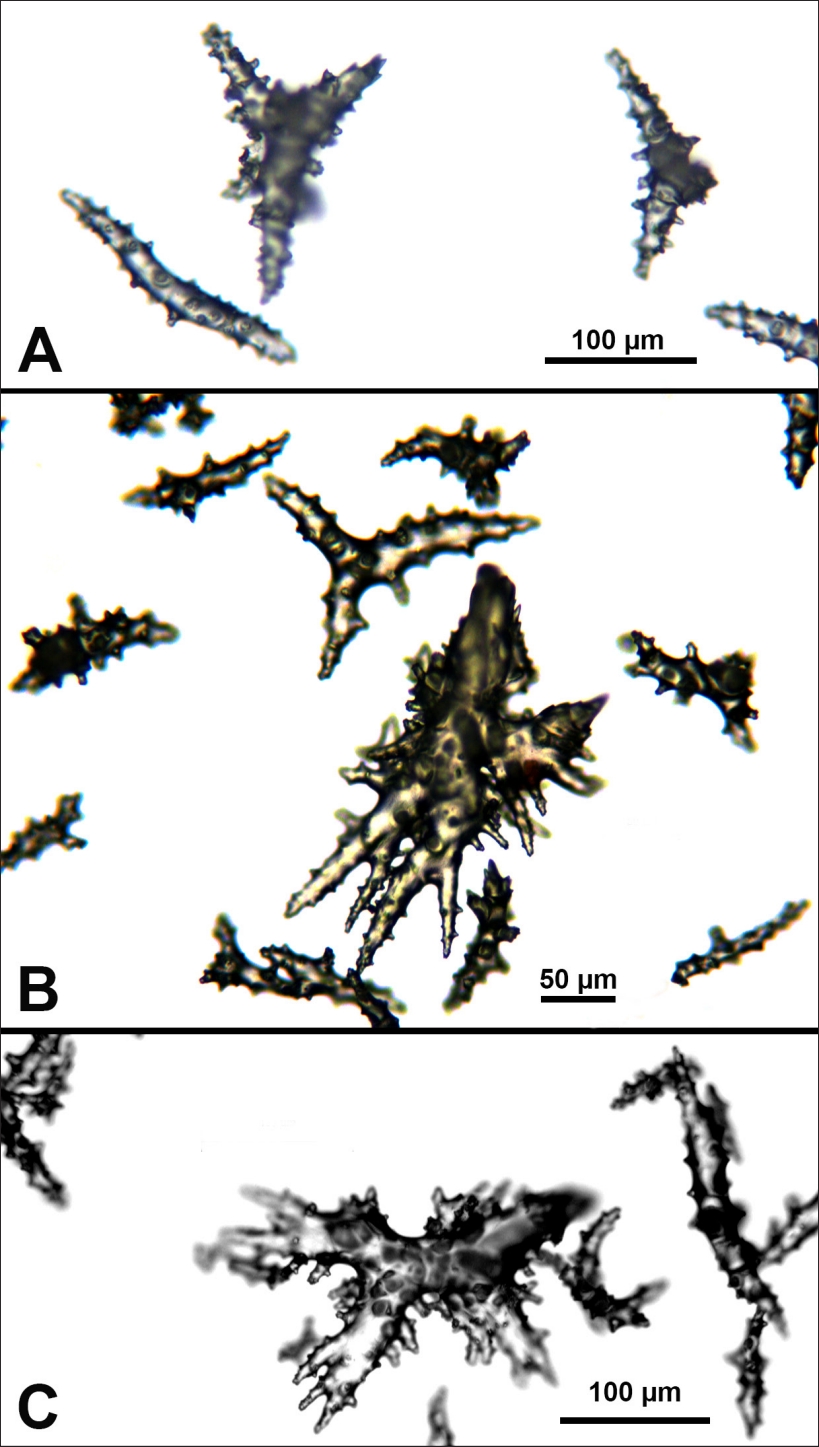
*Placogorgia* species A. SBMNH 422970, Light microscopy arrays, 10× magnification. **A** Some of the more unusually shaped sclerites **B–C** Several highly ornamented and distinctive sclerites. Crutch form ~80 µm tall, thin spindles measure in the range of 150–191 µm in length, larger, thicker spindles are ~250 µm long, with unusual forms, such as **B**, the tall, spikey sclerite measuring some 300–350 µm tall and that appearing as a very large quadriradiate (**C**) measuring 280 µm across.

**Figure 52.**
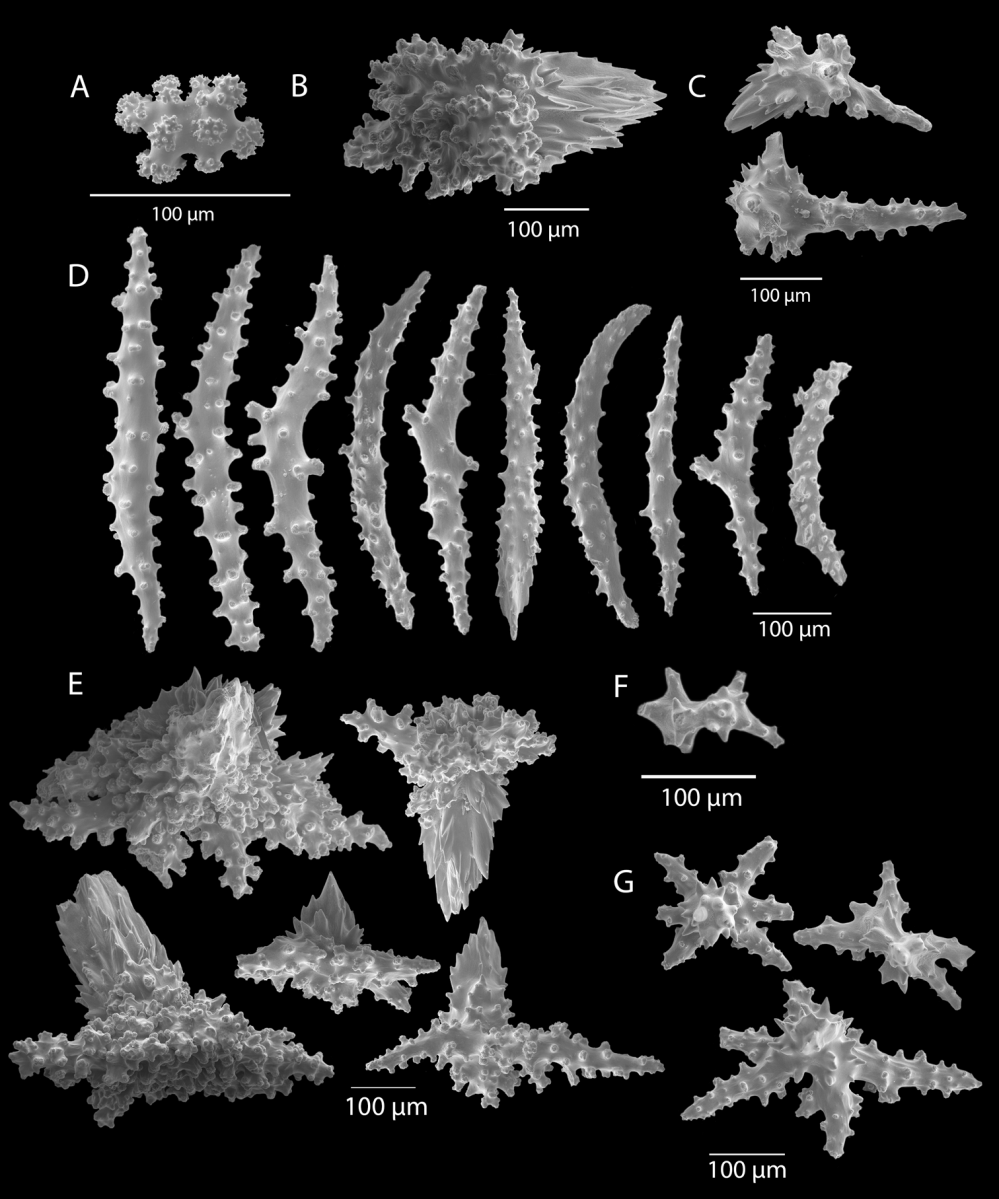
*Placogorgia* species A. SBMNH 422970, SEM image. **A** Small capstan form **B** Chunky torch-type spindle **C** Crutch-type spindles **D** Long spindles **E** Thorn scales typical of the genus **F** Small, developing thorn scale, dorsal view **G** More developed thorn scales, dorsal view.

Figures 49 , 50A, B , 51A–C , 52A–G

###### Type locality and type specimens.

Until there is species confirmation, no information can be provided.

###### Material examined.

~5 lots (see Appendix 1: List of material examined). While labeled *Placogorgia* material was examined at NMNH, nothing resembled in any way the specimens in the SBMNH collection.

###### Description.

*Colony* (Figures 49, 50A) generally branched in one plane; rarely, few reticulate; colony height (base to tip of upper-most branch) approximately 20–21 cm.; long, generally dichotomous branches and branchlets, moderately thick, cylindrical; branch diameter thickness averages 5.0 mm (including calyces); meandering sinuously, branches bent upward, parallel with main stem (not always obvious); tips of branches and branchlets swollen, to rounded 8.0-mm diameter (Figure 50B). Main stem bifurcates (sometimes), some distance (±10 cm) from base; branches then again bifurcate at ~1.0->5.0 cm from first division. Further branching asymmetrical. Not all branches subdivide; of those that do, distance from previous subdivision varies. Polyps distributed over entire surface, sparsely placed at base, becoming progressively most crowded at branch tips. Color of freshly collected specimens, via video and still image (Figure 49), pale pinkish tan with conspicuous, fluffy, cotton candy-pink polyps; in preserved (dry) specimens, color dull tan-brown (Figure 50A, B); axis color slightly darker brown. Calyces moderately low, ~1.0 mm tall, 2.0 mm across, 2.5 mm apart; blunt/rounded, conical, armed with sclerites of various shape. Coenenchyme (relatively thin) contains long, blunt-ending spindles, often bent; largest bent spindles 0.3–0.6 mm L, 0.05–0.1 mm H (average 0.5 mm L × 0.08 mm H), often with strong external spines (Figure 52D). Distinctive sclerites often like thorn-scales or thorn-stars; small, spinulate or laciniate ones predominantly calycular (Figures 52E–G). Anthocodial sclerites difficult to extract; sclerites of collaret also blunt-ended, bent spindles, at base of polyp, tending to circular arrangement. A few as large, heavy, tapered spindles, sometimes with several heavy, rough spines projecting from one side; some few (the calycular sclerites) as branched torches (Figure 52B); also, crutch-types (Figures 51A, 52C); some few as crosses and irregular ones, most as unilateral spiny shapes (Figure 51B, C). Color of sclerites cream to very light tan, at least in specimens that are long dry.

###### Distribution.

For genus (based on material found/examined at NMNH and other institutions, such as CAS) from Point Conception (California Bight) to Gulf of California (eastern North Pacific Ocean); western Pacific from Hawaii south to Philippines and Indonesia. NMNH also has specimens in this genus collected from off the coast of Chile in the southeastern Pacific Ocean; these look very different from the one described here.

###### Biology.

May be considered a subtidal to deep-sea genus; this based on collection data for known species.

###### Remarks.

Bayer’s review of the genus (1959b), and description of a new species from Florida, was invaluable for understanding the SBMNH specimens; a thorough discussion of both the calycular thorn scales and the cortical sclerites was provided. For specimens described here, the calycular thorn scales were difficult to extract; most of the material studied was quite dry, but an examination of wet specimens (SBMNH 422968 and 422970) revealed that the form labeled “E” in Bayer (1959) is the closest fit to what is seen in these specimens. Cortical sclerites in these specimens best fit those labeled “K,” “L,” and “M” (latter, in part; Bayer 1959). As well, sclerites seen in these specimens also corresponded as follows to Diechmann’s (1936) illustrations for several species in the genus *Placogorgia* from the Atlantic Ocean: pl 15, figs 19–20 best match those identified as cortical thorn scales, pl 15, figs 23–24 best match those likely calycular (branched torch) thorn scales, while pl 15, figs 26 and 32 match the blunt-ended spindles seen here. Nearly all sclerite shapes in this unidentified species are far broader in their root than they are tall; based on interpretation of the key in Bayer’s review I have made a tentative placement of this species in the genus *Placogorgia*.

There are multiple species of *Placogorgia* from the Atlantic, as well as a number of species from the South Pacific and Indian Oceans (Kükenthal 1924). Only a few records were found from the southeastern Pacific: unidentified species found west of southern Chile (USNM 80162 [wet] and 98863 [wet]), and a number of unidentified specimens from the western North Pacific (Hawaii), such as USNM 75077; none were from the eastern North Pacific. There was one reference by Harden (1979) indicating that the species, using one of the same specimens currently discussed here (SBMNH 422969), is *Placogorgiaramosa* (Wright and Studer 1889), stating that it is synonymous with *Paramuricearamosa* (Wright & Studer, 1889). Cordeiro et al. (2018i) does not list a species *P.ramosa* in the list of recognized *Placogorgia* species in the WoRMS Database; Harden’s designation does not inform the identification of the SBMNH specimen in any way. As well, I could not find any other indication that *Paramuricearamosa* is synonymous with *Placogorgiaramosa*.

Muzik (1979) stated that coenenchyme in species of *Placogorgia* is filled with diverse spindles that are simple, branched and often flattened, occasionally with one or more projections; this is descriptive of what was seen here. Muzik (1979) also stated that the calycular thorn scales are wider than tall, the projection usually short, with the base broad and flat. The Hawaiian species described by Muzik (1979) with their distinctive features were of interest: the presence of 1) broad calycal thorn scales and 2) very branched, flattened sclerites of the coenenchyme. It seemed best to place the examined specimens in the SBMNH collection within the genus *Placogorgia* based on the appearance of the sclerites, but other genera are under consideration. However, any link between an eastern Pacific locality and described members of this genus, or any other possible genera, cannot be made (Ekman 1935: 66, 1953: 40; Bayer and Deichmann 1960).

Should specimens in the SBMNH collection represent a new species, this would be the first description of a species from this genus in the northeastern Pacific Ocean; if not a new species, then this is the first record of a known species of *Placogorgia* (seen elsewhere) from the northeastern Pacific Ocean; further study of specimens is currently underway.

## Conclusions

The SBMNH research collection, including substantial material from the Allan Hancock Foundation’s ‘Velero’ Expeditions, provides a good representation of the families Gorgoniidae and Plexauridae (and many species included therein) from the area known as the California Bight. The collection effectively displays the variation (and differences) of species found in the California Bight as compared with other locations which may also harbor the same species. While many species from the collection match well with species collected in other locations, there are some intriguing differences seen in many specimens taken from within the Bight that reflect, perhaps, the dynamic environmental system that is the California Bight.

As the family Plexauridae is so well represented in the SBMNH collection, two of the remaining genera in the family, *Swiftia* and *Thesea*, will be discussed more thoroughly in Part III of this work, completing the full and comprehensive review of all species recorded to date as appearing in the California Bight, based on the SBMNH research collection, and specifically on the many specimens that came to SBMNH through the Allan Hancock Foundation’s historic ‘Velero’ Expeditions collection events.

## Supplementary Material

XML Treatment for Gorgoniidae

XML Treatment for Adelogorgia

XML Treatment for Adelogorgia phyllosclera

XML Treatment for Eugorgia

XML Treatment for Eugorgia daniana

XML Treatment for Eugorgia rubens

XML Treatment for Eugorgia ljubenkovia

XML Treatment for Leptogorgia

XML Treatment for Leptogorgia chilensis

XML Treatment for Leptogorgia diffusa

XML Treatment for Leptogorgia filicrispa

XML Treatment for Leptogorgia flexilis

XML Treatment for Leptogorgia

XML Treatment for Plexauridae

XML Treatment for Chromoplexaura

XML Treatment for Chromoplexaura marki

XML Treatment for Muricea

XML Treatment for Muricea californica

XML Treatment for Muricea plantaginea

XML Treatment for Muricea fruticosa

XML Treatment for Placogorgia

XML Treatment for Placogorgia

## Appendix 1

**List of material examined – Part II**

(Material examined = whole colony study plus multiple sclerite preparations; all with light microscopy, plus selected colonies under SEM, shown in figures associated with text)

***Adelogorgiaphyllosclera* Bayer, 1958**

**Material examined.** ~25 lots. **USA, California** – fragments, plus full colony; San Diego County, San Diego, Point Loma, 32°41'28"N, 117°16'53"W, station PL-13, no depth recorded; coll. SCCWRP, Sea Quest, 10 September 1975; LACoMNH Marine Biodiversity Center and SBMNH 422894 [wet]. – 1 fragment; Los Angeles County, off the southern California Channel Islands, seaward of Newport Beach, 33°34'02"N, 118°02'20"W, 96 m; coll. M Love via submersible ‘Delta’, 6 October 2005; (his number: A6651), SBMNH 422893 [wet]. –1 colony; Los Angeles County, Catalina Island, Bird Rock, 33°27'00"N, 118°29'14"W, 50 m; coll. R Given, July 1977; SBMNH 51252 [wet]. –1 colony; Los Angeles County, Santa Catalina Island, .33 miles, 180° T from Ship Rock Light, 33°27'19"N, 118°29'34"W, 58 m; coll. R/V ‘Velero IV’, station 2132-52, 22 July 1952; SBMNH 422308 [wet]. –1 colony; Los Angeles County, Santa Catalina Island, 4 miles, 140° T from Ship Rock Light, at Isthmus Cove, by dredge, 33°27'36"N, 118°28'58"W, 76 m; coll. R/V ‘Velero IV’, station 12400-68, 17 October 1968; SBMNH 423087 [dry]. –1 colony; Los Angeles County, off Santa Monica, South Banks, 33°53'00"N, 118°31'00"W, by trawl, 82–100 m; coll. unknown, 10 October 1978; SBMNH 422892 [wet]. **MEXICO, Baja California Sur (Pacific Coast)** – multiple fragments; southwest of Punta San Eugenio, 8.5 miles S of Canal de Dewey, 27°42'15"N, 115°05'02"W, 89 m; coll. R/V ‘Velero III’, station 1259-41, 27 February 1941; SBMNH 422309; [wet]. –3 colonies; Isla Natividad, 7.5 miles SSW of island, 27°44'17"N, 115°14'40"W, 115–120 m; coll. R/V ‘Velero III’, station 1258-41, bottom of loose rocks, coral, 27 February 1941; SBMNH 422403 [dry]. **MEXICO, Baja California Norte (Pacific Coast)** – multiple colonies; 8 miles W of Isla Cedros, 28°05'45"N, 115°31'35"W, 117–118 m; coll. R/V ‘Velero III’, station 1253-41, 26 February 1941; SBMNH 422310 [wet]. –multiple colonies/fragments; 1.5 miles off north end of Isla Cedros, 28°23'20"N, 115°11'52"W, 100–109 m; coll. R/V ‘Velero III’, station 1264-41, 28 February 1941; SBMNH 422311 [wet]. **CENTRAL AMERICA** – 1 colony, (no base); Panamá, Veraguas, off Bahía Honda, rock, sand and mud, 07°45'35"N, 81°35'35"W, 55–91 m; coll. R/V ‘Velero III’, station 863-38, 01 March 1938; SBMNH 423058 [dry]. **SOUTH AMERICA** – multiple fragments; northern Pacific Ocean, Ecuador, Galápagos Islands, Off Bindloe (=Marchena Island), 00°18'20"N, 90°31'00"W, 27 m; coll. R/V ‘Velero III’, Station 310-35, 3 December 1934; SBMNH 422312 [wet]. –1 colony (bleached); southern Pacific Ocean, Galápagos Islands, Santa Cruz Island, off Gordon Rocks, 00°33'30"S, 90°09'45"W, 46–55 m; coll. R/V ‘Velero III’, station 317-35, 8 December 1934; SBMNH 422891 (‘Velero’ label written in error, 317-37) [wet]. Note: Specimens listed from Central/South America possibly one or more of several new species recently described by Breedy and Guzmán 2018.

**Other material examined. USA, California** – 1 fragment; San Diego County, taken off shore, BI 53-4, SIO 61-517, 32°38'33"N, 117°15'34"W, 30 m, with dredge; coll. unknown, 19 November 1953 (0900); SIO-CO 457 [wet]. –2 fragments; San Diego County, SI 50, from Canyon Rim, 80 m; coll. unknown, 2 October 1965; SIO-CO 1683 (two specimens in the one lot) [wet]. –San Diego County, Canyon Rim, SL 49-b. 80 m; coll. R Grigg, 2 October 1965 (1200); SIO-CO 1605 [wet]. –San Diego County, off La Jolla, Station 8, La Jolla Canyon, 114-40 m, rock dredge–rock, sand, clay; coll. unknown, 7 January 1954; SIO-CO 1588 [wet]. –multiple colonies; San Diego County, La Jolla, La Jolla Canyon, South Wall, 64 m; coll. C Limbaugh and party, 11 September 1957; [NMNH collection, no catalog number, dry]. –Los Angeles County, Santa Monica, 33°52'08"N, 118°34'05"W, 73 m; coll. unknown, 29 March 1974; SIO-CO 1579 [wet].

**Other material, not examined. USA, California** – San Diego County, San Diego, 32°45'06"N, 117°30'00"W, 9–24 m; coll. C Limbaugh, Station 1166, 3 July 1906; USNM 57456 [wet]. –2 colonies; USA, California, San Diego County, La Jolla, 400 miles west of Point La Jolla, 18–21 m; coll. C Limbaugh, 14 December 1953; **Paratype**–USNM 50187 [dry]. –6 colonies; USA, California, San Diego County, La Jolla, La Jolla Canyon, 46–52 m; coll. C Limbaugh, 8 October 1955; **Paratype**–USNM 50188 [dry]. –1 colony; USA, California, San Diego County, La Jolla, La Jolla Canyon, 40–43 m; coll. R Ghilardi and party, 19 January 1956; **Paratype**–USNM 50635 [dry]. –USA, California, San Diego County, La Jolla, La Jolla Point, 32°51'01"N, 117°21'02"W, 104–130 m; coll. R/V ‘Albatross’, Station 4328, 8 March 1904; USNM 51320 [dry].

***Eugorgiadaniana* Verrill, 1868**

**Material examined.** ~10 lots **MEXICO, Baja California Sur (Pacific Coast)** – 1 colony; 2 miles, 220° T from Punta San Eugenio, 27°49'30"N, 115°05'10"W, 38 m; coll. R/V ‘Velero IV’, station 11514-67, 15 June 1967; SBMNH 422895 [wet]. **MEXICO, Baja California Norte (Pacific Coast)** – 1 colony; Isla Cedros, coast side, 28°13'00"N, 115°10'00"W; coll. Pacific BioMarine, received from Carmelita Freeman, 26 April 1974; SBMNH 422897 [DH 423 = SBMNH-10]; [dry]. –1 colony; Isla Cedros, 10 miles S of Punta Norte on east side of island, 28°13'00"N, 115°10'00"W, 9 m; coll. R McPeak, 22 February 1983; SBMNH 422404 (B1545) [dry]. **MEXICO, Baja California Norte (Gulf of California)** – 1 colony; Bahía de Los Angeles, Isla Mitlan, 29°04'00"N, 113°31'00"W, 6 m; coll. R McPeak, 12 September 1980; SBMNH 422405 [dry]. **MEXICO (Gulf of California)** – 1 colony; Sonora, Bahía de Cholla (= Choya), 31°20'46"N, 113°38'14"W; coll. J Wilkins, 23 February 1959; SBMNH 422896 [DH 433 = SBMNH-20] [dry].

**Other material examined** – fragment; northern Pacific Ocean, Mexico, Baja California Sur, offshore island of Isla Socorro, outside Punta Tosca, ~18/19°00'00"N, 112°00'00"W, 21 m; coll. unknown, 4 February 1964; SIO CO 1686 [wet]. –1 colony; northern Pacific Ocean, Mexico, Baja California Sur, Puerto (Bahía) San Bartolomé (Tortugas or Turtle Bay); coll. unknown, 14 March 1961; USNM 52484 [dry]. –fragment; USA, California, San Diego County, Loma Sea Valley, due west of Point Loma, by otter trawl 1, ST-908, 326–351 m; coll. Fager party, 21 January 1965; SIO CO 1684 (Acc. No. BI 65-33) [wet]. –1 colony; California, San Diego County, San Diego, Escondida Bay; coll. unknown, 14 November 1868; USNM 57302 [dry].

***Eugorgiarubens* Verrill, 1868**

**Material examined.** ~ 50 lots. **USA, California** – 1 colony; California Channel Islands, on a specimen shell of *Haliotissorenseni* (SBMNH 349096); coll. Leon Bray, 1964 [dry]. –1 fragment; Orange County, 1 mile S of Newport Harbor, 33°36'30"N, 117°54'30"W, vertical south face of shale reef, 17 m; coll. R Given, by hand, SCUBA, 05 October 1957; SBMNH 422313 [wet]. –1 colony (stripped); Santa Catalina Island, 1.5 miles, 130° T from Long Point Light, urchins, crinoids, dead brachiopod, diversified, very rich, 33°23'48"N, 118°21'16"W to 33°22'57"N, 118°19'54"W, 84 m; coll. R/V ‘Velero IV’, Station 2226-53, 28 February 1953; SBMNH 422314 [wet]. –1–2 colonies; Los Angeles County, Santa Catalina Island, between Avalon and Long Point, 33°23'30"N, 118°21'50"W, 55–80 m; coll. R/V ‘Velero III’, station 1167-40, 08 August 1940; SBMNH 422407; SBMNH-14 [dry]. –colony fragments; Los Angeles County, .33 miles, 180° T from Santa Catalina Island, Ship Rock Light, dredge–brachiopods, rocky sand bottom, 33°27'19"N, 118°29'34"W, 58 m; coll. R/V ‘Velero IV’, station 2132-52, 22 July 1952; SBMNH 422315[wet]. –multiple fragments; Los Angeles County, 7.35 miles, 89° T to Manhattan Beach Pier, 33°53'18"N, 118°32'45"W, 58 m; coll. R/V ‘Velero IV’, station 24493-76, 10 March 1976; (with label– AHF 24493, BLM Ref. station 360; Leg. Mo. 08); SBMNH 422099 [wet]. –fragment; Los Angeles County, off Rocky Point, close to Point Vicente, S end of Santa Monica Bay, ~33°44'33"N, 118°25'28"W; coll. by C Limbaugh; 5 October 1941; SBMNH 422898 [wet]. –multiple fragments; Los Angeles County, 10.75 miles W. of Point Dume, on loose rock, sponge, 34°00'00"N, 119°01'20"W, 87 m; coll. R/V ‘Velero III’, station 1276-41, 23 March 1941; SBMNH 422324 [wet]. –1 colony; Ventura County, Rincon, 34°22'27"N, 119°28'27"W, 98 m; coll. P Brophy, commercial otter trawl, 01 May 1968; SBMNH 422901 [DH 421 = SBMNH-08; dry]. –1 colony; Ventura County, Anacapa Island, 34°01'00"N, 119°21'43"W, 11 m; coll. B Scronce and M Conboy, by hand, SCUBA, 29 January 1964; SBMNH 422900 [DH 432 = SBMNH-19; dry]. –1 colony; Ventura County, Anacapa Island, 34°01'00"N, 119°21'43"W, reef and cove, northeast end of island, 11 m; coll. B Scronce and party, by hand, SCUBA, 29 January 1964; SBMNH 45561 [ex MacGinitie collection; wet]. –1 colony; Ventura County, Anacapa Island, 34°01'00"N, 119°21'43"W, reef and cove, northeast end of island, 17–18 m; coll. B Scronce and party, 29 January 1964; SBMNH 45562 [ex MacGinitie collection; wet]. –1 fragment; Ventura County, Anacapa Island, windward side, 34°00'36"N, 119°24'02"W, 15–24 m; coll. M Hamann and S Bobzin, 21 February 1998; SBMNH 345323 [wet]. –1 colony; Santa Barbara County, Santa Cruz Island, 34°01'14"N, 119°41'05"W, seaward side; 15–18 m; coll. W Hary, February 1967; SBMNH 422411 [dry]. –1 colony fragment; Santa Barbara County, basin off Santa Cruz Island (Santa Barbara Basin/Channel), 15.5 mi., 139°T, from Santa Barbara Light, Santa Barbara, CA, 34°20'15"N, 119°37'45"W, 51 m; coll. T Phillips, R/V ‘Velero IV’, station 13617-69, 13 November 1969; SBMNH 422409 [DH 428 = SBMNH-15; dry]. –small colony and fragments; Monterey County, Point Sur, 36°16'07"N, 121°55'13"W, 65 m; coll. T Laidig, NMFS-FED, Santa Cruz, 13 October 2007; SBMNH 235537 [dry]. **MEXICO, Baja California Sur (Pacific Coast)** – several fragments; 28 miles, 199° T from Abreojos Light, S of Punta Abreojos, dredge–brachiopods, #12 coarse sand and rock, 26°17'15"N, 113°41'45"W (end), 100 m; coll. ‘Velero IV’, station 1709-49, 7 March 1949; SBMNH 422327 [wet]. –1 colony; 6.5 miles, 167° T from Punta Abreojos, dredge–coarse mud and sand, mud bottom, 26°35'27"N, 113°31'45"W (end), 51 m; coll. R/V ‘Velero IV’, station 1954-50, 29 April 1950; SBMNH 422316 [wet]. –2 colonies; SE of Isla Asunción, pinnacles near the “6 fathom spot, 27°06'28"N, 114°17'30"W, 23–27 m; coll. RH McPeak, station B, 11-14 Nov. 1981; SBMNH 422412 [dry]. –2 colonies; 8.5 miles south of Canal de Dewey, sand, broken shell, gravel, 27°42'15"N, 115°05'02"W (end), 89 m; coll. R/V ‘Velero III’, station 1259-41, 27 Feb. 1941; SBMNH 422323 [wet]. **MEXICO, Baja California Norte (Pacific Coast)** – 1 colony; Islas San Benitos, 28°18'10"N, 115°33'20"W, in *Phyllospadix*, 5 m; coll. Santa Catalina Island Lab, 15 August 1977; SBMNH 422413 [dry]. –1 colony; 8 mi. SW of Isla Cedros, green, fine sand, coral, 28°00'00"N, 115°29'00"W (end), 115–118 m; coll. R/V ‘Velero III’, station 1254-41, 26 Feb. 1941; SBMNH 422318 [wet]. –several colonies; 8 miles W of Isla Cedros, gravel, loose rock, 28°05'45"N, 115°31'35"W (end), 116–118 M; coll. R/V ‘Velero III’, station 1253-41, 26 Feb. 1941; SBMNH 422317 [wet]. –1 colony; 1.5 miles off N end of Isla Cedros, fine sand, broken shell, 28°22'18"N, 115°11'00"W (end), 82–100 m; coll. R/V ‘Velero III’, station 1263-41, by small dredge, 28 Feb. 1941; SBMNH 422329 [wet]. –1 fragment; 1.5 miles off N end of Isla Cedros, shale, pebbles, 28°23'20"N, 115°11'52"W (end), 100–109 m; coll. R/V ‘Velero III’, station 1264-41, by small dredge, 28 Feb. 1941; SBMNH 422328 [wet]. –multiple fragments; 6.5 miles SW of Punta San Carlos, dredge, rocky bottom, 29°33'30"N, 115°35'28"W (end), 36 m; coll. R/V ‘Velero IV’, station 1944-50, 25 April 1950; SBMNH 422325 [wet]. –1 colony; 4 mi. N of Isla Todos Santos, on rock, 31°52'10"N, 116°49'25"W (end), 73 m; coll. R/V ‘Velero III’, station 1244-41, 24 Feb. 1941; SBMNH 422319 [wet]. **MEXICO, Baja California Sur (Gulf of California)** – 1 fragment; E of Isla San Francisco (Francisquito), sandy mud, 24°47'25"N, 110°31'20"W, 109 m; coll. R/V ‘Velero III’, station 651-37, 9 March 1937; SBMNH 422330 [wet].

**Other Material Examined. USA, California** – San Diego County, San Diego, off Point Loma, 32°35'05"N, 117°20'02"W, 91–101 m, by otter trawl; coll. R/V ‘E.B. Scripps’ (student cruise), A Fleminger, 15 November 1980; SIO-CO 912 [wet]. –1 colony; San Diego County, Canyon Rim, 80 m; coll. R Grigg, Cruise SI 49a, 2 October 1965; (was SIO/BIC CO 1593(b), now SIO-CO 2087(a)); [wet]. –1 fragment; San Diego County, Canyon Rim, 77–80 m; coll. unknown, Cruise SI 50, 02 October 1965; (was SIO/BIC CO 1683(a), now SIO-CO 2088, 2089 or 2090) [wet]. –San Diego County (?), Sea Lab SL 49-d, 80 m; coll. R Grigg, 2 October 1965; SIO-CO 1578 [wet]. –San Diego County, La Jolla, La Jolla Canyon, 47 m; coll. R Ghilardi and party, 4 March 1959; SIO-CO 1685; [wet]. –1 colony; San Diego County; Station 2104, 86 m; det. M Lilly and D Pasko, City of San Diego, MBL, 26 July 1996; [dry]. –1 colony; Los Angeles County, San Clemente Island, NE side, at Forbidden Reef, 33°00'51"N, 118°20'37"W; coll. J Cooper, 11 June 1982; MLML CO 109 [wet]. –Los Angeles County, Santa Monica, 33°52'08"N, 118°34'05"W (end), 73 m; 29 March 1974; SIO-CO 1629 [wet]. –Ventura County, Anacapa Island, south side, 34°00'00"N, 119°26'00"W, 15–26 m; coll. FG Hochberg, 20 November 1964. LACoMNH No. 64-28 [wet].

***Eugorgialjubenkovia* sp. nov.**

**Material examined.** 5 lots; two specimens of species in SBMNH collection from Mexico; another 3 lots, collected by staff of OCSD in California waters, now housed at SBMNH. – fragment; USA, California, Orange County, off Huntington Beach, by Van Veen grab, ~33°36'34"N, 118°02'32"W, 30 m; coll. OCSD, Survey 94–100, station 34, Rep. 1, 1994; material from J Ljubenkov; SBMNH 472234 [wet]. –numerous fragments; USA, California, Orange County, off Huntington Beach, otter trawl, ~33°35'41"N, 117°59'34"W, 35 m; coll. OCSD, Survey 8718, station T-2, Rep. 1, Haul 1, 13 January 1987; Bottle 0011, SBMNH 472232 [wet]. –1 colony + fragment; USA, California, Orange County, off Huntington Beach, otter trawl, at night, ~33°35'57"N, 118°02'47"W, 36 m; coll. OCSD, Survey 8836, station T-6, Rep. 1, 1988; Bottle 0080, SBMNH 472233 [wet]. **MEXICO, Baja California Norte y Sur** – 1 strand; 28 miles south of Punta Abreojos, bearing 199° T from Abreojos Light, 26°17'30"N, 113°41'45"W, 98 m; coll. R/V ‘Velero IV’, station 1709-49, 7 March 1949; SBMNH 472245 [wet]. –colony fragments; Isla Cedros, S Bay, dredge—sand and mud, *Lovena* (heart urchin), sponges, 28°04'21"N, 115°17'50"W (end), 29 m; coll. R/V ‘Velero IV’, station 1703-49, 5 March 1949; SBMNH 422333–**Holotype** [wet].

***Leptogorgiachilensis* (Verrill, 1868)**

**Material examined.** ~25 lots. **USA, California** – 1 colony (partial); Los Angeles County, Farnsworth Bank, dredge—bryozoan, 33°20'39"N, 118°30'55"W (end), 15 m; coll. R/V ‘Velero IV’, 1904-49, 7 September 1949; SBMNH 422940 [wet]. –1+ colony; Los Angeles County, 5 miles, 152° from San Pedro Breakwater, 33°38'20"N, 118°12'00"W, 31–35 m; coll. R/V ‘Velero III’, station 1232-41, 15 February 1941; SBMNH 423066 [wet]. –1+ colony + fragments; Los Angeles County, 2.25 miles south of San Pedro Breakwater, 33°40'30"N, 118°14'20"W, 27 m; coll. R/V ‘Velero III’, station 1207-40, 24 November 1940; SBMNH 423067 [wet]. –two fragments; Los Angeles County, Palos Verdes Estates, 33°47'18"N, 118°24'47"W; coll. T Burch, station 40129, 22 August 1940; SBMNH 422954 [wet]. –multiple fragments; Los Angeles County,7.35 miles, 89° T to Manhattan Beach Pier, 33°53'18"N, 118°32'45"W (end), 58 m; coll. R/V ‘Velero IV’, station 24493-76, 10 March 1976; SBMNH 422943 [wet]. –2 colonies; Ventura County, Anacapa Island, 34°00'14"N, 119°23'41"W, 6 m; coll. unknown, 24 January 1963; with hydroid colony on one branch; SBMNH 422944 [wet]. –1 colony; Ventura County, Anacapa Island, 100 yards out from Cat Rock, 34°00'13"N, 119°25'16"W, 17–18 m; coll. B Scronce and party, 30 October 1962; SBMNH 422945 [wet]. –1 colony; Santa Barbara County, ~6 miles south and west of Summerland coast, on northwest corner of Platform “A”, ~34°20'49"N, 119°38'25"W, ~39 m; coll. S Clark, 3 December 2010; SBMNH 265962 [wet]. –1 colony; Santa Barbara County, Goleta, Sands Beach, 34°24'00"N, 119°53'00"W, ~6 m; coll. R/V ‘Vantuna’ Cruise, 469—Mar. Bio, November 2001; SBMNH 422953–**Neotype** [wet]. **MEXICO, Baja California Sur (Pacific Coast)** – 1 colony + branch fragments; 6.5 miles, 167° T from Punta Abreojos, dredge–coarse mud and sand, mud bottom, 26°35'27"N, 113°31'45"W (end), 51 m; coll. R/V ‘Velero IV’, station 1954-50, 29 April 1950; SBMNH 422947 [wet]. –2 colonies; Canal de Dewey, opposite Punta San Eugenia, coralline, rock, 27°49'40"N, 115°06'20"W (end), 38–47 m; coll. R/V ‘Velero III’, station 1260-41, 27 February 1941; SBMNH 422949 [wet]. –1 colony; 2 miles, 220° T from Punta San Eugenia, 27°49'30"N, 115°05'10"W (end), 38 m; coll. R/V ‘Velero IV’, station 11514-67, 15 June 1967; SBMNH 422950 [wet]. **MEXICO, Baja California Norte (Pacific Coast)** – 1 fragment; South Bay, Isla Cedros, rock, along margin of kelp bed, 28°04'45"N, 115°21'05"W, 18–27 m; coll. R/V ‘Velero III’, 287-34, 10 March 1934; SBMNH 422951 [wet]. –1 colony, incomplete; 6.5 miles SW of Punta San Carlos, dredge–rocky bottom, 29°33'30"N, 115°35'28"W (end), 36 m; coll. R/V ‘Velero IV’, station 1944-50, 25 April 1950; SBMNH 422952 [wet].

**Other material examined. USA, California** – 3 fragments; San Diego County, Canyon Rim, 73 m; coll. R Grigg, Cruise SI 49a, 4 October 1965; SIO/BIC 1593(a) [wet]. –1 strand; San Diego County, Canyon Rim, 70–73; coll. unknown, Cruise SI 50, 2 October 1965; SIO/BIC CO 1683(c) [wet]. –2 lots; colony/fragments; San Diego County, Scripps Submarine Canyon, off Scripps Institute, 15–21 m & 23–30 m; coll. C Limbaugh, December 1953; [Limbaugh Collection, NMNH, dry]. –1+colony/ fragments; San Diego County, La Jolla Canyon, one quarter mile off Scripps Institute, 46 m; coll. C Limbaugh, 23 July 1954; [Limbaugh Collection, NMNH, dry]. –colony/ fragments; San Diego County, one quarter mile off Point La Jolla, edge of kelp bed, 18–21 m; coll. C Limbaugh, 14 December 1953; [Limbaugh Collection, NMNH, dry]. –colony/fragments; San Diego County, 1 mile north of La Jolla, 29 m; coll. C Limbaugh, 11 February 1952; [Limbaugh Collection, NMNH, dry]. –1 colony; Los Angeles County, San Clemente Island, NE side of island, Forbidden Reef, 33°01'26"N, 118°20'17"W; coll. J Cooper, by SCUBA, 11 June 1982; MLML C0108 [wet]. –colony/fragments; Los Angeles County, Redondo Beach, Santa Monica Bay, Streetcar Reef, ~33°51'00"N, 118°25'04"W, 15 m; coll. unknown, 18 July 1961; [Limbaugh Collection, NMNH, dry]. –colony/fragments; Los Angeles County, Redondo Beach, Santa Monica Bay, Streetcar Reef, 18 m; coll. unknown, 14 September 1961; [Limbaugh Collection, NMNH, dry]. –colony/fragments; Ventura County, ~10 miles south of Santa Barbara, California, Richfield Oil Island, 5–9 m; coll. unknown, 16 May 1961; [Limbaugh Collection, NMNH, dry]. –1 colony (possibly this species); Santa Barbara County, S side of Santa Cruz Island, Smuggler's Cove, 34°01'13"N, 119°32'25"W, washed up on cobbles; coll. N Moore, det. EA Horvath, photographed only, 10 March 2008 [dry]. **MEXICO, Baja (Pacific Coast)** – colony/fragments; MEXICO, Baja California Sur, Bahia Tortugas area, ~15–18 m; coll. J Stewart, November 1959; [Limbaugh Collection, NMNH, dry]. –colony/fragments; MEXICO, Baja California Norte, Isla Guadalupe, 42 m; coll. J Stewart and party, February 1960 [Limbaugh Collection, NMNH, dry].

**Other material, not examined** – 1 specimen: USA, California, Santa Barbara County, Santa Cruz Island, Smuggler’s Cove, 21 m.; coll. B Scronce and party, by diving, 24 January 1963; [DH 426 = SBMNH-13 [dry], missing from SBMNH collection.

***Leptogorgiadiffusa* (Verrill, 1868)**

**Material examined.** 4 lots. **MEXICO (Gulf of California)** – 1 colony; Sonora, Cabo Tepoca, 30°15'45"N, 112°53'20"W, on shore–rock and reef; coll. R/V ‘Velero III’, station 1077-40, 4 February 1940; SBMNH 423089 [dry]. –multiple colonies; Sonora, off Rocky Point, mud, sand and shell, 31°19'50"N, 113°39'10"W (end), 18–20 m; coll. R.V ‘Velero III’, station 1072-40, 2 February 1940; SBMNH 423088 [dry]. –1 colony; Sonora, Bahia de Choya (Cholla), ~31°21'19"N, 113°37'26"W; coll./legit. JW with # 038-1, 25 February 1959; SBMNH 423090 [dry].

**Other material examined** –1 colony; USA, California, San Diego County, San Diego, off shore; coll. J Stewart, 1965; CAS IZ 97890 [dry].

***Leptogorgiafilicrispa* Horvath, 2011**

**Material examined.** ~9 lots. **USA, California** – 2 fragments (1 lot); Ventura County, off Ventura Harbor, collected with ovulid snails, 34°14'00"N, 119°19'00"W, 20–50 m; coll. P Brophy, trawled by dragnets, August 1968; SBMNH 423079 [housed in vial, in ovulid snail collection, *Neosimnia* nec *Simnialoebbeckeana*, SBMNH 423103, dry]. **MEXICO, Baja California Sur (Pacific Coast)** – multiple colonies (1 lot); off Boca Flor de Malva, SE of Punta Tosca, 24°11'07"N, 111°21'03"W, 69–87 m; coll. J McLean on R/V ‘Searcher’, Station 31–32, No. 71–16, 1 February 1971; SBMNH 423057–**Holotype** [DH 415 = SBMNH-06, dry].

**Other material examined. MEXICO (Gulf of California)** – several fragments; Gulf of California, Sonora, Mexico, Guaymas, 28°15'00"N, 111°48'00"W (end), 64–75 m; coll. unknown, 21 March 1960; USNM 75099 [wet]. –numerous fragments/colonies; Sonora, Cabo Tepoca, 30°20'30"N, 113°08'00"W, 56–67 m; coll. R Parker, 31 March 1960; (with a station number: P-212-60); USNM 1106683 [dry]. **MEXICO, Baja California Sur (Pacific Coast)** – multiple fragments/colonies; Cabo San Lucas Canyon, ~22°54'09"N, 109°22'45"W, on sand bottom, 30 m; coll. W North and C Limbaugh, March 1959; USNM 1106685 [dry]. –multiple fragments; off Boca Flor de Malva, SE of Punta Tosca, 24°11'07"N, 111°21'03"W, 69–87 m; coll. J McLean on R/V ‘Searcher’, Station 31–32, No. 71–16, 1 February1971; LACoMNH—**Holoparalectotype** [DH 218, SBMNH06, dry]. –multiple fragments; off Boca Flor de Malva, SE of Punta Tosca, 24°11'07"N, 111°21'03"W, 69–87 m; coll. J McLean on R/V ‘Searcher’, Station 31–32, No. 71-16, 1 February1971; LACoMNH—**Holoparalectotype** [DH 415, SBMNH06, dry]. –multiple fragments; Cabo San Lazaro, 24°48'00"N, 112°19'12"W, by shrimp trawler, 65–73 m; coll. C Limbaugh, sometime in the 1950s; USNM 1106684 [dry]. –several fragments; Magdalena Bay, ~24°38'11"N, 111°58'15"W, 86 m; coll. unknown on R/V ‘Albatross’, 8 April 1889; USNM 57144 [wet]. –multiple fragments; SW Punta San Juanico, outer coast, ~26°12'33"N, 112°30'49"W, (no depth recorded); coll. unknown, 29 January 1971; LACoMNH [DH240, dry].

***Leptogorgiaflexilis* (Verrill, 1868)**

**Material examined.** 5 lots. **USA, California** – 1 colony fragment (base missing); Los Angeles County, San Pedro, 6.9 miles, 139° T from Point Fermin, dredge–sand, 33°37'04"N, 118°11'50"W (end), 45 m; coll. R/V ‘Velero IV’, 2042-51, 20 July 1951; SBMNH 422941 [wet]. –2 lots; colony, fragmented; Los Angeles County, between White's Point and Portuguese Bend, 33°42'54"N, 118°20'07"W (end), by beam trawl, 22 m; coll. R/V ‘Velero IV’, station 1644-48, 19 November 1948; SBMNH 422942 [dry & wet]. **MEXICO, Baja California Sur (Pacific Coast)** – fragments; 28 miles, 199° T from Abreojos Light, S of Punta Abreojos, dredge–brachiopods, beam trawl–# 12 coarse sand and rock, 26°17'15"N, 113°41'45"W (end), 100 m; coll. R/V ‘Velero IV’, station 1709-49, 7 March 1949; SBMNH 422946 [wet]. –1 colony + multiple fragments; 2 miles, 142° T from Thurloe Head, 27°35'45"N, 114°49'15"W, no depth recorded; coll. R/V ‘Velero IV’, station 11842-67, 7 December 1967; SBMNH 422948 [wet].

***Leptogorgia* sp. A (? = *Leptogorgiatricorata*Breedy and Cortés 2011)**

**Material examined.** ~10-11 lots. **USA, California** – 2 colonies, 1 fragment; 9.5 miles NW of buoy, Cortes Bank, white sand, rock, 32°33'15"N, 119°15'15"W, 91 m; coll. R/V ‘Velero III’, station 1342-41, 10 June 1941; SBMNH 423080 [wet]. –4 colonies; Los Angeles County, Santa Catalina Island, 1 mile SW of Ben Weston Point, mud, sand, gravel, 33°20'55"N, 118°30'45(35)” W (end), 82–89 m; coll. R/V ‘Velero III’, station 1316-41, 17 May 1941; SBMNH 422334 [wet]. –1 colony; Los Angeles County, Santa Catalina Island, Bird Rock, ~33°27'05"N, 118°29'10"W, 46 m; coll. R Given, July 1977; SBMNH 423083 [wet]. –1 fragment; Los Angeles County, Santa Catalina Island, off Bird Rock, rock, coarse shell, kelp; sm. dredge boat, 33°27'20"N, 118°29'00"W (end), 56–73 m; coll. R/V ‘Velero III’, station 1187-40, 29 September 1940; SBMNH 423081 [wet]. –1 fragment; Ventura County, Santa Barbara Island, N of island, 33°30'58"N, 119°00'50"W, on gray sand, 67-73 m; coll. R/V ‘Velero III,’ station 1177-40, 9 September 1940; SBMNH 422903 [wet]. –2 colony fragments; Los Angeles County, off Rocky Point, close in proximity to Point Vicente (S end of Santa Monica Bay), ~33°44'33"N, 118°25'28"W; coll. C Limbaugh, 5 October 1941; SBMNH 423082 [wet]. –numerous colonies/fragments; 10.75 miles W of Point Dume, on loose rock and sponge, 34°00'15"N, 119°01'30"W (end), 86-87 m; coll. R/V ‘Velero III’, station 1276-41, 23 March 1941; SBMNH 422340 [wet]. –fragment; Santa Barbara County, Channel Island area, ~34°06'56"N, 119°42'49"W, by trawl; coll. P Brophy; SBMNH 423084 [wet].

**Other material examined** – fragment; Mexico, Baja California Norte (Gulf of California), NE of Isla San Jose, Isla Las Animas, 28°42'14"N, 112°55'43"W, epizooic on “black coral, 36–42 m; coll. Adcock and Markham, Station D-24B, 11 August 1965 (identified in error by Harden as *Heterogorgiatortuosa*); CAS-IZ 96757 [wet]. –1 colony; USA, California, San Diego County, near coast of San Diego, no depth recorded; coll. J Stewart (UCSD diver), 1965 (identified in error by Harden as *H.tortuosa*); CAS-IZ 98140 [dry]. –1 strand; USA, California, San Diego County, Canyon Wall (1200), 82 m; coll. R Grigg, Cruise SL 49c, 2 October 1965; SIO-CO 1594 [wet]. –colony fragment; USA, California, Ventura County, Channel Islands, 10 miles E of San Nicolas Island, ~33°12'19"N, 119°43'29"W, 91 m; coll. (possible ‘Albatross’ collection, according to Harden; identified in error as *Heterogorgiapapillosa*), 29 March 1917; CAS-IZ 34636 [wet].

**Other material, not examined** – Japan, Shizuoka, Honshu Island, Suruga Bay, Omae Zaki, 34°35'00"N, 138°15'00"E; coll. R/V ‘Albatross’, station nos. 3727-3735, US Fish Commission, 16 May 1900, 62–89 m; USNM 50248 [wet].

***Chromoplexauramarki* (Kükenthal, 1913)**

**Material examined.** ~60 lots. **USA, California** – (?)1 colony; Ventura/Los Angeles County, 20–22 miles south of San Nicolas Island, rocky, 32°51'00"N, 119°23'45"W, 118–136 m; coll. R/V ‘Velero III’, 1344-41, 11 June 1941; SBMNH 423071 [wet]. –4 fragments (2 lots); Los Angeles County, Santa Catalina Island, 5 miles SE of Church Rock, 33°16'00"N, 118°13'45"W (end), 215 m on sand, loose washed rock; coll. R/V ‘Velero III’, Station 1350-41, 12 June 1941; SBMNH 265946 [wet]. –multiple fragments/colonies (two lots); Los Angeles/Ventura County, off San Nicolas Island, with sponge, rock, 33°16'10"N, 119°24'30"W (end), sponge and rock, 51–56 m; coll. R/V ‘Velero III’, 1123-40, 12 April 1940; SBMNH 423061 [wet]. –3 colonies, 1 fragment; Los Angeles County, 2.5 miles SE of Seal Rocks, Santa Catalina Island, 33°17'20"N, 118°15'35"W (end), rock, lumpy gray sand, sponge, urchin, gorgonians, hermits, 158-173 m; coll. R/V ‘Velero III’, station 1429-41, 25 October 1941; SBMNH 265948 [wet]. –1 colony; Los Angeles County, 5 miles, 152° T from San Pedro Breakwater, 33°38'20"N, 118°12'10"W (end), coarse sand, dead shell, clay, 31–35 m; coll. R/V ‘Velero III’, 1232-41, 15 February 1941; SBMNH 423066 [wet]. –1 colony; Los Angeles County, 2.25 miles south of San Pedro Breakwater, 33°40'30"N, 118°14'20"W (end), sand, pebble, broken shell, 27 m; coll. R/V ‘Velero III’, 1207-40, 24 November 1940; SBMNH 423067 [wet]. –multiple fragments (bearing numerous brittle stars); Santa Barbara County, Santa Cruz Island, 13 miles, 5° T to Gull Island Light, 33°44'00"N, 119°51'00"W, 155 m; coll. R/V ‘Velero IV’, station 24329-76, 21 January 1976; SBMNH 265935 (with label reading: 24329 BFI) [wet]. –fragment; Santa Barbara County, 16.5 miles SE X S of South Point, Santa Rosa Island, 33°38'35"N, 119°58'05” (end) W, rocks, crinoids, brittle stars, 131–140 m; coll. R/V ‘Velero III’, station 1397-41, 27 August 1941; SBMNH 265936 [wet]. –1 colony; Santa Barbara County, Santa Rosa Island, 6.27 miles, 341° T to Ford Point, 33°48'45"N, 119°59'20"W (end), 104 m; coll. R/V ‘Velero IV’, station 23065-75, 18 October 1975; SBMNH 265937 (with label reading: 23065 6C) [wet]. –1 colony; Santa Barbara, County, San Miguel Island, 3.7 miles, 21° T to Crook Point, 33°56'48"N, 120°21'42"W (end), 118 m; coll. R/V ‘Velero IV’, station 24882-76, 28 April 1976; SBMNH 265949 (with label reading: 24882 CH) [wet]. –1 colony; Santa Barbara County, south side of Santa Cruz Island, gray sand, shell, 33°57'45"N, 119°38'20"W (end), 67–73 m; coll. R/V ‘Velero III’, 1191-40, 30 September 1940; SBMNH 423068 [wet]. –1 colony; 2.6 miles, 140° T to Cavern Point, Santa Cruz Island, 34°04'30"N, 119°34'18"W (end), 76 m; coll. R/V ‘Velero IV’, station 24863-76, 27 April 1976; SBMNH 423064 [wet]. –1 colony (fragments); Ventura County, 1 mile, 231.5° T from Ventura Pier Light, 34°10'00"N, 118°26'57"W, no depth; coll. R/V ‘Velero IV’, station 5828-58, 21 August 1958; SBMNH 265947 [wet]. –(?)1 colony; Monterey County, off San Jose Creek Beach (N end), Carmel Submarine Canyon, 36°32'00"N, 121°56'00"W, 12–38 m; coll. J H McLean; 1960-1964; SBMNH 423070 [wet] (formerly part of Los Angeles County Museum collection). –1 small colony; Monterey County, Point Sur, 36°17'02"N, 121°57'09"W, 56 m, on large rock; coll. D Starr, NMFS-FED, Santa Cruz, 13 October 2007; SBMNH 235536 [dry]. –1 fragment; Monterey County, southwest of Point Pinos, 36°37'25"N, 121°58'25"W, rock and sponges, 47 m; coll. R/V ‘Velero III’, 891-38, 8 August 1938; SBMNH 423062 [wet]. –1 colony; Monterey County, Monterey Bay, 1.15 miles, 347° T from Point Pinos Light, on rock, 36°38'54"N, 121°56'15"W; coll. R/V ‘Velero IV’, station 6437-59, 30 September 1959; SBMNH 423065 [wet]. –fragments; Monterey County, Monterey Bay, off Point Pinos, rock, crinoids, brittle stars, 36°39'50"N, 121°58'00"W, 89–98 m; coll. R/V ‘Velero III’, station 890-38, 8 August 1938; SBMNH 423063 [wet]. –1 colony; Monterey County, Monterey, 36°47'00"N, 122°02'00"W, ~22 m; coll. T Burch, BLM Ref. Sta. 360, 18 August 1940, with label (Burch # 40128); SBMNH 423060–**Neotype** [dry]. –1 colony + fragments; Monterey County, Monterey, 36°47'00"N, 122°02'00"W, ~22 m; coll. T Burch, possible BLM Ref. Sta. 360, 18 August 1940, with label (Burch # 40128); SBMNH 423072 [wet]. –1 colony; Monterey County, Monterey, 36°47'00"N, 122°02'00"W, ~30–45 m; coll. T Burch (Acc. No. 3665), BLM Ref. Sta. 360, 18 August 1940; SBMNH 422954 [wet]. –1 colony; Santa Cruz County, Santa Cruz, 36°57'07"N, 122°00'36"W, 30 m; coll. Kinnetics Lab; Station 1, 7 July 1978; with barnacles growing on the colony, where some branches come off of main “stem;” SBMNH 423069 (previous no. 40612, not in Data Base; specimen originally labeled *Psammogorgiatorreyi*). [wet]. **USA, Oregon** – 1 colony plus fragments; Lincoln County, 15 nautical miles off coast of Newport, Oregon, Station NH-15, 44°39'28"N, 124°24'29"W, 85 m; coll. R/V ‘Yaquina’, Cruise Y6906C, 27 June 1969; SBMNH 423073 [wet].

**Other material examined. USA, California** – multiple fragments; San Luis Obispo County, 27° off Avila Beach, starting just N of bell buoy, around Souza Rock, ~35°10'35"N, 120°44'16"W, 36 m; coll. R/V ‘NB Scofield’, Field No. 53-B-25, 9 June 1953; CAS-IZ-96742 [wet]. –1 colony; San Luis Obispo County, ~10 miles S of Morro Bay, near Point, ~35°22'01"N, 121°02'55"W, 9 m; coll. abalone diver via scuba (“Ed”), 1964; CAS-IZ 97951 [dry]. –1 colony; Monterey County, between Point Lobos and Sabrina Point, ~36°31'28"N, 121°57'03"W, 364 m; coll. Capt. Alioti, on Shrimper ‘Jackie Boy’ (lot 2), 4 March 1972; CAS-IZ 96735 [wet]. –1 colony; Monterey County, Carmel Bay, off San Jose Creek Beach (Monastery Beach), ~36°31'34"N, 121°55'50"W, 38 m; coll. D Sullivan, 20 May 1962; CAS-IZ 96746 [wet]. –1 colony + 1 fragment; Monterey County, Monterey Bay, bearing S 25° E, 5.4 miles off Point Piños lighthouse, ~36°35'49"N, 122°00'13”W 170 m; coll. USBCF ‘Albatross’, station 4543, Haul # 816, 1904 SIO/BIC CO 361 [wet]. –1 colony/fragments; Monterey County, Monterey Bay, bearing N 87° W, 1.74 miles off Point Piños lighthouse, ~36°38'30"N, 121°57'45"W, 64 m; coll. USBCF ‘Albatross’, station 4441, 1904SIO/BIC CO 1810 [wet]. (Both SIO/BIC CO 361 and CO 1810, are labeled *P.arbuscula*; this is in error, based on location data.) –1 colony; Monterey County, Monterey Bay, Monterey Bay Canyon Wall, ~36°52'37"N, 121°56'50"W, 500–600 m; coll. C Mah, R/V ‘Point Sur’, 28 February 1997; CAS-IZ 108905 [wet]. –2 colonies; Monterey County, Monterey Bay, Continental Shelf, ~36°52'37"N, 121°56'50"W, 150–160 m; coll. C Mah, R/V ‘Point Sur’, 14 April 1998; CAS-IZ 113071 [wet & dry]. –colonies; Monterey County, Monterey Bay, Monterey Canyon, canyon wall, ~36°52'37"N, 121°56'50"W, 600–700 m; coll. C Mah, R/V ‘Point Sur’, 14 April 1998; CAS-IZ 113072 [wet]. –1 colony; San Francisco County, ? Farallon Islands, ~37°44'22"N, 123°03'13"W, no depth recorded; coll. unknown, with exception of “Trawl no. PC1-1: sample no. 1,” 23 September 1991; CAS-IZ 96747 [wet]. –1 colony; Humboldt County, Blunt's Reef, off Cape Mendocino, California, ~40°25'56"N, 124°27'28"W; coll. Turkington, May 1910; det. by Bayer; USNM 51500 [dry]. **USA, Oregon and Washington** – 1 colony; no specific location data, 600 m; coll. A Carey Jr., R/V ‘Acona’, Station OTB-36 16 June 1964; CAS-IZ 143903 [wet]. –1 colony and 1 fragment; Coos County, off coast, Coquille Bank, ~42°54'23"N, 124°51'16"W, 257 m; coll. P Etnoyer, NOAA “West Coast Survey, Fall 2010, 3 November 2010; BS004 (CB 50001-002) and BS005 (CB 50001-003) [wet]. –fragment; off Oregon coast, Heceta Bank, 43°55'58"N, 124°55'55"W, 140 m; coll. Heceta RB-00-05, G Hendler; RV ‘Ronald H. Brown’ (NOAA) and S/V ‘Ropos’, Dive R534-Rk-009, 24 June 2000; LACoMNH Marine Biodiversity Center processing number 233 [wet]. –fragment; off Oregon coast, Heceta Bank, 43°56'52"N, 124°56'36"W, 189 m; coll. Heceta RB-00-05, G Hendler; RV ‘Ronald H. Brown’ (NOAA) and S/V ‘Ropos’, Dive R535-Bio-2, 3, 4, or 13, 24 June 2000; LACoMNH, Marine Biodiversity Center processing number 379 [wet]. –1 colony; off Oregon coast, Heceta Bank, 43°58'07"N, 124°51'10"W, hard bottom covered with invertebrates, 117.9 m; coll. A Valdés by ROV; RV ‘Ronald H. Brown’ (NOAA) and S/V ‘Ropos’, Dive 615, 10 July 2001; LACoMNH, Marine Biodiversity Center processing number 100 [wet]. –1 colony; off Oregon coast, Heceta Bank, 43°59'16"N, 124°52'59"W, rocky area, collected with a large rock, 81.1 m; coll. A. Valdés by ROV; RV ‘Ronald H. Brown’ (NOAA) and S/V ‘Ropos’, Dive 613, 9 July 2001; LACoMNH Marine Biodiversity processing center number 85 [wet]. –1 colony; off Oregon coast, Heceta Bank, 44°09'11"N, 124°49'29"W, 81 m; coll. Heceta RB-00-05, G Hendler; RV ‘Ronald H. Brown’ (NOAA) and S/V ‘Ropos’, Dive R530-Bio-0012, 21 June 2000; LACoMNH, Marine Biodiversity Center processing number 205 [wet]. –1 colony; off Oregon coast, Heceta Bank, 44°11'08"N, 124°48'24"W, rocky bottom, collected with rock, 74.3 m; coll. R Embley by ROV; RV ‘Ronald H. Brown’ (NOAA) and S/V ‘Ropos’; Dive 608, 7 July 2001; LACoMNH, Marine Biodiversity Center processing number 56 [wet]. –2 colonies; off Oregon coast, Heceta Bank, 44°11'14"N, 124°50'03"W, rocky bottom, collected with rock with gorgonian attached, 81.8 m; coll. N Puniwai by ROV; RV ‘Ronald H. Brown’ (NOAA) and S/V ‘Ropos’, Dive 608, 7 July 2001; LACoMNH, Marine Biodiversity Center processing number 55 [wet]. –1 colony; off Oregon coast, Heceta Bank, 44°13'20"N, 124°52'26"W, 98 m; coll. Heceta RB-00-05, G Hendler; RV ‘Ronald H. Brown’ (NOAA) and S/V ‘Ropos’, Dive R538-Rk-0003, 26 June 2000; LACoMNH Marine Biodiversity Center processing number 263 [wet]. –2 colonies; Lincoln County, off Newport, ~44°40'10"N, 124°27'12"W, 110 m; coll. Dr JE McCauley, R/V ‘Acona’, no collection date given; CAS-IZ 24639 [wet]. –2 colonies + fragments; Lincoln County, 112.01 miles SW of Yaquina Head Light, 44°40'00"N, 126°21'12"W, 2,856 m; coll. A Carey, OSU, Station OTB-30, 20 May 1964; SBMNH 423078 [wet]. –1 colony; Eastern N Pacific, Tillamook County, off Oregon coast, due W of Nehalem Bay State Park, 45°38'55"N, 124°06'03"W, ~80 m; coll. NFSC, 2006 expedition; CB 34213-093, FRAM 100105460 [wet]. –1 colony, North Pacific, off Oregon/Washington border, Clatsop County, due W of Tillamook Head, and mouth of Columbia River, ~46°13'30"N, 124°22'31"W, ~113 m; coll. NFSC, 2006 expedition; CB 34210-023, FRAM 100105455 [wet]. –3 colonies; North Pacific, Grays Harbor County, off Washington State, due W of Quinault Beach Resort, ~47°01'33"N, 124°39'10"W, ~98 m; coll. NFSC, 2006 expedition; CB 34210-025, FRAM 00105453 [wet]. –1 colony, North Pacific, off Washington coast, Grays Harbor County, ~47°06'07"N, 124°32'17"W, 73 m; coll. NFSC, WCGS 2006; CB 34210-020, FRAM 100105450 [wet].

**Other material, location not known.** WCGS 2006, Oregon – CB 34213-047, FRAM 100105469 (provided by E Berntson, NOAA Fisheries Office, Port Orchard, WA).

**Other material, not examined** – several colonies; USA, N Pacific Ocean, California, Monterey County, Carmel, 26 m; [YPM 8877A and YPM 8877B; notation of “questionable form,” wet].

***Muriceacalifornica* Aurivillius, 1931**

**Material examined.** ~33 lots. **USA, California** – (?)1 colony; Orange County, Newport Bay, 33°35'37"N, 117°52'51"W, on rocks of outer jetty, below extended low tide; coll. G MacGinitie, October 1946; SBMNH 422390 [wet]. –multiple colonies/fragments; Orange County, Newport Beach, “The Pipeline,” ~33°36'23"N, 117°55'50"W, depth not recorded; coll. Aquarium of the Pacific, Long Beach (for SBMNH Sea Center), early summer, 2010; SBMNH 265938 [wet]. –1 colony; Orange County, Newport Bay, 33°37'11"N, 117°53'41"W, depth not recorded; coll. G Sphon, 08 January 1953; SBMNH 422920 [DH 451 = SBMNH-35; dry]. –2+ colonies; Orange County, off Huntington Beach, with a beam trawl, 33°38'30"N, 118°00'20"W, 7–36 m; coll. R/V ‘Velero III’, 1127-40, 20 April 1940; SBMNH 422360 [1 dry, others wet]. –1 colony; Los Angeles County, San Pedro, Point Fermin, 33°41'00"N, 118°16'00"W; coll. R/V ‘Velero IV’, station 1578-46, 20 December 1946; data from label, not Station Log (NOTE: from August 1942-July 1948, no Station Log notes; ship not running consistently during WWII?); SBMNH 422361[wet]. –fragment; Los Angeles County, San Pedro, inside of Point Fermin, on shore–rock wall and loose rock, 33°42'45"N, 118°16'45"W; coll. R/V ‘Velero III’, station 1217-40, 30 November 1940; SBMNH 422357 [wet]. –multiple colonies; Los Angeles County, San Pedro, Point Fermin, 33°42'18"N, 118°17'35"W, 13 m; with data station number: 50 3 T147, 17 June 1916; SBMNH 422375[wet]. –several colonies in 3 lots; Los Angeles County, San Pedro Breakwater, on shore, 33°42'08"N, 118°16'05"W; coll. R/V ‘Velero III’, station 1230-41, 26 January 1941; SBMNH 422358 [wet]. –1 colony; Los Angeles County, between White's Point and Portuguese Bend, beam trawl–bryozoa, corals, sea mouse, gorgonacea, 33°42'54"N, 118°20'07"W (end), 22 m; coll. R/V ‘Velero IV’, station 1644-48, 19 November 1948; SBMNH 422359 [wet]. –1 colony; Los Angeles County, between White's Point and Portuguese Bend, 33°42'54"N, 118°20'07"W, 22 m; coll. R/V ‘Velero IV’, station 1644-48, 19 November 1948; SBMNH 422919 [dry]. –1 colony (stripped); Ventura County, Rincon Beach, 30°20'10"N, 119°24'35"W, on beach; coll. N Moore, 2 March 2008; SBMNH 422918 [dry]. –1 colony; Santa Barbara County, Anacapa Island, 34°00'13"N, 119°25'16"W, 100 yards out from Cat Rock, 17–18 m; coll. B Scronce, and party, 30 October 1962; SMBNH 45564; [wet]. –1 colony; Santa Barbara County, Anacapa Island, 34°00'13"N, 119°25'16"W, 100 yards out from Cat Rock, 17–18 m; coll. B Scronce, and party, 30 October 1962; SBMNH 45563; [wet]. –1 colony; Ventura County, Port Hueneme, off the north jetty, 34°09'26"N, 119°13'42"W, 11 m; coll. M Conboy and D Sprong, 16 January 1963; SBMNH 422911 [DH 448 =SBMNH-32; dry]. –3 colonies; Ventura County, Port Hueneme, 34°09'26"N, 119°13'42"W, 11 m; coll. M Conboy and D Sprong, 16 January 1963; SBMNH 422913 [DH 449 =SBMNH-33, dry]. –2 colonies; Santa Barbara County, Carpinteria, several miles east of Carpinteria State Beach, 34°23'36"N, 119°31'31"W, between splash and high inter-tidal, up on rocks and exposed beach; coll. EA Horvath, between 27–30, January 2006; SBMNH 422372 & 422373 [wet]. –2 colonies; Santa Barbara County, Carpinteria, off Carpinteria State Beach, 34°23'36"N, 119°31'31"W; coll. C Watters and J Henry, det. EA Horvath; 18 February 2008; SBMNH 422914 [dry]. –1 fragment; Santa Barbara County, Santa Barbara, Naples Reef, 34°26'24"N, 119°56'60"W, 15 m; coll. S Anderson and party, 11 November 1998; SBMNH 345324; [wet]. **MEXICO, Baja California Sur (Pacific Coast)** – 2 colonies + fragments; Cabo San Lucas, 22°52'49"N, 109°54'32"W; coll. unknown, 22 March 1936; SBMNH 422921 [dry]. –1 colony; off Thurloe Head, on rock with gorgonids, 27°36'50"N, 114°50'50"W, 15–18 m; coll. R/V ‘Velero III’, station 283-34, 9 March 1934; SBMNH 422369 [wet]. **MEXICO, Baja California Norte (Pacific Coast)** – 1 fragment; Isla Cedros, South Bay, on rock along margin of kelp bed, 28°04'45"N, 115°21'05"W, 18–27 m; coll. R/V ‘Velero III’, station 287-34, 10 March 1934; SBMNH 422363 [wet]. –2 colonies; Baja, 6.5 miles SW of Punta San Carlos, dredge–rocky bottom, 29°33'30"N, 115°35'28"W (end), 36 m; coll. R/V ‘Velero IV’, station 1944-50, 25 April 1950; SBMNH 422362 [wet]. **MEXICO, Baja California Norte (Gulf of California)** – 2 colonies; Roca Consag, 31°12'17"N, 114°33'39"W; coll. L Findley, 9 July 1973; Nos. 207/208, University of Arizona; SBMNH 422376 [wet]. **MEXICO (Gulf of California)** – 1 colony; Sonora, South of Isla Tiburon, sand, shell, 28°43'45"N, 112°17'50"W, 36 m; coll. R/V ‘Velero III’, 566-36, 11 March 1936; SBMNH 422384 [wet]. **SOUTH AMERICA** – 1 fragment; Ecuador, off Cape San Francisco, 00°37'10"N, 80°00'30"W, 27 m; coll. R/V ‘Velero III’, station 850-38, 23 February 1938; SBMNH 422427 [dry].

**Other material examined. USA, California** – San Diego County, La Jolla, Quast Rock, 21 m; coll. R Kiwala, by scuba, 7 April 1969; SIO/BIC CO 1600 [wet]. –San Diego County, La Jolla, Quast Rock, 20 m; coll. R Grigg, December 1967; SIO/BIC CO 1632. –San Diego County, La Jolla, Quast, 20 m; coll. unknown, October 1966; SIO/BIC CO 1641. –1 lot; San Diego County, Quast Rock, 65 feet; coll. unknown, 29 October 1965; SIO/BIC CO 1945 [wet]. –2 colonies/fragments; Orange County, just south and east of Huntington Beach, ~33°36'50"N, 117°59'13"W, 18 m; coll. OCSD, station T0, Haul 1, 29 February 2012 [wet]. –Los Angeles County, off San Clemente Island, 32°35'29"N, 118°19'02"W, near Station # 2, in restricted area, by SCUBA; coll. J Cooper, 11 June 1982; MLML C0110 [wet]. –colonies/fragments; Los Angeles County, Santa Catalina Island, just outside Blue Cavern Marine Protected Area, near “The Quarry,” ~33°25'53"N, 118°26'49"W, depth unknown; coll. Aquarium of the Pacific, Long Beach, early summer, 2010 [wet]. –Los Angeles County, Coast Station, Palos Verdes, off Marineland of the Pacific, 33°43'58"N, 118°25'12"W, 10–15 m; large boulders with sand between; coll. R Grigg, 7 January 1967; SIO/BIC CO 1596. **MEXICO** – Baja California Norte, Punta Banda, station 2 (no other data); 20 July 1968; SIOBIC CO 1943 “a1-a4”, and probably “b” as well.

All lots SIO/BIC CO 1860 – CO 1939; most of these lots probably this species, a few mixed in likely *M.fruticosa*, but no specific collection location data is readily available. In the “Limbaugh” Collection housed at NMNH, other collection locations could include: (USA) the Richfield Oil Island (Ventura County, ~10 miles south of Santa Barbara, California), Newport Bay (California), in the Huntington Beach Gas and Electric Steam Plant discharge pipe (California), and from Baja: Gulf of Punta Final, Turtle Bay and off Cape San Lucas. There are SIO/BIC CO 1943 and CO 1945, but these are not included in any official listing, and there appeared to be no data to go with the numbers.

**Other material, not examined** (but photographed) or examined/photographed (not collected) – 1 colony; N Pacific Ocean, USA, California, Los Angeles County, San Clemente, ~33°25'21"N, 117°37'22"W, on the beach; photographer A Nelson, det. EA Horvath; 1 March 2008. –1 colony; N Pacific Ocean, USA, California, Ventura California, Santa Barbara Channel Islands, Anacapa Island, ~34°00'19"N, 119°24'55"W, big tidal pool; photographer N Downend, det. EA Horvath; 14 April 2008.

***Muriceaplantaginea* (Valenciennes, 1846) [= *M.appressa* Verrill, 1864**]

**Material examined.** ~4 lots. **MEXICO, Baja California Sur (Pacific Coast)** – 1 colony; Bahia de Santa Maria, 24°42'20"N, 112°14'10"W, 58 m; coll. R/V ‘Velero III’, station 760-38, 5 January 1938; SBMNH 422418; [dry]. –1 colony; Punta Asunción, 27°07'28"N, 114°17'48"W, 7 m; coll. RH McPeak, 15 October 1980; SBMNH 422417 [dry]. **MEXICO, Baja California Norte (Gulf of California)** –1 colony; Isla Partida, off White Rock, 28°55'30"N, 113°05'35"W, 82 m; coll. R/V ‘Velero III’, station 557-36, 8 March 1936; SBMNH 422416 [dry]. **MEXICO (Gulf of California)**

–1 colony; Sonora, Guaymas, Ensenada Carrizal, 27°52'12"N, 110°51'00"W; coll. P LaFollette; August 1960; SBMNH 422912 [DH 450 =SBMNH-34; Also: # 0413, and A339, dry].

**Other material, not examined.**USNM 52301 and USNM 52303 were both collected in the N Pacific, USA, California, Los Angeles County, off San Pedro; no other data given. A third, USNM 94678, may also be this form, as it is from the same collection location. Additionally, from the Limbaugh collection of specimens housed at NMNH, locations for possible examples of this species include: Baja California Sur (Cabo San Lucas; Cape San Lucas Canyon; Bahia de Los Angeles); Baja California Norte (an area near South Cedros Island; perhaps Punta Final and Turtle Bay) and USA, California, San Diego County, La Jolla (Scripps submarine canyon). Further sclerite examinations need to be made.

***Muriceafruticosa* Verrill, 1868**

**Material examined.** ~14 lots. **USA, California** – 1 colony (fragmented); Orange County, Newport Channel, Balboa, shore collected on a –1.6 tide, 33°36'02"N, 117°52'50"W; coll. R/V ‘Velero III’, station 1224-41, 25 January 1941; SBMNH 422383 [wet]. –1 fragment (albino form or bleached–FG Hochberg); Los Angeles County, Santa Catalina Island, Salta Verde Point, 15 m, 33°19'00"N, 118°25'20–27"W; coll. R Given, 1 May 1967; SBMNH 422389 [wet]. –several colonies; Los Angeles County, Santa Catalina Island, Big Fisherman's Cove, 33°20'40"N, 118°29'07"W, 3 m; coll. R Setzer, 22 August 1968; SBMNH 422374 [wet]. –1 colony; Los Angeles County, Palos Verdes, east of Portuguese Bend, 33°43'00"N, 118°19'57"W, 15 m; coll. unknown, 6 November 1949; SBMNH 422428 [dry]. –1 colony; Los Angeles County, Long Beach, Long Beach Harbor, Back Channel, just east of Terminal Island, 33°45'41"N, 118°13'07"W, 7.6 m; coll. DB Cadien, rocky subtidal station I/S7, 31 March 1980; SBMNH 265945 [wet]. –1 fragment; Los Angeles County, Santa Monica Bay, ~33°56'07"N, 118°27'59"W; coll. Aquarium of the Pacific, Long Beach (for SBMNH Sea Center), early summer, 2010; SBMNH 265940 [wet]. –2 colonies (1 incomplete); Santa Barbara County, Santa Cruz Island, Smugglers Cove, 34°01'19"N, 119°32'31"W, 6 m; coll. B Scronce and party, 24 January 1963; SBMNH 422430 [DH 477 = SBMNH-31, dry]. –1 fragment; Santa Barbara County, Mohawk Reef, 34°23'40"N, 119°43'48"W, 4 m; coll. S Anderson, by hand, SCUBA, 01 November 1998; SBMNH 345325 [wet]. **MEXICO, Baja California Sur (Pacific Coast)** –1 colony; Escondido Bay area, Isla Danzante to Punta Candeleros, 25°47'15"N, 111°15'30"W; coll. R McPeak, 30 March 1981; SBMNH 422429; [dry]. –1 colony; 2 miles, 142° T from Thurloe Head, 27°35'45"N, 114°49'15"W, no depth reported; coll. R/V ‘Velero IV’, station 11842-67, 7 December 1967; SBMNH 422910; [wet]. –fragment; Scammons Lagoon, Isla Piedras, high littoral under flat sandstone, 27°46'00"N, 114°15'20"W; coll. W Williams on Kenyon-Williams Expeditions, station M12, 30 April 1946; SBMNH 422387 [wet]. **MEXICO, Baja California Norte (Pacific Coast)** – 1 colony; Baja, 6.5 miles SW of Punta San Carlos, dredge–rocky bottom, 29°33'30"N, 115°35'28"W (end), 36 m; coll. R/V ‘Velero IV’, 1944-50; 25 April 1950; SBMNH 422386 [wet]. **MEXICO (Gulf of California)** – 1 colony; Sonora, Guaymas, “Bahia de Conos” (El Carrisito), 27°52'00"N, 110°54'15"W, subtidal, less than 9 m, attached to rocks; coll. P LaFollette, 8-12 August 1960; SBMNH 422916 [DH 452 =SBMNH-36; ? 0336; ? A332, dry]. –1 colony/2 fragments; Sonora, Guaymas, “Bahia de Conos” (El Carrisito) 27°52'00"N, 110°54'15"W, less than 9 m, attached to rocks; coll. P LaFollette, 5–12 August 1960; SBMNH 422917 [DH 455 =SBMNH-39; ? A20; ? A332; ?0336, dry]. –4 colonies, 2 other fragments; Sonora, Guaymas, Ensenada Carrizal, 27°52'12"N, 110°51'00"W; coll. P LaFollette, 5–13 August 1960; SBMNH 422915 [DH 453 =SBMNH-37; ? PIL-A19; ? A332; ? 0335, dry].

**Other material examined. USA, California** – colony; Dana Point, N of, 33°32'41"N, 117°48'36"W, 48–53 m; coll. T Matsui, with otter trawl on R/V ‘Agassiz’, 29 March 1974; CO/SIO 1628 (M-15, OT-8) [wet/dry?]. –CO/SIO 1939 also identified as this species, but no other data could be found.

***Placogorgia* sp. A**

**Material examined.** ~5 lots. **USA, California** – 1 colony, in fragments; Orange County, Oceanside, 33°10'29"N, 117°23'09"W, Station IP-B1046, caught in shark net, 138 m; coll. B Brophy, 1971; SBMNH 422969 [DH 844 = SBMNH-51; dry]. –multiple fragments; Los Angeles County, off Santa Monica, South Banks, 34°00'21"N, 118°29'57"W, 82–100 m; coll. Pacific BioMarine, commercial trawl, 10 October 1978; SBMNH 422970 [wet]. –1 colony fragment; Ventura County, California Channel Islands, NW edge of Hueneme Canyon, 34°02'20"N, 119°18'07"W, 145 m; coll. NMFS Dive 32, 23 September 2011; SBMNH 235579 [dry]. –fragment; Ventura County, California Channel Islands, slightly NE of East Anacapa, 34°02'20"N, 119°18'13"W, 140 m; coll. M Love from submersible ‘Delta’, 14 October 2005; with his number: A6689; SBMNH 422968 [wet]. **MEXICO, Baja California Norte (Pacific Coast)** – multiple fragments; 5.5 miles S of Islas San Benito, fine green sand, coarse grey sand, 28°13'18(55)” N, 115°33(35)’15(05)” W (end), 120–147 m; coll. R/V ‘Velero III’, station 1251-41, 26 February 1941; SBMNH 422966 [wet].

**Other material examined** – 1 piece; USA, California, Orange County, northern edge of San Gabriel Canyon, off Huntington Beach, trawl, ~33°33'49"N, 118°08'40"W, 113 m; coll. DB Cadien, EMAP Voucher, Survey SCBPP, southern California Bight, Station 1476 (ID by J Ljubenkov, not collected by MEC), 29 July 1994 [wet].

## Appendix 3

**Table A1.** Contrasts and comparisons of key “red whip” species and/or species of the genus *Swiftia*, as represented in SBMNH collection.

Red Whip species	Location, S to N	Location Depth	Colony Branching	Colony Color	Polyp Spacing	Polyp Height	Sclerite Color	Sclerite Form	Sclerite Size
* Leptogorgia flexilis *	Magdalena Bay, Baja to San Diego, CA	11 meters to ?	Thin, drooping branches; highly branched colony	Red/pink to tan/beige Polyps white to very pale orange	No more than 1 mm	No more than 1 mm	Bright Salmon	Spindles & Capstans	.03–.09 mm
* Leptogorgia chilensis *	N of Magdalena Bay to Santa Cruz Is., CA	Approx. 15–80 m	Thin branches; moderately branched	Orange-red Polyps white	1 mm	Generally, almost flush	Bright Salmon	Spindles & Capstans	.03-.05 mm
Red Whip (?”Transitional/Regional Endemic”)	San Diego, CA to off Oregon coast	?20–2,000 meters	Moderate thickness to branches; slightly branched to not branched	Orange-red Polyps white	Varies from 1 to 2 mm	Generally, from flush to nearly 1 mm; rarely taller	Salmon	Spindles & Dbl. Spinds.	Approx. 0.1 mm
Red Whip (?”Transitional/Regional Endemic”)	San Diego, CA to off Oregon coast; possible extension to WA coast	Approx. 12–150 m	Moderately thin branches, whip-like; slightly branched	Orange-red Polyps white (?pale pink)	Varies from 1 to 2 mm	Generally, consistently flush, rarely taller; on some, prominent	Salmon	Spindles & Dbl. Spinds.	Approx. 0.1 mm
“?*Swiftia* Transitional/ Regional species”	N Los Angeles County to Point Conception	Approx. 104–173 m	Single branches; also slightly branched (if so, dichotomous)	Bright red to salmon-pink Polyps white	Less than 1 mm	Approx. 1 mm	Salmon	Spindles; very few Capstans, Dbl. Spinds. or Rods	From 0.04 mm to nearly +.16 mm
* Swiftia simplex *	N Los Angeles County to Alaska	200–900 m	Single branches; sometimes slightly branched	Pinkish-red (Brick-red) Polyps pinkish-red	No more than 2 mm	Approx. 1 mm	Pinkish-red (Brick color) Rods orange	Spindles, Capstans, Rods and Dbl. Spinds.	.1–.3 mm
* Chromoplexaura marki *	Point Conception to Cape Mendocino, CA (?further north to WA state, on to Alaska)	20–60 m; possibly deeper (to 600 m)	Single branches; sometimes slightly to moderately branched	Bright red, orange, even pinkish Polyps white or colored	2 mm	Nearly flush to 2 mm	Salmon to reddish	Spindles, Capstans, Ovals and Dbl. Spinds; NO Rods	0.05 mm to ≅ 0.2 mm
* Swifita spauldingi *	Monterey Bay, CA to off Washington coast (?further north to Alaska)	40 to at least 300 m	Moderate branch thickness; branched to some degree	Orange-red Polyps white or very pale pink	about 1 mm	Nearly flush to often very conspicuous, rounded	Salmon to pinkish- orange; some yellow; Rods orange	Spindles, Capstans, Rods and Dbl. Spinds.	About 0.1 mm

Polyp Height: includes both calyx and actual polyp.
